# Single-cell transcriptomic profile of human pulmonary artery endothelial cells in health and pulmonary arterial hypertension

**DOI:** 10.1038/s41598-021-94163-y

**Published:** 2021-07-19

**Authors:** Kewal Asosingh, Suzy Comhair, Lori Mavrakis, Weiling Xu, Dean Horton, Ian Taylor, Svyatoslav Tkachenko, Bo Hu, Serpil Erzurum

**Affiliations:** 1 Departments of Inflammation and Immunity, Lerner Research Institute and Respiratory Institute, Cleveland Clinic, 9500 Euclid Ave, NB20, Cleveland, OH 44195 USA; 2 Departments of Quantitative Health Sciences, Lerner Research Institute and Respiratory Institute, Cleveland Clinic, Cleveland, OH USA; 3 Departments of Flow Cytometry Core, Lerner Research Institute and Respiratory Institute, Cleveland Clinic, Cleveland, OH USA; 4 Departments of Genomics Core, Lerner Research Institute and Respiratory Institute, Cleveland Clinic, Cleveland, OH USA; 5 BD Life Sciences: Informatics, Ashland, OR USA

**Keywords:** Mechanisms of disease, Cell biology, Health occupations, Translational research

## Abstract

Pulmonary arterial hypertension (PAH) is an insidious disease characterized by severe remodeling of the pulmonary vasculature caused in part by pathologic changes of endothelial cell functions. Although heterogeneity of endothelial cells across various vascular beds is well known, the diversity among endothelial cells in the healthy pulmonary vascular bed and the pathologic diversity among pulmonary arterial endothelial cells (PAEC) in PAH is unknown and previously unexplored. Here single-cell RNA sequencing technology was used to decipher the cellular heterogeneity among PAEC in the human pulmonary arteries isolated from explanted lungs from three patients with PAH undergoing lung transplantation and three healthy donor lungs not utilized for transplantation. Datasets of 36,368 PAH individual endothelial cells and 36,086 healthy cells were analyzed using the SeqGeq bioinformatics program. Total population differential gene expression analyses identified 629 differentially expressed genes between PAH and controls. Gene Ontology and Canonical Ingenuity analysis revealed pathways that are known to be involved in pathogenesis, as well as unique new pathways. At the individual cell level, dimensionality reduction followed by density based clustering revealed the presence of eight unique PAEC clusters that were typified by proliferative, angiogenic or quiescent phenotypes. While control and PAH harbored many similar subgroups of endothelial cells, PAH had greater proportions of angiogenic and proliferative subsets. These findings identify that only specific subgroups of PAH PAEC have gene expression different than healthy PAEC, and suggest these subpopulations lead to the pathologic functions leading to remodeling.

## Introduction

Pulmonary artery hypertension (PAH) is a deadly cardiopulmonary disease characterized by profound structural changes to the pulmonary artery wall^1^. Pulmonary artery endothelial cells (PAEC) are causally linked the vascular remodeling in PAH^2–6^. For example, apoptosis-resistant proliferation and dysregulated metabolism in PAEC are key features of PAH^4–7^. Although endothelium exhibits a marked heterogeneity in structure and function^8–10^, little is known about the single-cell transcriptomic heterogeneity of the pulmonary vasculature. The pulmonary vasculature is distinct from other vascular beds; it is a low pressure vascular bed and accommodates the complete venous return from the systemic vascular bed. Because endothelial remodeling in PAH is patchy^11^, PAEC isolated from explanted lung contain a mixture of cells from healthy and affected areas. Analysis of these cells in bulk cultures may mask phenotypically altered PAEC subsets. Here, we hypothesized that the transcriptomic diversity of PAEC isolated from healthy and PAH lungs would differ and that PAH would have specific expansion of pathological phenotypes of cells. To test this, we evaluated PAH and control PAEC by methodologies to identify cell clusters and their phenotypes. Eight distinct clusters of endothelial cells were identified from healthy and PAH lungs. Transcriptomic profiles identified expansion of clusters with proliferative and angiogenic gene signatures in PAH.

## Results

### Differential gene expression between total populations of control and PAH PAEC

**Figure 1:**
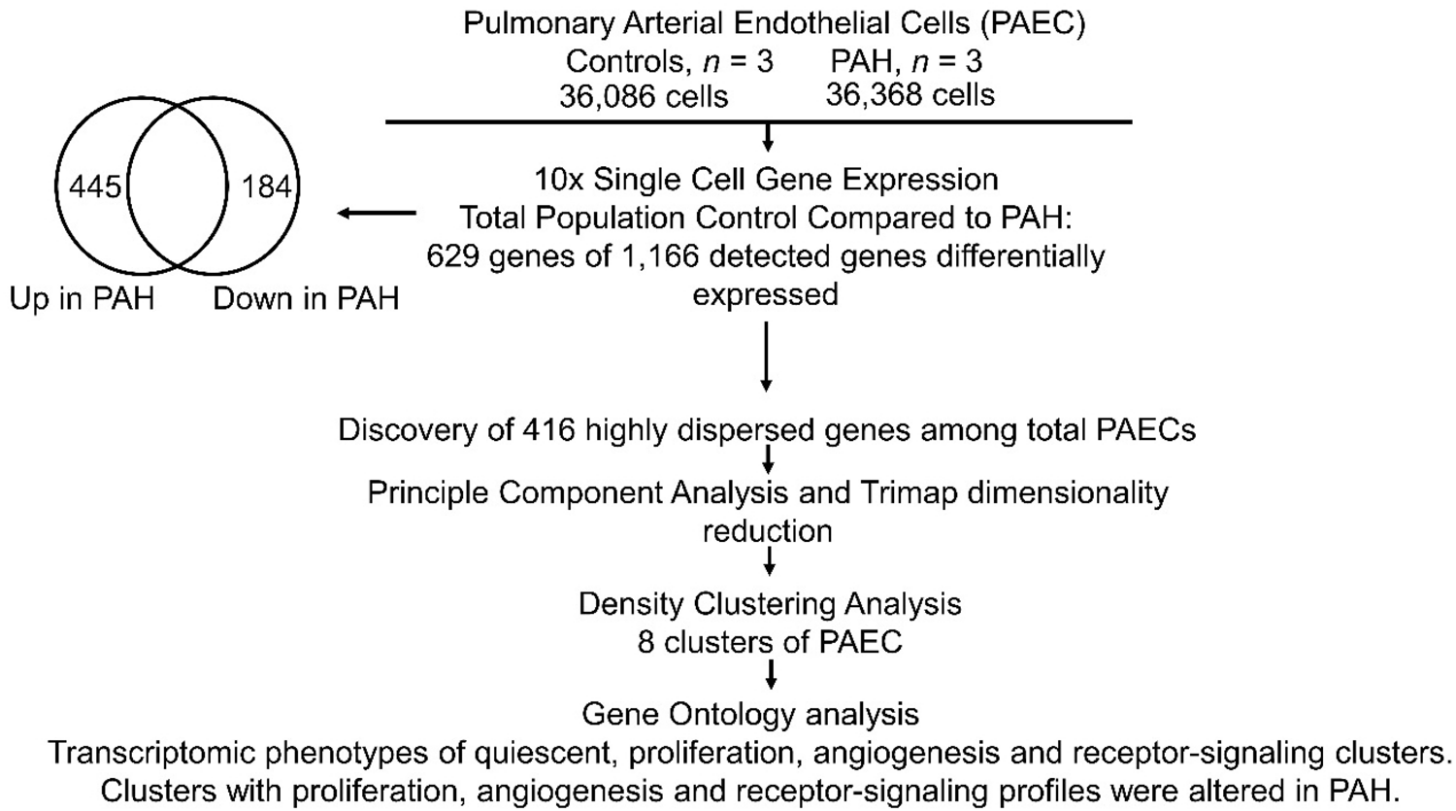
Overall work flow differential gene expression and clustering analysis. 10 × Single-Cell Omics was performed on PAEC derived from 3 control and 3 PAH lungs. The total number of cells in each sample was 36 K. Global gene expression analysis between the total populations of control and PAH cells revealed that 629 genes from 1,116 detected genes were differentially expressed. Among these, 445 genes were upregulated and 184 were downregulated in PAH. 416 highly dispersed genes were found in the total control and PAH population. Principle Component Analysis followed by Trimap dimentionality reduction was performed to depict the high-dimentional data in a two-dimentional space. Density Clustering Analysis identified 8 clusters of cells among control and PAH PAEC. Differential gene expression analysis of clusters in each sample, followed by gene ontology analysis of the differentially expressed genes showed that there were 4 transcriptomic profiles among the 8 clusters of PAECs. Three PAH clusters had an altered profile compared to the corresponding control cluster.

**Figure 2:**
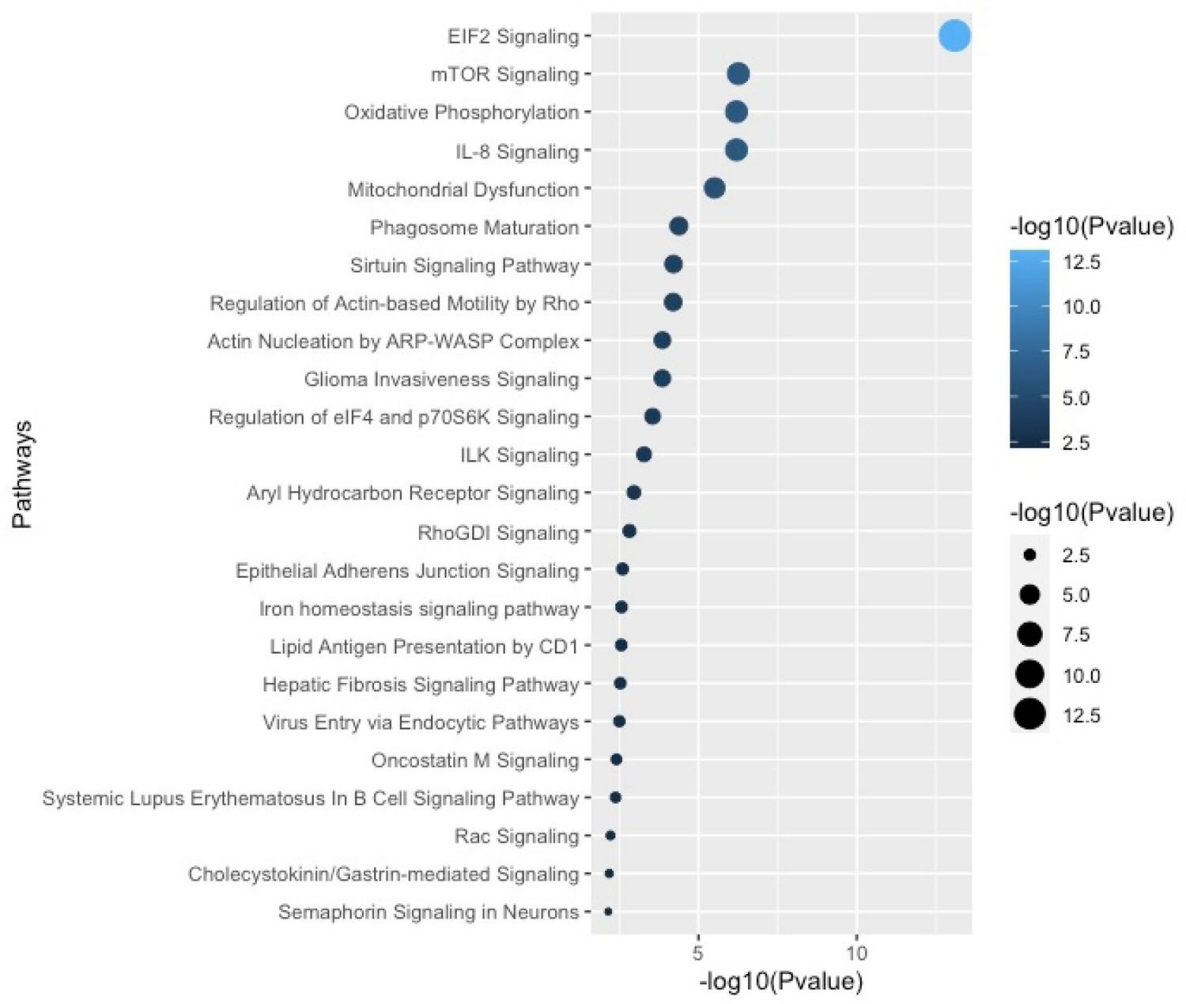
Ingenuity pathway analysis of genes differentially expressed between the total population of control and PAH PAECs. Differential expression analyses revealed 629 genes. These were used for canonical pathway ingenuity analyses.

PAEC were isolated with purities > 95% as demonstrated by cell surface expression of endothelial cell specific markers CD31 and VEGFR2. Final count in the single-cell RNA-seq dataset was 72,454 cells in total, with 36,368 PAH endothelial cells (n = 3; 10,389; 13,055; 12,924 cells per individual samples) and 36,086 healthy PAEC (n = 3; 9,589; 11,482; 15,015 cells per individual samples). Mean library size and number of genes expressed per cell were determined to be 3,166 and 1,166 respectively. Quality control of genes started by first eliminating outliers based on genes expressed per cell, library size per cell, and total reads per gene (8825 features). The top 416 highly dispersed transcripts were selected for initial dimensionality reduction and clustering analysis. (Supplemental Fig. S1). Initial Principal Component Analysis (PCA) was followed by non-linear dimensionality reduction including t-distributed stochastic neighbor embedding (tSNE), Uniform Manifold Approximation and Projection (UMAP), and TriMap^12^. All these dimensionality reduction algorithms confirmed that datasets segregated by sequencing run (i.e. by sample), rather than by cell type. Based on this finding we determined that batch effects would be a confounding factor in the analysis. In order to correct for this, Mutual Nearest Neighbors (MNN) batch effect correction was applied to the data matrix using the Batchelor algorithm^13^, followed by a fresh round of PCA, and TriMap dimensionality reduction on MNN batch effect corrected data. (Supplemental Fig. S2). In these initial analyses, we performed traditional differential gene expression analyses of total cell populations, which revealed that 629 genes were differentially expressed between the two populations of PAH and controls. Gene ontology analysis revealed major pathways involving the differentially expressed genes. The overall workflow for differential gene expression analysis is summarized in Fig. 1. Table 1 summarizes endothelial and mitochondrial pathways. Genes upregulated in PAH were enriched in pathways involved in mitochondrial function. Downregulated genes also included some in mitochondrial function and others in angiogenic pathways. Ingenuity pathway analysis was also performed on the differentially expressed genes to identify canonical pathways (Fig. 2). Several pathways known to play a role in PAH were seen in these analyses, confirming that our cell populations were representative of PAH and healthy pulmonary vascular beds as previously published^5,11,14–17^. These included upregulation of Eukaryotic Initiation Factor (EIF) 2 signaling, which was the most significant pathway upregulated among PAH PAEC^14^. Mitochondrial pathways, another well-established dysfunction in PAH, was also increased among PAH PAECs^5,14^. All other pathways identified by the differential gene expression have not been reported in PAH (Fig. 2). The complete list of differentially expressed genes is provided in Supplementary Tables S1 and S2.

**Table 1:** Differential expression of endothelial and mitochondrial pathways by ontology analysis.

Gene set name	FDR q-value	Up or down in PAH
ATP_METABOLIC_PROCESS	1.41E−16	Up
MITOCHONDRION_ORGANIZATION	1.44E−16	Up
OXIDATIVE_PHOSPHORYLATION	2.49E−15	Up
ELECTRON_TRANSPORT_CHAIN	2.99E−15	Up
OXIDATION_REDUCTION_PROCESS	4.87E−15	Up
ATP_SYNTHESIS_COUPLED_ELECTRON_TRANSPORT	4.07E−14	Up
CELLULAR_RESPIRATION	2.62E−13	Up
RESPIRATORY_ELECTRON_TRANSPORT_CHAIN	6.87E−13	Up
MITOCHONDRIAL_ELECTRON_TRANSPORT_NADH_TO_UBIQUINONE	6.97E−13	Up
ENERGY_DERIVATION_BY_OXIDATION_OF_ORGANIC_COMPOUNDS	1.26E−12	Up
NADH_DEHYDROGENASE_COMPLEX_ASSEMBLY	4.90E−12	Up
MITOCHONDRIAL_RESPIRATORY_CHAIN_COMPLEX_ASSEMBLY	5.96E−12	Up
MITOCHONDRIAL_TRANSLATIONAL_TERMINATION	2.96E−11	Up
MITOCHONDRIAL_TRANSLATION	9.39E−09	Up
MITOCHONDRIAL_GENE_EXPRESSION	9.13E−08	Up
TUBE_DEVELOPMENT	3.66E−11	Down
TUBE_MORPHOGENESIS	1.19E−10	Down
BLOOD_VESSEL_MORPHOGENESIS	5.99E−10	Down
ENDOTHELIAL_CELL_MIGRATION	1.43E−08	Down
OXIDATIVE_PHOSPHORYLATION	3.12E−07	Down
ENDOTHELIUM_DEVELOPMENT	3.10E−05	Down
ATP_SYNTHESIS_COUPLED_ELECTRON_TRANSPORT	4.83E−05	Down
RESPIRATORY_ELECTRON_TRANSPORT_CHAIN	1.30E−04	Down
REGULATION_OF_ENDOTHELIAL_CELL_MIGRATION	1.54E−04	Down
ATP_METABOLIC_PROCESS	1.88E−04	Down
BLOOD_VESSEL_ENDOTHELIAL_CELL_MIGRATION	2.21E−04	Down
POSITIVE_REGULATION_OF_ENDOTHELIAL_CELL_MIGRATION	2.35E−04	Down
SPROUTING_ANGIOGENESIS	2.52E−04	Down

*FDR* false discovery rate.

### Clustering of control and PAH PAEC

**Figure 3:**
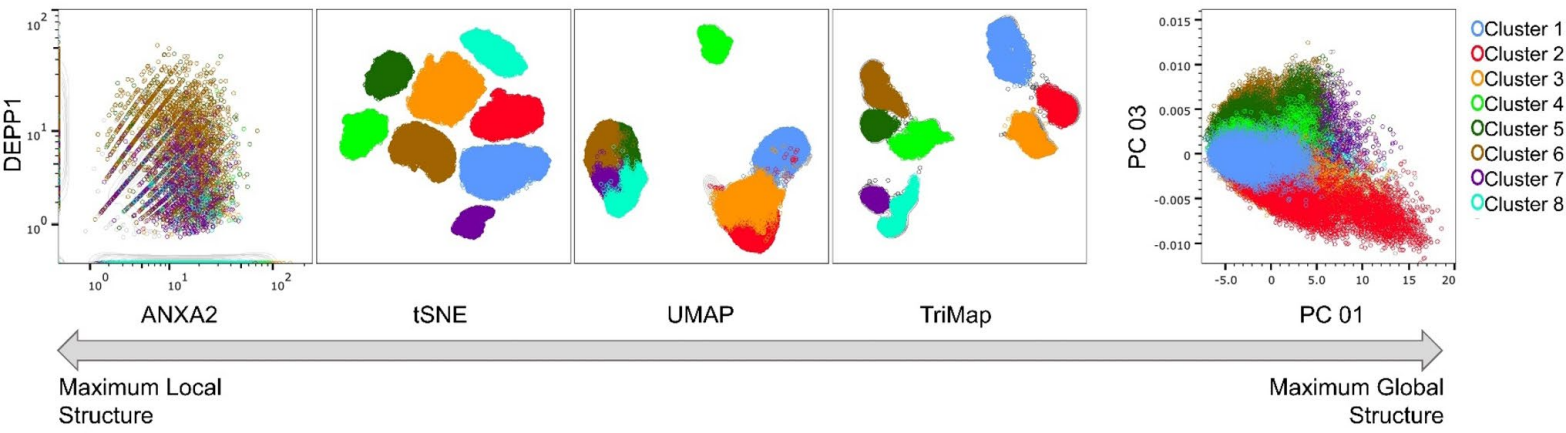
Dimensionality reduction using all genes in all cells. Dimensionality reduction summarizes the heterogeneous populations in a high dimensional space in a two-dimensional embedding. The DEPP1/ANXA2 plot depict the expression of the two genes in the different clusters in a two-dimensional embedding. The tSNE, UMAP, TriMAP and PC plots depict the expression of all genes in higher dimensional space in a two-dimensional embedding. Thus, from left to right the data depicts maximum local structure (for example the needles on a pine tree) to maximum global structure (for example the clusters of pine trees in a pine forest). Eight clusters were identified by visual inspection of the TriMap space.

**Figure 4:**
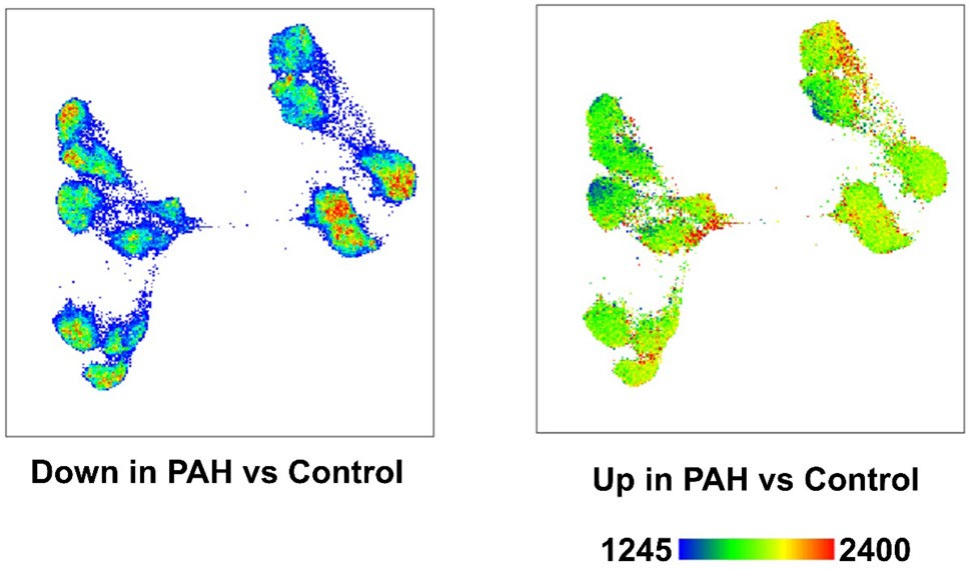
Distribution of the 629 differentially expressed genes across clusters. TriMap space showing genes down (left figure) and up (right figure) regulated in PAH. The differentially expressed genes did not localize in a single cluster, but were spread across all clusters.

**Figure 5:**
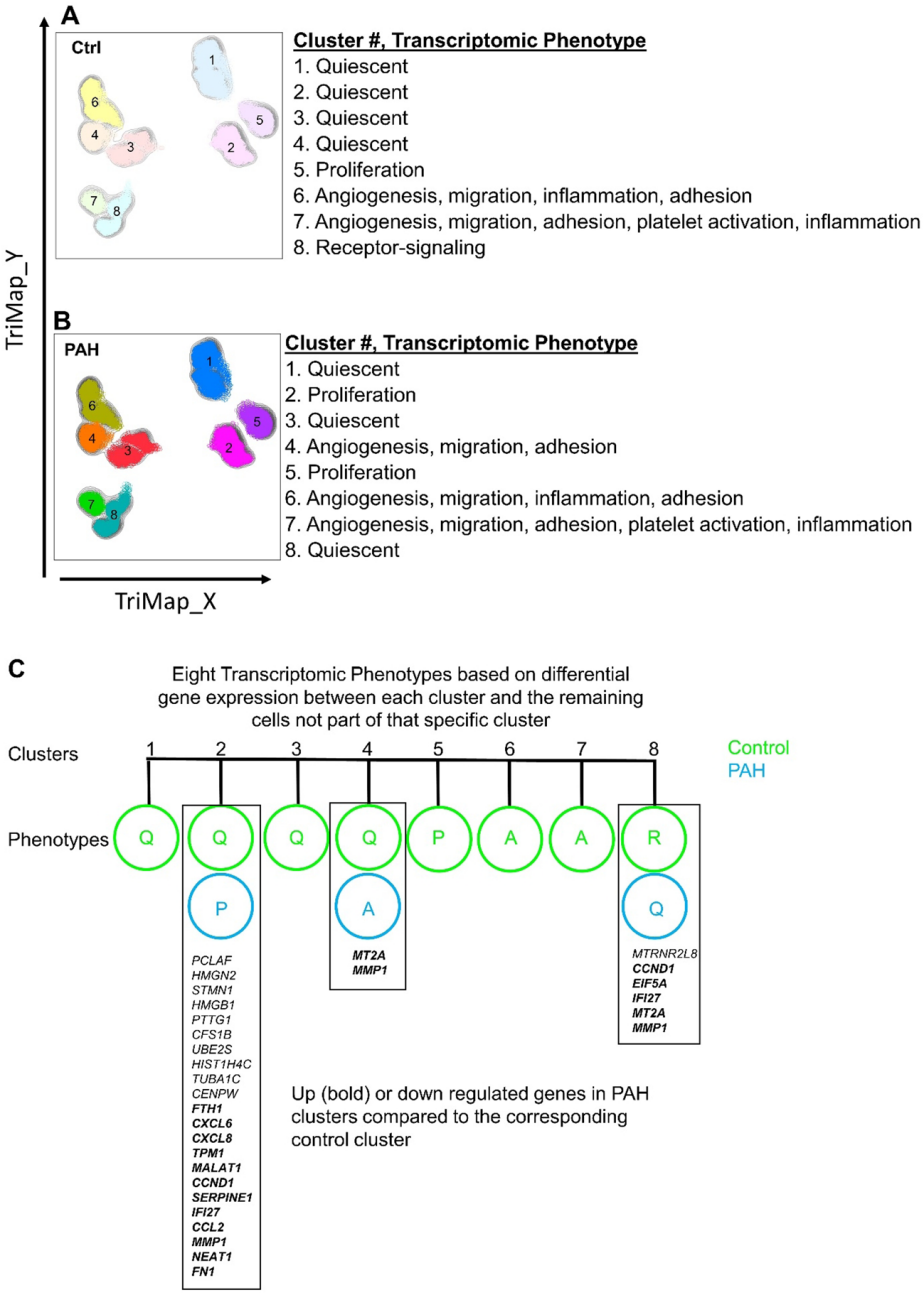
Segregation of control and PAH PAEC clustering. TriMap clustering was performed on the total PAEC population. Control and PAH cells were then segregated based on their unique sample IDs. Differential gene expression analysis was performed between each cluster and the remaining cells, not part of that specific cluster, within control and PAH PAEC populations. Gene Ontology Term Enrichment Analysis was then performed on these differentially expressed gen-sets. Each cluster was named based on key identified pathways (**A**,**B**). Clusters 2, 4, 8 were different between control and PAH. Genes differentially expressed in PAH clusters compared to the corresponding control cluster are shown (Up regulated genes in bold) *Q* quiescent; *P* proliferation; *A* angiogenesis, and *R* receptor-signaling(**C**).

Clustering of control and PAH PAECs was first performed using a nonbiased approach. Dimensionality reduction in single cell analyses summarizes heterogeneous populations in a high dimensional space in a two-dimensional embedding. tSNE, UMAP and TriMAP dimensionality reduction were applied (Fig. 3). The TriMap algorithm was used because it gave an improved preservation of the global data structure in the two-dimensional space^12^. Three clustering approaches, PhenoGraph, Kmeans and density clustering were used to identify PAEC subsets. A minimum number of clusters found by these methods was 8 (Supplemental Fig. S3). Because the density clustering gave the clearest separation among the clusters we proceeded with this clustering approach to explore the expression patterns that defined the 8 subsets. The distribution of the differentially expressed genes between PAH and controls found in the total population (629 genes) was assessed for possible localization of differential expression in any specific clusters Fig. 4. In fact, the total population differentially expressed genes did not localize to any single cluster. We next investigated the specific phenotypes of cells in each cluster. For this purpose, we performed differential gene expression analysis between each cluster as compared to the remaining cells not part of that specific cluster, within control or PAH populations. The complete lists of these genes are shown in Supplemental Tables S3–S6. Gene Ontology, a bioinformatics platform providing functional information about gene products and biological processes in which gene sets are involved^18,19^, was utilized to gauge functionality and naming of the clusters (Fig. 5A,B).

Cluster 1 in control and PAH PAEC exhibited genes primarily all down-regulated relative to the remaining clusters. The majority of the cells in cluster 1 were involved in cell-cycle and cell proliferation, for example centromere protein F (*cenpf*), cell division cycle 25B (*cdc25b*), cyclin F (*ccnf*), regulator of cell cycle (*rgcc*) and marker of proliferation Ki-67 (*mki67*). Based on the down-regulated nature profile of this cluster, it was assigned the name of quiescent cluster.

A cluster 2 cell group was identified in controls and PAH, but their gene expression was dissimilar. In control PAEC, cluster 2 genes involved in angiogenesis, cell migration and proliferation were all down-regulated. These genes included kinase insert domain receptor (*kdr*), regulator of cell cycle (*rgcc*), platelet and endothelial cell adhesion molecule 1 (*pecam*)*,* placental growth factor (*pgf*)*,* C-X-C chemokine receptor type 4 (*cxcr4*)*,* Angiopoietin-like 4 (*angptl4*)*,* G1/S-specific cyclin-D2 (*ccnd2*)* and,* Chemokine (C–C motif) ligand 14 (*ccl14*)*.* The transcriptomic profile suggests that this healthy PAEC cluster has a quiescent phenotype. In contrast, PAH PAEC cluster 2 had both down- and up-regulated genes. Down-regulated genes, such as *pgf, kdr,* fibronectin 1 (*fn1*)* and* C–C motif chemokine ligand 2 (*ccl2*)*,* are mainly involved in new blood vessel formation. Eight genes were strikingly up-regulated in PAH cluster 2, including denticleless e3 ubiquitin protein ligase homolog (*dtl*)*,* minichromosome maintenance complex component 4 (*mcm4*) and minichromosome maintenance 10 replication initiation factor (*mcm10*). Gene Ontology term enrichment analyses showed that these genes are involved in DNA replication, cell-cycle checkpoint, DNA strand elongation and cell proliferation. Thus cluster 2 in control PAEC contains quiescent cells, but in PAH contain cells with a proliferative phenotype.

Cluster 3 in both control and PAH were characterized by decreased expression of genes in cell-cycle and cell proliferation pathways, including *cenpf, cdc25b, cenpw* (centromere protein W)*, pclaf* 9 (pcna clamp associated factor)*,* and *cdkn3* (cyclin dependent kinase inhibitor 3)*.* Therefore, cluster 3 in both PAH and controls were assigned the term of quiescent phenotype.

A cluster 4 was identified in both PAH and control PAEC but the clusters were different in their transcriptomic profiles. Cluster 4 in control PAEC had 142 transcripts that were down-regulated relative to the other clusters. The genes were from wide-ranging pathways, including mitotic cell cycle, cell division, chromosome organization and segregation, spindle organization and microtubule cytoskeleton organization. Based on this transcriptome, cluster 4 among control PAEC was termed quiescent. In contrast, although some cell proliferation genes were down-regulated in PAH cluster 4, 23 genes in angiogenesis, migration and adhesion pathways were up-regulated, such as (claudin 4 (*cldn5*)*, pgf, cxcr4,* nidogen 2 (*nid2*)*, and ccl14*)*.* This profile suggests that PAH cluster 4 has a unique angiogenic phenotype not found in the control PAEC cluster 4.

The majority of the differentially expressed genes of cluster 5 were upregulated in both control and PAH cells. The genes upregulated in this cluster control cell proliferation, such as *cenpf,* cytoskeleton-associated protein 2 (*ckap2*)*,* kinesin family member 15 (*kif15*)*,* kinesin family member 18a (*kif18a*)*, mik67,* serine/threonine-protein kinase 2 (*nek2*)* and* kinetochore protein (*spc25*)*.* This cluster is thus active in proliferation and was accordingly named Proliferative cluster.

Cluster 6 was similar among control and PAH, with some down-regulated genes in cell cycle pathways, but many upregulated genes in processes of angiogenesis, migration, inflammation and cell adhesion (*kdr, cxcr4,* angiopoietin 2 (*angpt2*)*, pgf,* platelet derived growth factor subunit B (*pdgfb*), *nid2,* jagged canonical notch ligand 2 (*jag2*), OX-2 membrane glycoprotein (*cd200*), *ccl14*). This cluster was accordingly considered as an angiogenic subset in both control and PAH.

Similar to cluster 6, cluster 7 among control and PAH PAEC had reduced expression of cell proliferation genes (cell division cycle-associated 3 (*cdca3*)*,* DNA topoisomerase II alpha (*top2a*)*, and mki67*) relative to other clusters*.* In both control and PAH, genes regulating angiogenesis, migration, adhesion, platelet activation and inflammation were overexpressed, including (*kdr,* endoglin (*eng*)*, pecam1,* matrix metallopeptidase 2 (*mmp2*)*,* (matrix metallopeptidase 12 (*mmp12*)*, cldn5, pgf,* cd93 molecule (*cd93*)*,* protocadherin 9 (*pcdh9*)*,* clusterin (*clu*)*,* heparin binding egf like growth factor (*hbegf*)*, and* glycophorin C (*gypc*)). The clusters were named consistent with these phenotypes of angiogenesis, migration and inflammation.

Control and PAH Cluster 8 had cells with down-regulation of genes mainly involved in cell proliferation. While there were no genes upregulated in PAH cluster 8, control PAEC cluster 8 had upregulated expression of genes controlling the enzyme-linked-receptor-protein-signaling pathways inhibin subunit beta A (*inhba*)*,* follistatin like 3 (*fstl3*)*,* zinc finger protein 703 (*znf703*)*,* hedgehog interacting protein (*hhip*)*,* eph receptor B6 (*ephb6*)*,* integrin subunit beta 3 (*itgb3*)* and* plasminogen activator, tissue type (*plat*)*.* These clusters were therefore named Receptor-signaling in control cluster 8 and Quiescent in PAH cluster 8.

Differential gene expression analysis was subsequently performed to compare transcriptomic profiles of control and PAH clusters (2, 4 and 8) (Fig. 5C). Genes down-regulated in PAH cluster 2 relative to control cluster 2 were involved in microtubules, spindle and centromere formation (*tuba1b, tuba1c and cenp10*)*.* Upregulated genes regulate intracellular iron storage (*fth1*)*,* chemoattractant of inflammatory cells know to have essential roles in PAH such as neutrophils, monocytes, T-cell and dendritic cells (*cxcl6, cxcl8 and ccl2*)^20–22^*.* In clusters 4 and 8, *mt2a,* a member of the metallothionein family controlling intracellular metabolism of essential metals^23^, and *mmp1 *(matrix metallopeptidase 1) were overexpressed. In cluster 8, humanin-like pepdide 8 (*mtrnr2l8*), a mitochondrial genome encoded gene with receptor activity regulator activity, was down-regulated^24^. Importantly, the majority of genes differentially expressed between corresponding control and PAH clusters were not identified in the global differential gene expression analysis with the total control and PAH cells. Only *HIST1H4C* (downregulated) and *FTH1, CXCL6, CCND1 IFI27 and CCL2* (upregulated) were found in the analysis of the total populations.

**Figure 6:**
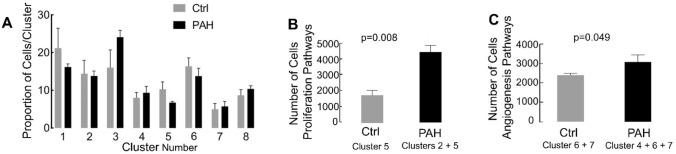
Increased angioproliferative cells in PAH. The mean frequency of each cluster, calculated based on the cluster frequency in the individual samples, was similar between control and PAH (**A**). The total number of cells in the clusters enriched in proliferation pathways (cluster 2 in control, and clusters 2 and 3 in PAH), was significantly increased in PAH (**B**). The total number of cells in clusters enriched in angiogenic pathways (clusters 6 and 7 in control, and clusters 5, 6, and 7 in PAH), was significantly higher in PAH (**C**).

The proportion of cells in each individual cluster was similar between control and PAH (Fig. 6A). Comparison of the total number of cells enriched in proliferation pathways between conditions, (control cluster 5 *vs*. PAH clusters 2 + 5) showed more proliferative cells in PAH (Fig. 6B). Similarly, the total number of cells in an angiogenesis pathway was significantly higher in PAH (control clusters 6 + 7 *vs* PAH clusters 4 + 6 + 7) (Fig. 6C). Overall, PAH PAEC contained more proliferating and angiogenic cells due to a phenotypic change in specific clusters. In a biased approach, we also evaluated only the top ten differentially overexpressed genes in each cluster in control (Table 2) and PAH samples (Table 3). These genes revealed similar transcriptomic profiles as the Gene Ontology term enrichment.

**Table 2:** Top ten upregulated genes in control clusters.

Cluster	Parameter	Fold-change	FDR q-value
2	TOP2A	28.61185732	3.55E−231
2	MKI67	26.27793419	0
2	NEK2	24.64180751	8.94E−89
2	CENPF	23.20713437	1.3024430098599E−317
2	NUF2	22.46846048	9.23E−136
2	SPC25	21.06233128	1.27E−129
2	KIF15	20.71534477	1.20E−67
2	PSRC1	20.18222113	1.46E−64
2	KIF18A	20.14262227	1.11E−47
2	CKAP2L	19.99418063	2.34E−112
6	RASGRP3	10.13670085	6.27E−88
6	CXCR4	8.520098363	2.00E−299
6	CD200	5.158937263	1.57E−17
6	CSGALNACT1	5.173452832	5.44E−116
6	CHST1	10.93093523	4.56E−166
6	DEPP1	9.216096628	2.72E−259
6	MGP	6.676951416	5.00E−138
6	RGCC	5.174688626	2.54E−231
6	NID2	5.645609732	2.07E−26
6	PLA2G4C	6.073089562	5.78E−105
7	HLX	3.308916449	4.14E−16
7	NOTCH4	2.994194879	1.03E−31
7	AKAP12	3.897821175	7.75E−57
7	PEG10	3.317471895	3.69E−28
7	APLN	3.051646077	8.94E−49
7	CNTNAP3B	3.478569887	5.03E−47
7	PGF	3.46250528	6.01E−113
7	YPEL2	2.966003011	1.13E−21
7	LIPG	3.10185442	1.08E−09
7	PDGFB	2.983750339	8.70E−17
8	PTX3	2.383583031	4.29E−82
8	DDIT4L	2.344600929	2.83E−11
8	HHIP	2.314530069	7.29E−125
8	TGFBI	2.439371111	9.35E−25
8	PLAT	2.945899196	2.15E−40
8	FRMD3	2.432868258	6.78E−14
8	CLIC3	2.445455159	0.002293661
8	ANKRD1	2.490652973	5.36E−48
8	ITGB3	2.343396353	6.43E−28
8	FSTL3	2.403705182	9.46E−15

Clusters 1 and 3–5 among control PAEC exhibited mostly down-regulated genes. The set of top ten overexpressed genes in control 2 cluster was enriched in genes involving cell proliferation, including *cenpf, ckap2l, kif15, kif18a, mik67, nek2 and spc25.* Control cluster 6 had increased expression of top ten genes regulating immune cell—endothelial interaction and new vessel formation such as *cd200,* Carbohydrate Sulfotransferase 1 (*chst1*), *cxcr4,* matrix Gla protein (*mgp*)*, and* Ras guanyl-releasing protein 3 (*rasgrp3*). Sprouting and migratory gene signatures, A-kinase anchoring protein 12 (*akap12*), aplin (*apln*)*,* H2. 0 Like Homeobox (*hlx*), Neurogenic locus notch homolog 4 (*notch4*), *and pdgfb* were enriched in control cluster 7. Highly expressed genes in control cluster 8 were characteristic of inflammation and angiogenesis, including *fstl3, hhip, itgb3, ptx3 and tgfb1.*

**Table 3:** Top ten upregulated genes in PAH clusters.

Cluster	Gene ID	Fold-change	FDR q-value
2	TOP2A	23.31466095	3.18E−299
2	UBE2C	20.81621634	0
2	KIF18A	20.80020025	3.16E−46
2	MKI67	17.37921703	0
2	NEK2	17.1630725	2.83E−84
2	PSRC1	17.09632113	2.95E−71
2	NUF2	16.73112233	1.31E−152
2	CKAP2L	15.91362108	4.26E−133
2	CENPF	15.54628052	3.5E−316
2	SPC25	14.05552696	4.99E−104
3	DTL	2.864467417	1.43E−25
3	MCM4	2.370515087	2.82E−43
3	MCM10	2.884836554	8.37E−26
3	TUBA1B	2.374727347	0
3	GINS2	2.519801484	3.60E−63
3	CDT1	2.22514212	5.08E−37
3	E2F1	2.985704965	1.41E−53
3	MCM5	2.339736103	5.60E−47
5	TNFSF10	3.253200019	9.59E−08
5	PPP1R16B	3.177574014	5.81E−16
5	CSGALNACT1	2.985941539	1.18E−24
5	CXCR4	2.983789305	3.01E−52
5	TM4SF18	2.710871286	8.57E−18
5	RASGRP3	2.68766549	6.67E−09
5	DEPP1	2.676648883	1.10E−38
5	CCL14	2.641992743	1.25E−05
5	RGCC	2.553964856	1.94E−59
5	SULF2	2.544644983	4.64E−30
6	CHST1	12.52724927	3.06E−162
6	DEPP1	10.92164641	2.61E−257
6	CXCR4	9.002071006	9.68E−288
6	RASGRP3	8.64920468	3.82E−69
6	VWF	7.821002651	6.27E−191
6	PPP1R16B	7.191421822	2.63E−73
6	NID2	6.666894279	3.47E−26
6	CSGALNACT1	6.174138021	2.42E−106
6	HOXB5	5.980496397	4.69E−14
6	TNFSF10	5.196597822	1.09E−26
7	PLA2G4C	3.152371554	8.22E−20
7	ITGA10	3.121176978	2.22E−19
7	CCL14	3.023824847	1.09E−06
7	FRMD3	2.987223157	1.90E−13
7	AKAP12	2.924917691	1.01E−52
7	CNTNAP3B	2.845281525	3.06E−54
7	CDC42EP5	2.82167109	3.26E−33
7	HSPG2	2.7813723	6.94E−121
7	PGF	2.779502026	2.09E−124
7	CAMK2N1	2.771007913	1.09E−19

Clusters 1 and 8 in PAH exhibited mostly down-regulated genes. Similar to control cluster 2, PAH cluster 2 overexpressed genes involved in cell proliferation. Interestingly, PAH cluster 3 was also enriched in overexpression of genes involved in cell proliferation (*cdt1*, DNA replication complex GINS protein (*gins2*)*, mcm10, mcm4, and mcm5*). PAH cluster 4 did not have any upregulated genes. In cluster 5 contained genes reported in aberrant angiogenesis (*ccl14, cxcr4, rasgrp3 and* Extracellular sulfatase Sulf-2 (*sulf2*)). Similar to control cluster 6, PAH cluster 6 was enriched in genes regulating immune cell—endothelial interaction and new vessel formation. Genes upregulated in PAH cluster 7 were compatible with an angiogenic phenotype (A-Kinase Anchoring Protein 12 (akap12); ccl14; pgf and Cell Division Cycle 42 Pseudogene 5 (cdc42p5)).

### Expression of BMPR2, TGF-β signaling genes and endothelial-to-mesenchymal transition markers

**Figure 7:**
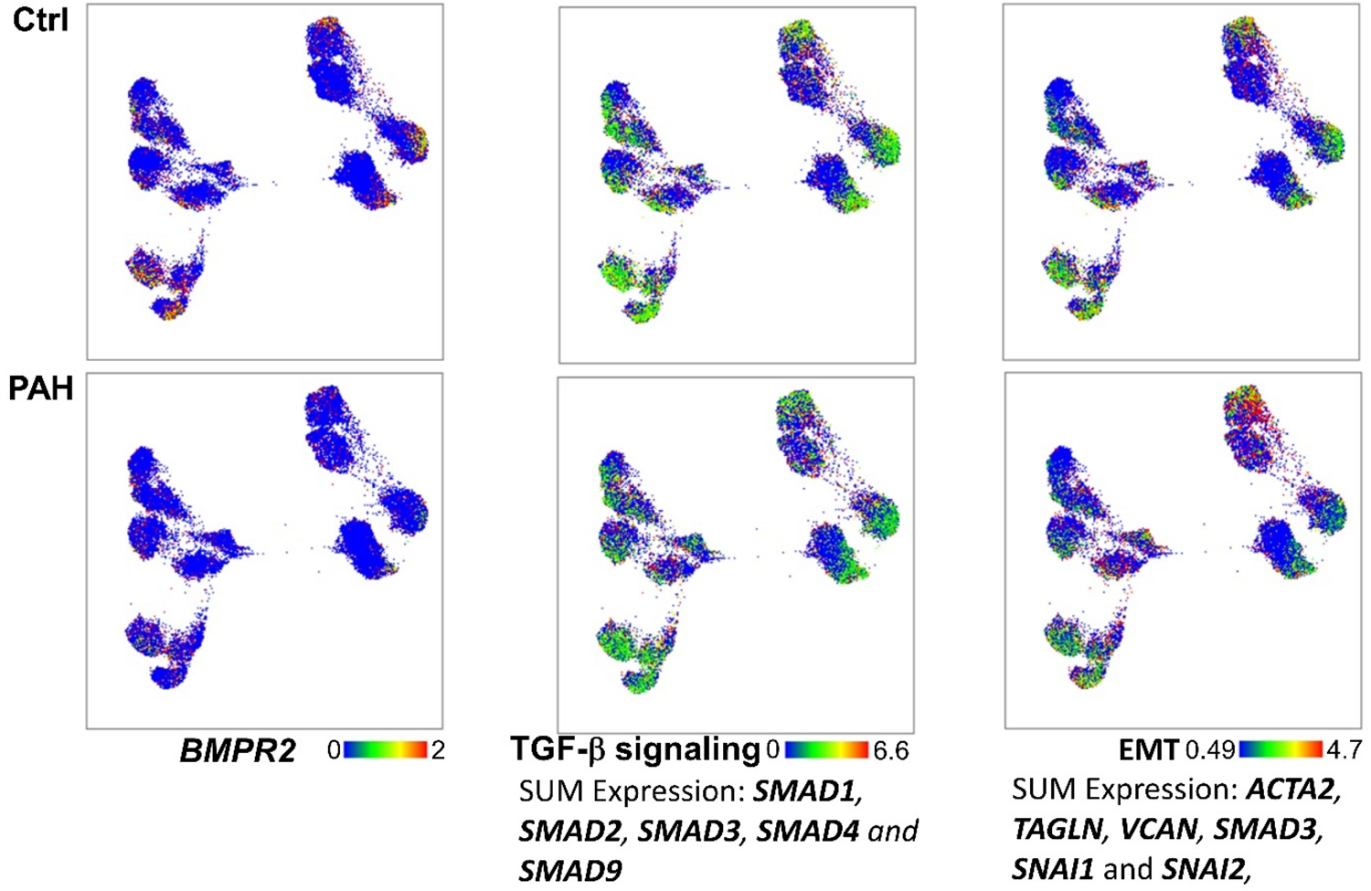
*BMPR2, TGF-β* signaling genes and Endothelial-to-Mesenchymal Transition markers. BMPR2 and sum expression of genes in TGF-β pathway and Endothelial-to-Mesenchymal Transition (EMT) is shown. Genes in bold were down-regulated in the total PAH PAEC population.

BMPR2, TGF-β signaling and Endothelial-to-Mesenchymal transition have been reported in PAH^25–27^. Several genes involved in these pathways were expressed by PAECs in both control and PAH across all clusters (Fig. 7). *BMPR2* and *SMAD* mRNA in TGF-β signaling pathways^28^ were down-regulated in PAH, consistent with the literature^29^. Similarly, genes in Endothelial-to-Mesenchymal transition pathways were also down-regulated among PAH PAECs^25–27^. This suggests that, at least in these PAH endothelial cells in culture, the expression pattern does not support evidence of endothelial-to-mesenchymal transition.

### Expression of genes in therapeutic pathways

**Figure 8:**
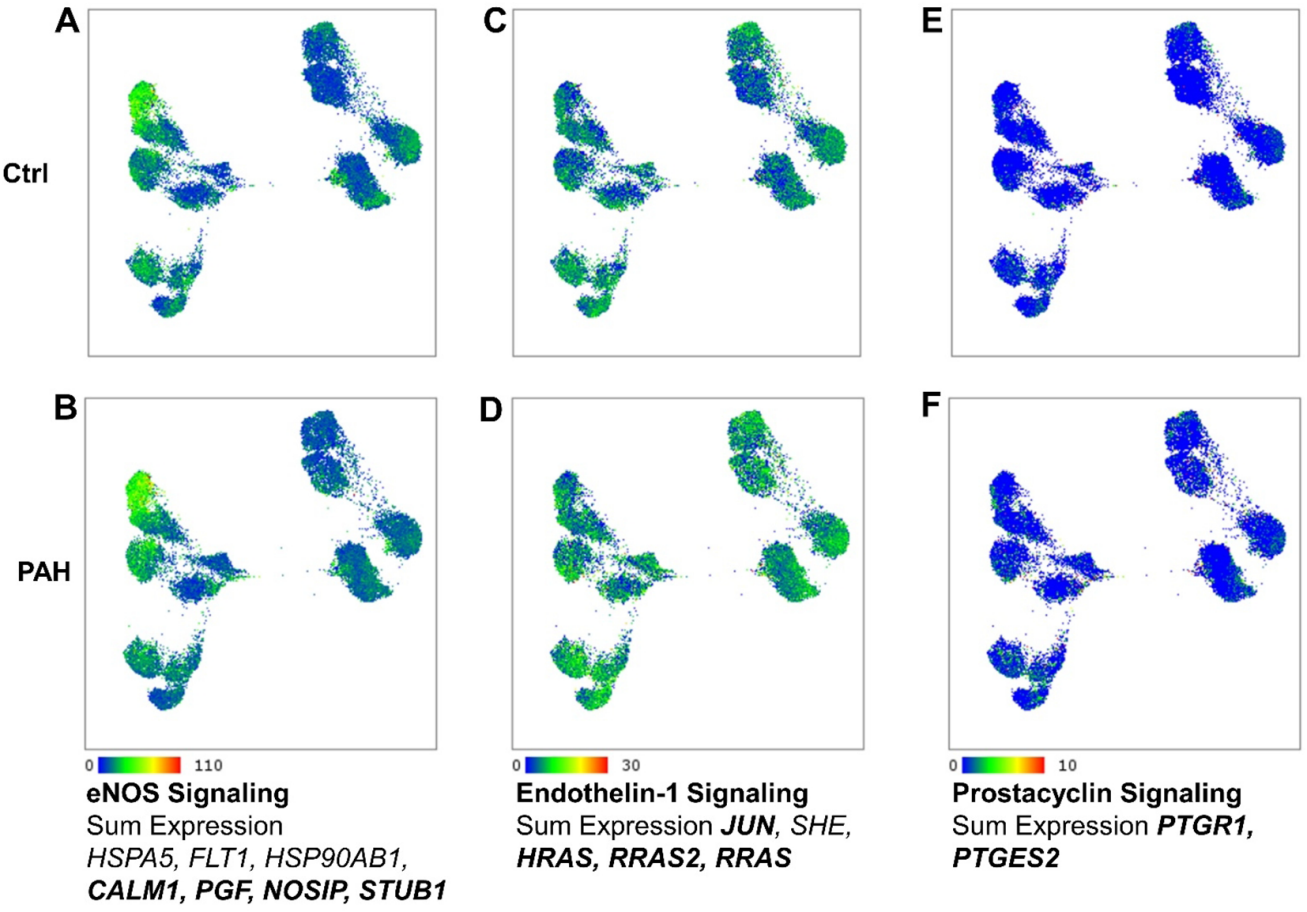
Genes in therapeutic pathways. Genes involved in eNOS (**A**,**B**), endothelin-1 (**C**,**D**) and prostacyclin (**E**,**F**) signaling pathways. Sum expression in each pathway is shown. Genes in bold were upregulated in the total PAH PAEC population.

Next, we focused on the three main therapeutic pathways in PAH; eNOS, endothelin-1 and prostacyclin^30,31^. It has been previously reported that endothelium-derived vasodilators, e.g., prostacyclin and nitric oxide (NO), are downregulated while endothelium-derived vasoconstrictors, e.g., endothelin-1, are upregulated in PAH^4,32–36^ Here, eNOS signaling related gene expression including calmodulin (*CALM*)*2/3*, NOS-interacting protein (*NOSIP*), placental growth factor (*PGF*), and E3 ubiquitin protein ligase (*STUB1*) were significantly increased, while heat shock protein family A member 5 (*HSPA5*), *HSP90AB1*, *HSP90B1*, and vascular endothelial growth factor receptor 1 (*FLT1*) were significantly reduced in PAH PAEC as compared with controls (Supplementary Tables S1 and S2) (Fig. 8). Gene expression of prostaglandin E synthase 2 (*PTGES2*) and prostaglandin reductase 1 (*PTGR1*) involved in prostacyclin pathway were significantly upregulated (Supplementary Tables S1 and S2) (Fig. 7). In addition, abnormality of endothelin-1 signaling related gene expression including transcription factor AP-1 (*JUN*), Ras family small GTP binding protein H-Ras (*HRAS*), RRAS, RRAS2, protein disulfide isomerase family A member 3 (PDIA3), and Src homology 2 domain containing E (*SHE*) was also found in PAH PAEC (Supplementary Tables S1 and S2) (Fig. 8). From all the genes involved in PAH therapeutic pathways, only *pgf* was differentially expressed across the clusters within controls and PAH. In both sources of PAEC, *pgf* was down-regulated in clusters 1, 2, and 3, and upregulated in clusters 6 and 7. In addition, *pgf* was also upregulated in PAH cluster 4.

## Discussion

Here we define the cellular heterogeneity among primary cultures of PAEC isolated from the main pulmonary artery and 1st–4th branches. Differential gene expression analysis on the whole population of control and PAH PAEC showed altered expression of more than 600 genes. Gene ontology enrichment analysis on these differentially expressed transcripts revealed that altered mitochondrial and angiogenesis processes characterized PAH PAEC. Oxidative phosphorylation and mitochondrial dysfunction are known to be dysregulated in PAH PAEC^5,14,15^. Ingenuity pathways analysis of the differentially expressed genes demonstrated dramatic up-regulation of EIF2 signaling, essential for protein synthesis, among PAH PAEC. EIF2 is essential for the initiation of protein synthesis via mediating the binding of tRNA to the ribosome. The role of EIF2 in PAH vascular remodeling and proliferation is well-established^11,14,16,17^. mTOR/eIF2α pathway is known to mediate hypoxic responses and vascular remodeling in pulmonary arterial vascular smooth muscle cells in a rat model of hypoxia-induced PAH^37^. The expression of IL-8 by PAH PAEC is another novel finding. IL-8 has an established role in PAH^38,39^ and is a key survival, proliferation and angiogenic factor^40^. Alterations in endothelium-derived vasodilator therapeutic pathways confirmed dysregulated vascular homeostasis in PAH^4,32–36^.

This report is the first single-cell omics analysis of a systemic large vessel vasculature in humans. Transcriptional profiling of single cells obtained from explanted idiopathic arterial hypertension and normal controls lungs showed that endothelial cells, in addition to pericyte/smooth muscle cells exhibited the highest number of differentially expressed gene sets, relative to other lung cells^41^. Even with a lenient false discovery rate of 10%, only 33 endothelial genes were upregulated in PAH in this study which analyzed a limited number of endothelial cells. Unfortunately, the exact fraction of endothelial cells among the average 3,688 total cells per patient was not reported. From the 33 unregulated endothelial cell genes, 3 genes, *apln, gas6 and arl6IP4,* also found in our cohort. Clustering within the endothelial cell subset was not reported in their study. Another study utilizing the mouse *Tabula Muris* consortium and Seurat Findcluster approach, identified 13 endothelial clusters in the lungs and other organs. The authors concluded that transcriptomic endothelial clustering may be independent of tissue of origin^42^.

We used a novel TriMap algorithm that provides a better preservation of the global data structure in the two-dimensional space^12^. Eight novel PAEC subsets were identified in both health control and PAH. Gene ontology analysis and highest overexpressed genes revealed key phenotypes of each cluster. PAH PAEC were characterized by three clusters with altered transcriptomic phenotypes. These included clusters 2, 4 and 8. While these clusters had a quiescent phenotype in controls, PAH cluster 2 had a proliferative and cluster 4 an angiogenic phenotype. Differential gene expression analysis between corresponding PAH and control clusters with altered profiles, revealed that recruitment of inflammatory cells, homeostasis of essential metals and coagulation regulation, essential processes in the pathogenesis of PAH^1,11,22^, are attributable to specific subsets of PAECs. In addition, the reduced expression of BMPR2 and altered expression of TGF-β signaling and Endothelial-to-Mesenchymal Transition marker among PAH PAECs indicated that PAH endothelial pathophysiology is well represented by PAECs. Overall, these data suggest that fundamental processes defining PAEC heterogeneity and identifying dysregulated endothelial cells in PAH originate from distinct subsets of cells. The findings of our study shed light on the transcriptomic diversity of PAEC. Identification of canonical dysregulated pathways demonstrate that PAH is characterized by an altered PAEC landscape in specific endothelial subsets. It should be kept in mind that these findings are in passaged cells. Further study is required to evaluate whether clusters vary among cells freshly harvested from PAH and control lungs. Nevertheless, the findings map transcriptomic heterogeneity among PAEC in health and vascular disease and serves as a comprehensive gene expression profile model to advance our understanding of endothelial dysfunction in PAH.

## Materials and methods

### Isolation of human PAEC

Human primary pulmonary arterial endothelial cells (PAEC) were collected either from unused explanted control donor lungs or explanted from PAH patients undergoing lung transplantation. The study was approved by the Cleveland Clinic Institutional Review Board, and informed written consent was obtained from all participants or tissue was under an Cleveland Clinic IRB exempt protocol. All methods were performed in accordance with the relevant guidelines and regulations.

PAEC from explanted PAH lungs or donor lungs not used for transplantation were harvested and cultured as previously described^43^. Donors for PAH lungs included 2 women and were of average age 33 ± 7 years old. They were transplanted at 16, 7 and 2 years after their diagnoses with PAH. All patients were on sildenafil, two of the patients were also on treprostinil, and one patient was also treated with prostacyclin and bosentan. Hemodynamic measurements were available and the mean pulmonary artery pressures were 53 ± 8.7 mmHg; cardiac output 3.5 ± 0.18 L/min; cardiac index 2 ± 0.19 L/min/m^2^ (mean ± SD, n = 3). Briefly, excess fat and connective tissue were removed from the main artery and rinsed (3 times) with Hanks’ Balanced Salt Solution (Invitrogen, Grand Island, NY). The arterial endothelial cells were removed by placing the arteries in 10–15 ml PBS containing 2 mg/ml type II collagenase (Worthington Biochem, Lakewood, NJ) at 37 °C and 5% CO_2_ with 90% humidity for 20 min. After incubation the arteries were placed in into MCDB-107 medium containing 14.8 g/l MCDB-105 (Sigma, St Louis, MO), 90 mg/l heparin (Sigma, St Louis, MO), 115 mg Endothelial Cell Growth Supplement (ECGS) (Sigma, St Louis, MO), 15.1 mg/l Glycine, 149 mg/l Potassium chloride (KCl), 10% Heat Inactivated Fetal Bovine Serum (FBS) (Lonza, Walkersville, MA), 1% Penicillin/Streptomycin/ Fungizone Solution (Invitrogen, Grand Island, NY) with pH 7.3. The endothelial cells were detach by a gentle shake and the residual arteries were removed before centrifugation at 330 g for 7 min at room temperature. The cell pellet was re-suspended in MCDB-107 medium and seeded on fibronection (1 mg/cm^2^) (CalBiochem, La Jolla, CA) coated cell culture plates. The cells were incubated at 37 °C, 5% CO_2_ with 90% humidity followed by media changes at 24 h and every 3 days until confluence. At passage 5 we tested the purity of the cell culture by immunophenotyping for CD31. All cells used in this paper were more than 96% CD31 positive. The passage of cells used in experiments was passage 6. Venous^44^ and alveolar capillary^45^ gene signatures were dimly present among the cells (Supplemental Fig. S4), showing that we sampled the same endothelial cell populations in PAH and controls.

### 10 × Single-cell RNA-Seq

PAEC suspensions with viabilities between 96 and 98% were used on 10 × platform for single-cell partitioning. The Chromium 3’ Solution V2 chemistry was used as instructed by the manufacturer.

### Library Prep and sequencing

Concentration and quality of double-stranded cDNA was assessed using a high-sensitivity DNA assay on an Agilent 2100 Bioanalyzer. Library preparation for sequencing on an Illumina platform was accomplished for each sample following the manufacturer’s protocol (CG000183 Rev A). Quality of library construction was again assessed using the Bioanalyzer. Samples were fluorometrically quantified with a Qubit (Thermo Fisher Scientific), pooled, and quantified on a Quantabio Q cycler using Quantabio SparQ Fast Library Quant kit. Sequence data was generated on a NovaSeq 6000 using parameters recommended by 10x (CG000183 Rev A).

### Cell ranger quality control and mapping

Raw sequencing data were processed with Cell Ranger (version 3.0.2), a proprietary package from 10 × Genomics. Reads QC and mapping were also performed within the Cell Ranger analysis pipeline. Reads were mapped to human reference genome, assembly GRCh38, using STAR aligner^46^. After obtaining mapped reads from the Cell Ranger pipeline, count matrices for the samples were produced using data in filtered_feature_bc_matrix directories and subsequently filtered to remove genes without any counts. Gene Ontology (GO) term enrichment analysis for Biological Processes was performed using a browser interface to MSigDB database v7.1^47–49^ A list of differentially expressed genes was provided and the list was compared to gene sets in the GO database using the hypergeometric test. Canonical pathway analysis of differentially expressed genes was done using QIAGEN Ingenuity Pathway Analysis (QIAGEN IPA, Qiagen, Redwood City, CA).

#### SeqGeq Bioinformatics

Data was analyzed in the SeqGeq bioinformatics platform, from the makers of FlowJo^50^. Techniques applied were done so following recommended bioinformatics protocols developed by the scientific community and honed by experts in single-cell data exploration at FlowJo, LLC. All differential expression analysis defined genes as up or down regulated if features showed Fold-Change >|2.0| and Bonferroni adjusted p-Values < 0.05 relative to comparator populations. Data files were concatenated together and normalized to Counts per 10 k reads, for uniform analysis of all matrices together. This ensured that uniform treatment was given to each dataset and made results more easily comparable between samples and conditions downstream.

## Supplementary Information

Supplementary Information.

## Data Availability

All raw mRNA-seq data files are available on NCBI's Gene Expression Omnibus (GEO), accessible using the following link https://www.ncbi.nlm.nih.gov/geo/query/acc.cgi?acc=GSE185479.
