# Author Correction: Single-cell transcriptomic profile of human pulmonary artery endothelial cells in health and pulmonary arterial hypertension

**DOI:** 10.1038/s41598-021-00563-5

**Published:** 2021-10-20

**Authors:** Kewal Asosingh, Suzy Comhair, Lori Mavrakis, Weiling Xu, Dean Horton, Ian Taylor, Svyatoslav Tkachenko, Bo Hu, Serpil Erzurum

**Affiliations:** 1 Departments of Inflammation and Immunity, Lerner Research Institute and Respiratory Institute, Cleveland Clinic, 9500 Euclid Ave, NB20, Cleveland, OH 44195 USA; 2 Departments of Quantitative Health Sciences, Lerner Research Institute and Respiratory Institute, Cleveland Clinic, Cleveland, OH USA; 3 Departments of Flow Cytometry Core, Lerner Research Institute and Respiratory Institute, Cleveland Clinic, Cleveland, OH USA; 4 Departments of Genomics Core, Lerner Research Institute and Respiratory Institute, Cleveland Clinic, Cleveland, OH USA; 5 BD Life Sciences: Informatics, Ashland, OR USA

Correction to: *Scientific Reports* 10.1038/s41598-021-94163-y, published online 19 July 2021

The original version of this Article omitted an URL to all of the raw single-cell omics data in Data availability section.

"All data associated with this study are available in the main text or the Supplementary materials."

now reads:

"All raw mRNA-seq data files are available on NCBI's Gene Expression Omnibus (GEO), accessible using the following link https://www.ncbi.nlm.nih.gov/geo/query/acc.cgi?acc=GSE185479."

The original Article has been corrected.

