# Supplementary material for: Single-cell transcriptomic profile of human pulmonary artery endothelial cells in health and pulmonary arterial hypertension
Source: Sci Rep. 2021 Jul 19;11:14714. doi: 10.1038/s41598-021-94163-y (PMC8289993; doi:10.1038/s41598-021-94163-y)
Supplement: Supplementary file 1 — Supplementary Information. [file 41598_2021_94163_MOESM1_ESM.pdf]

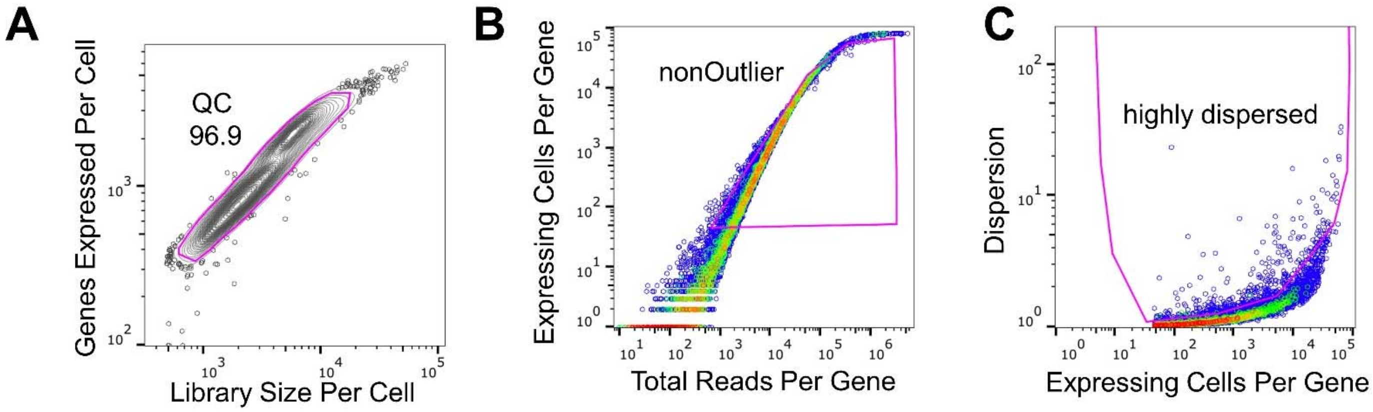

Supplemental Fig 1. Quality Control Filters for Cells and Genes. Quality cells were selected based on the library size per cell and the number of genes expressed per cell (A). Cell selected on this plot were then gated to exclude outliers on a total reads per gene vs. gene expression per cell (B). Highly dispersed genes were gated on an expression vs dispersion plot, with the understanding that parameters having higher dispersion give more discriminatory power and are inherantly less noisy for clustering and dimensionality reduction (C).

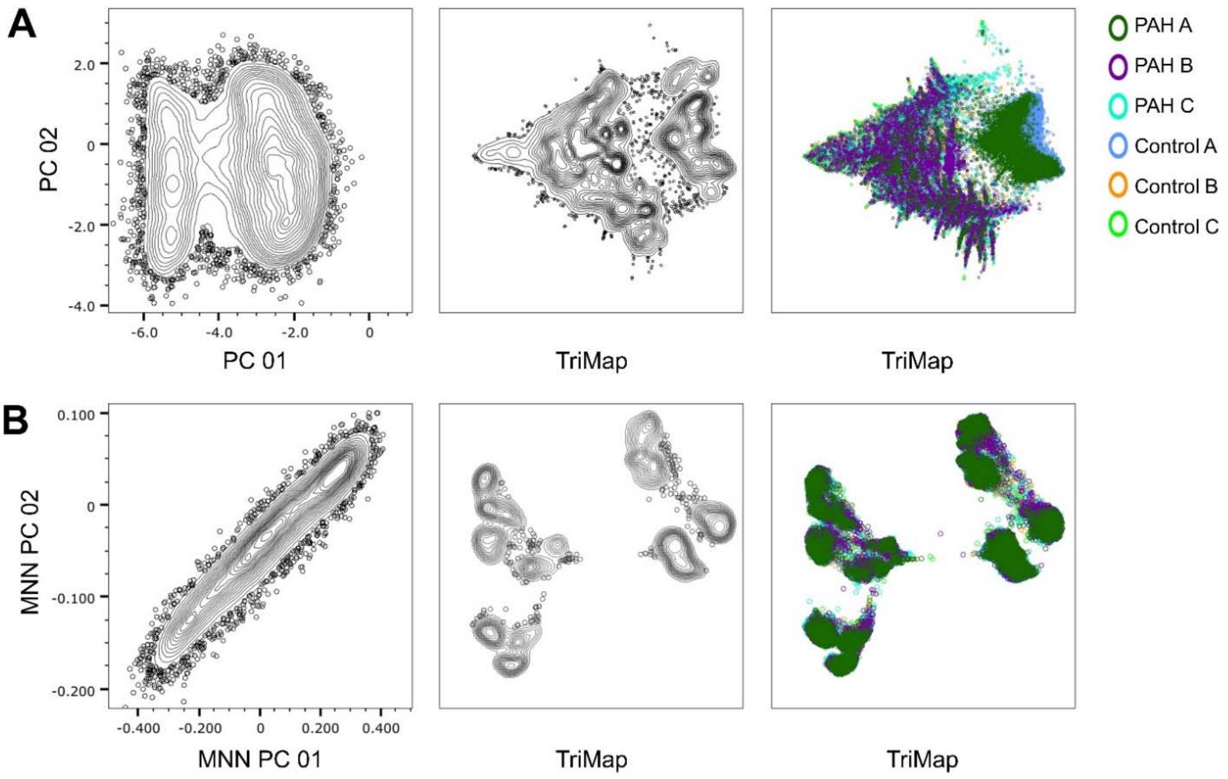

Supplemental Fig 2. Batch Effect Correction. Batch effect correction is a special type of principal component analysis (PCA) which tries to remove technical noise from different sample batches. PCA is a foundational method of dimensionality reduction, which serves to compress sparse data matrices and achieve a more robust clustering downstream. Panels A and B show two PC parameters and TriMap dimensionality reduction before (A) and after (B) batch effect correction. The comparison of initial dimensionality reduction and dimensionality reduction after mutual nearest neighbors correction (MNN) batch effect correction are shown.

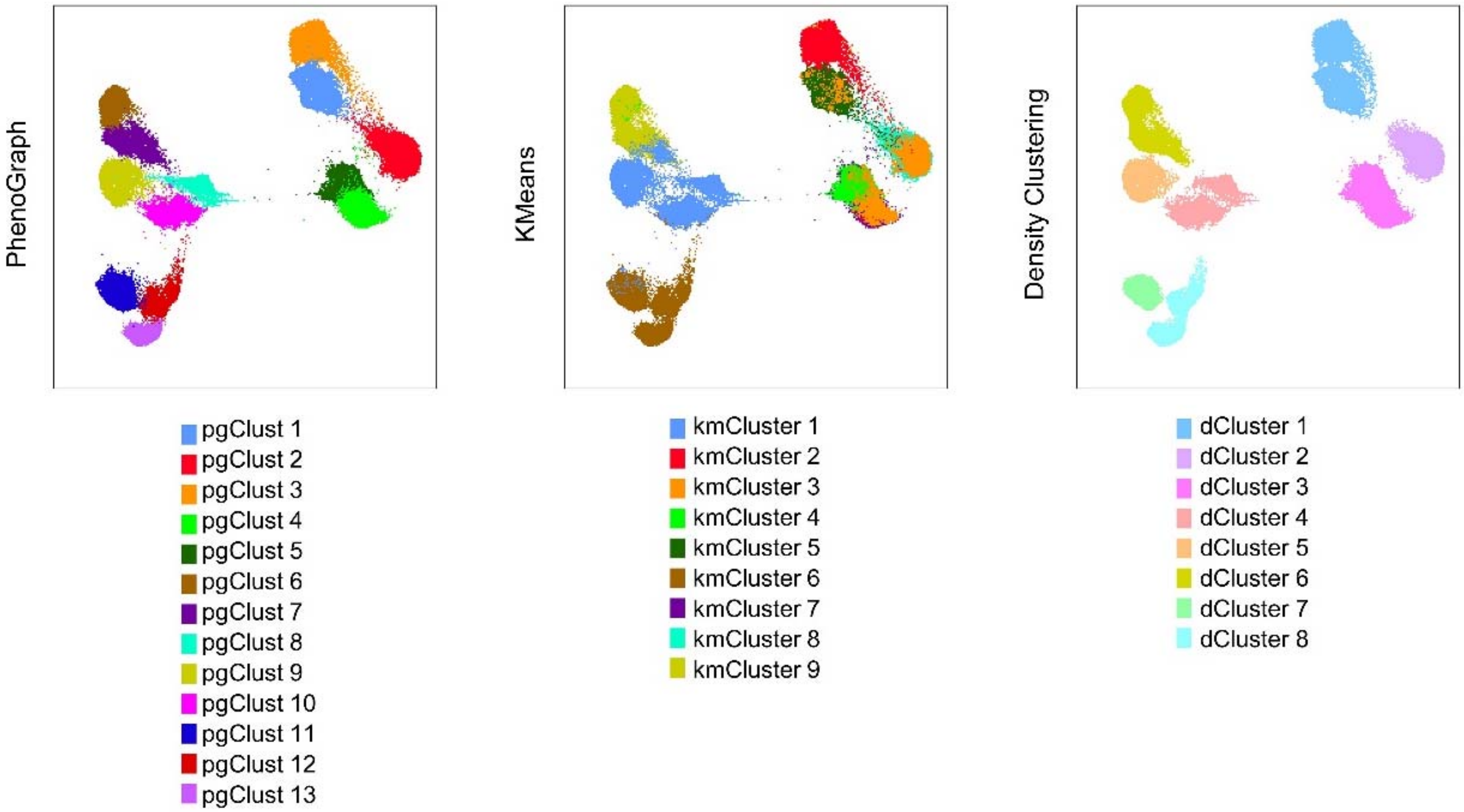

Supplemental Figure 3. Clustering Approaches. Three commonly used clustering algorithms, PhenoGraph, KMeans and Density Clustering were used to dissect the transcriptomic heterogeneity among PAEC. KMeans clustering utilizes vector quantization to minimize inter-cluster variance, while Phenograph is a K Nearest Neighbors based approach to unsupervised clustering of high dimensional data. Density clustering identifies populations in an unbiased fashion based on the density of islands across tSNE space, and seemed to perform best in this case.

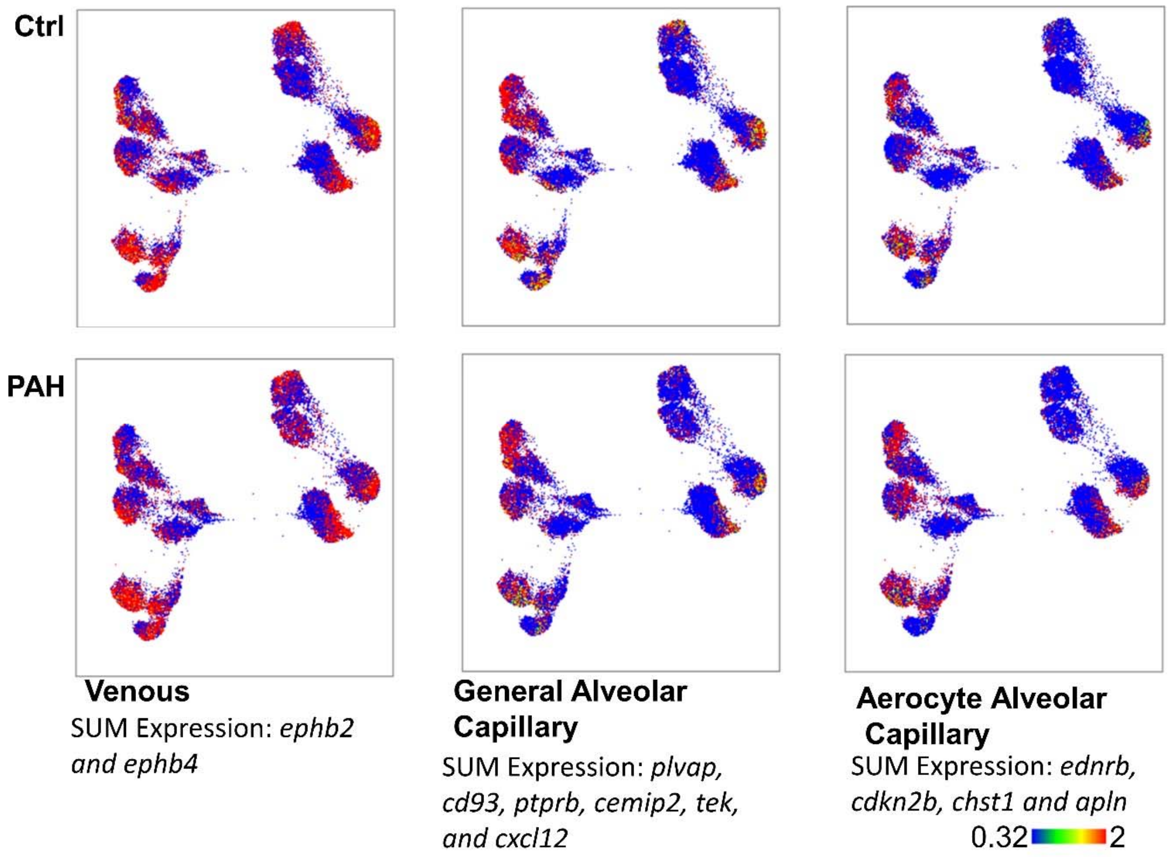

Supplemental Figure 4. Venous and capillary gene expression. The expression of venous and alveolar capillary signature genes was dim among PAECs (note the low scale of the heatmap bar).

Supplementary Table 1. Genes down-regulated in PAH PAECs

Genes	fold_change PAH vs Control	q-value	PAH	Control
RPL22	0.872871	1.35E-13	15.7256	18.1616
MFAP2	0.854364	1.35E-13	0.223413	0.431958
HSPG2	0.864525	1.35E-13	7.72826	9.09602
RPS8	0.858874	1.35E-13	57.4831	67.0927
PLPP3	0.870342	1.35E-13	0.309664	0.50477
RPL5	0.87151	1.35E-13	28.2463	32.5582
C1orf54	0.874081	1.35E-13	0.144002	0.308806
RPS27	0.693656	1.35E-13	18.9704	27.7901
SHE	0.881508	1.35E-13	0.133171	0.285491
DUSP23	0.81592	1.35E-13	0.623181	0.989387
SELP	0.88648	1.35E-13	0.0455046	0.179389
C1orf21	0.846825	1.35E-13	0.813502	1.14153
PXDN	0.849397	1.35E-13	2.03495	2.57307
PDIA6	0.888018	1.35E-13	1.32623	1.61958
DDX1	0.833484	1.35E-13	0.394832	0.673496
RHOB	0.826648	1.35E-13	0.94941	1.35821
OST4	0.883596	1.35E-13	6.50797	7.49706
EPAS1	0.893027	1.35E-13	0.854105	1.0762
EFEMP1	0.816041	1.35E-13	4.27132	5.45963
AAK1	0.818953	1.35E-13	0.536732	0.876459
MTHFD2	0.867783	1.35E-13	0.663014	0.916394
EIF5B	0.879815	1.35E-13	1.50285	1.84475
RPL31	0.774859	1.35E-13	19.8185	25.8674
IL1RL1	0.86495	1.35E-13	0.209677	0.398552
MIR4435-2HG	0.779143	1.35E-13	1.06785	1.654
DYNC1I2	0.848827	1.35E-13	0.77247	1.08814
TFPI	0.810257	1.35E-13	1.38546	1.94408
COL5A2	0.889097	1.35E-13	0.404281	0.579447
BMPR2	0.869976	1.35E-13	0.657497	0.905223
FN1	0.883409	1.35E-13	5.2848	6.11426
RPL37A	0.701226	1.35E-13	25.8547	37.2968
RPL32	0.890975	1.35E-13	55.369	62.2666
RPSA	0.891718	1.35E-13	40.6708	45.7309
HYAL2	0.863966	1.35E-13	0.757972	1.03477
SMIM4	0.881015	1.35E-13	0.313489	0.490881
MTRNR2L12	0.894663	1.35E-13	0.358103	0.518005
WWTR1	0.801808	1.35E-13	1.50687	2.12652
FNDC3B	0.8954	1.35E-13	0.807949	1.01915
ST6GAL1	0.868252	1.35E-13	0.220442	0.405631
MUC20-OT1	0.836454	1.35E-13	0.230384	0.470952
RPL35A	0.883551	1.35E-13	28.3133	32.1767
ATP5ME	0.695162	1.35E-13	1.92178	3.20303
LDB2	0.867555	1.35E-13	0.223912	0.410759
UCHL1	0.89159	4.02E-08	0.487221	0.668055
G3BP2	0.886606	1.35E-13	0.587987	0.791086

MMRN1	0.708608	1.35E-13	0.230263	0.736168
PPP3CA	0.89187	1.35E-13	0.516475	0.700332
RPL34	0.783527	1.35E-13	37.9074	48.6567
SNHG8	0.877854	1.35E-13	1.93243	2.34045
PCDH10	0.852692	1.35E-13	0.228317	0.440517
SLC7A11	0.885483	1.35E-13	1.81969	2.18435
MGARP	0.878235	1.35E-13	0.420138	0.617036
HHIP	0.808891	1.35E-13	4.42051	5.70116
MIR4458HG	0.87349	1.35E-13	0.117252	0.279067
RPL37	0.747892	1.35E-13	20.7937	28.1401
ITGA2	0.85098	1.35E-13	1.03435	1.3906
NSA2	0.823053	1.35E-13	0.788502	1.17301
TBCA	0.843003	1.35E-13	2.2281	2.82928
RPS23	0.83871	1.35E-13	54.5674	65.2535
COX7C	0.815978	1.35E-13	6.38551	8.05111
ERAP2	0.805657	1.35E-13	0.249147	0.550469
SPOCK1	0.877671	1.35E-13	2.54313	3.03697
CANX	0.89455	1.35E-13	1.96078	2.3098
SERPINB9	0.866696	1.35E-13	0.604513	0.8513
SOX4	0.818844	1.35E-13	2.36954	3.11499
HIST1H4C	0.873801	1.35E-13	1.56048	1.93028
ZSCAN16-AS1	0.889361	1.35E-13	0.139996	0.281815
IER3	0.844776	1.35E-13	1.41589	1.8598
HLA-C	0.887106	5.99E-12	1.24202	1.52735
RPS18	0.86544	1.35E-13	124.69	144.233
RPL10A	0.887389	1.35E-13	32.2441	36.4628
HSP90AB1	0.881586	1.35E-13	4.66686	5.42803
EEF1A1	0.87135	1.35E-13	102.446	117.72
GJA1	0.871668	1.35E-13	1.56546	1.94317
HEY2	0.864138	1.35E-13	0.204536	0.393917
SEC61G	0.873709	1.35E-13	2.13585	2.58913
PEG10	0.876648	1.35E-13	0.164597	0.328467
TRBC2	0.812948	1.35E-13	0.0530716	0.295373
PDIA4	0.889646	1.35E-13	0.520308	0.708892
GIMAP4	0.805776	1.35E-13	0.431061	0.776004
BMX	0.862512	1.35E-13	0.484736	0.72141
AP1S2	0.892307	1.35E-13	0.658187	0.858313
SAT1	0.835354	1.35E-13	3.23419	4.06874
SRPX	0.809636	1.35E-13	0.849944	1.28491
RPS4X	0.810049	1.35E-13	58.7754	72.7923
RPL36A	0.863415	1.35E-13	3.52613	4.24212
TCEAL9	0.861006	1.35E-13	0.780612	1.06806
RPL39	0.81059	1.35E-13	26.6296	33.0857
BGN	0.797931	1.35E-13	0.868111	1.34119
CTSB	0.85229	1.35E-13	2.93828	3.62082
MTUS1	0.868697	1.35E-13	1.14989	1.47484
DUSP4	0.891114	1.35E-13	0.576507	0.769142

RPS20		0.843437	1.35E-13	23.8685	28.4847
RPL7		0.887602	1.35E-13	36.6128	41.3757
FABP5		0.773164	1.35E-13	3.23924	4.48297
RUNX1T1		0.852193	1.35E-13	0.273622	0.494523
UQCRB		0.827749	1.35E-13	4.11247	5.17635
EIF3E		0.874291	1.35E-13	3.5697	4.22675
IL33		0.711958	1.35E-13	0.26024	0.770105
NFIB		0.878502	1.35E-13	1.69486	2.06757
TEK		0.896137	1.35E-13	0.2338	0.376798
DNAJA1		0.893412	1.35E-13	0.832059	1.05063
HSPA5		0.855289	1.35E-13	1.17419	1.54206
RPL7A		0.872202	1.35E-13	51.3199	58.986
RPLP2		0.872085	1.35E-13	43.9446	50.5369
RPL27A		0.845904	1.35E-13	47.8325	56.7281
SVIP		0.850752	1.35E-13	2.66949	3.31323
RCN1		0.781536	1.35E-13	1.65633	2.39886
TMEM258		0.844132	1.35E-13	2.09899	2.67121
FAU		0.89396	1.35E-13	15.2147	17.1381
MALAT1		0.847503	1.35E-13	103.686	122.523
PRCP		0.818198	1.35E-13	0.532366	0.872855
CTSC		0.840384	1.35E-13	0.714522	1.04016
RPS25		0.796431	1.35E-13	29.5968	37.4173
ITGB1		0.837156	1.35E-13	5.77936	7.09808
DEPP1		0.822272	1.32E-10	0.74143	1.11783
MMRN2		0.866469	1.35E-13	0.448761	0.672029
PTPRE		0.888609	1.35E-13	0.466342	0.650154
VWF		0.621591	1.35E-13	0.537588	1.47363
MGP		0.653788	1.35E-13	0.27102	0.944086
LDHB		0.879314	1.35E-13	3.68169	4.32426
EIF4B		0.889181	1.35E-13	2.11651	2.50493
ATP5MC2		0.863029	1.35E-13	5.50721	6.53996
RPS26		0.699333	1.35E-13	8.54142	12.6436
NAP1L1		0.827655	1.35E-13	6.80489	8.43012
DUSP6		0.850897	1.35E-13	0.810298	1.12752
NUDT4		0.784973	1.35E-13	1.6025	2.31541
METAP2		0.880239	1.35E-13	0.77536	1.01691
SNRPF		0.892116	1.35E-13	1.37766	1.66519
ELK3		0.845658	1.35E-13	0.449361	0.713884
HSP90B1		0.843657	1.35E-13	1.50311	1.96698
CKAP4		0.852259	1.35E-13	1.5883	2.03699
RPL21		0.857825	1.35E-13	40.2509	47.0877
POLR1D		0.87223	1.35E-13	1.86266	2.28201
FLT1		0.875189	1.35E-13	0.263039	0.443161
KCTD12		0.862888	1.35E-13	1.48759	1.88287
MBNL2		0.895379	1.35E-13	0.486528	0.660221
EFNB2		0.853149	1.35E-13	0.331956	0.561224
PNP		0.89312	1.35E-13	0.724791	0.931197

CLEC14A		0.870167	1.35E-13	0.811036	1.08125
RPS29		0.626913	1.35E-13	12.9894	21.3147
RHOJ		0.864172	1.35E-13	0.987869	1.30032
SLIRP		0.887162	1.35E-13	1.48375	1.79965
NDUFB1		0.815948	1.35E-13	0.924003	1.358
THBS1		0.808983	1.35E-13	2.94087	3.87139
PDIA3		0.875088	1.35E-13	0.842615	1.10564
EIF3J		0.85209	1.35E-13	0.718401	1.01669
RPS17		0.893602	1.35E-13	2.98719	3.46193
RPS15A		0.86468	1.35E-13	42.4375	49.2353
CDH5		0.882092	1.35E-13	2.06963	2.47994
CFDP1		0.895711	1.35E-13	0.809443	1.02012
WSB1		0.875979	1.35E-13	0.897999	1.16672
RPL23A		0.839044	1.35E-13	36.7096	43.9435
RPL23		0.777419	1.35E-13	17.7624	23.1342
IGFBP4		0.803351	1.35E-13	1.01004	1.50206
RPL27		0.87076	1.35E-13	22.0366	25.4558
PECAM1		0.856194	1.35E-13	3.21111	3.91841
ABCA8		0.840621	1.35E-13	0.186415	0.411355
RPL38		0.707624	1.35E-13	7.55639	11.0917
COLEC12		0.885876	1.35E-13	0.122343	0.266931
TCF4		0.833884	1.35E-13	2.31196	2.97173
BMP2		0.839268	1.35E-13	0.504628	0.792787
CST1		0.714628	1.35E-13	1.16776	2.03341
EIF2S2		0.820353	1.35E-13	2.78149	3.60958
ROMO1		0.864029	1.35E-13	1.05508	1.37849
TGM2		0.864832	1.35E-13	3.27004	3.93743
PKIG		0.865684	1.35E-13	1.7273	2.15045
ZFAS1		0.858081	1.35E-13	5.08894	6.096
ATP5F1E		0.857847	1.35E-13	10.7594	12.708
RPS21		0.832347	1.35E-13	11.2392	13.7045
SOX18		0.869507	1.35E-13	0.282171	0.474596
RPL36		0.863595	1.35E-13	24.7203	28.7828
RPS28		0.772947	1.35E-13	26.1351	34.1061
CALR		0.835414	1.35E-13	2.87758	3.64151
RPL13A		0.872972	1.35E-13	100.381	115.133
RPS11		0.852383	1.35E-13	17.1799	20.3283
NDUFA3		0.80449	1.35E-13	0.625585	1.02064
FAM118A		0.805211	1.35E-13	0.0882703	0.351535
PIM3		0.890743	3.13E-05	0.603692	0.800398
CCT8		0.858494	1.35E-13	0.872201	1.1808
COL18A1		0.881118	1.35E-13	1.37595	1.69652
MT-ND3		0.589073	1.35E-13	6.75671	12.1676
MT-ND4L		0.799362	1.35E-13	0.499514	0.875887
MT-ND6		0.857944	1.35E-13	0.597674	0.862212

Supplementary Table 2. Genes up-regulated in PAH PAECs

Genes	fold_change PAH vs Control	q-value	PAH	Control
HES4	1.29243	1.35E-13	0.740437	0.346643
ISG15	1.21225	1.35E-13	1.33656	0.927461
AURKAIP1	1.18109	1.35E-13	3.81426	3.07613
SSU72	1.11094	1.35E-13	2.32711	1.99485
FAAP20	1.10945	1.35E-13	1.47847	1.23396
RER1	1.16497	1.35E-13	1.18066	0.871856
SRM	1.17946	1.35E-13	3.38276	2.7159
EFHD2	1.17879	1.35E-13	1.53394	1.14961
NBL1	1.13282	1.35E-13	0.749326	0.544227
LUZP1	1.11252	1.35E-13	1.37638	1.13603
SH3BGRL3	1.29587	1.35E-13	30.1081	23.0057
SMIM12	1.105	1.35E-13	0.872364	0.694449
TRAPPC3	1.13805	1.35E-13	1.18765	0.922283
EVA1B	1.222	1.35E-13	1.95512	1.41828
LSM10	1.11112	1.35E-13	1.41854	1.17667
NDUFS5	1.14931	1.35E-13	4.23414	3.55415
EBNA1BP2	1.13109	1.35E-13	1.04738	0.810091
HYI	1.09766	1.35E-13	1.02253	0.842577
ATP6V0B	1.2017	1.35E-13	1.51163	1.09006
JUN	1.11307	1.35E-13	2.0557	1.7453
JAK1	1.10895	1.35E-13	2.48041	2.13848
NEXN	1.09616	3.68E-05	0.36436	0.24467
GCLM	1.12241	1.35E-13	0.849933	0.648184
GSTM3	1.13629	1.35E-13	0.3651	0.201367
RHOC	1.3144	1.35E-13	5.88624	4.23907
S100A11	1.31852	1.35E-13	7.1221	5.16002
S100A16	1.20816	1.35E-13	14.0285	11.4391
JTB	1.15819	1.35E-13	3.18948	2.61725
PMVK	1.09754	1.35E-13	0.65672	0.50949
GPATCH4	1.1011	1.35E-13	0.618723	0.470096
PEA15	1.15679	1.35E-13	1.52645	1.18402
RGS4	1.22428	1.35E-13	1.3293	0.90258
PHLDA3	1.11717	1.35E-13	0.803751	0.614576
CSRP1	1.26227	1.35E-13	2.00886	1.38368
NENF	1.14145	1.35E-13	1.54261	1.22752
MRPL55	1.12026	1.35E-13	1.04434	0.824879
GUK1	1.2535	1.35E-13	9.57792	7.43867
FKBP1B	1.1163	1.35E-13	0.585648	0.420455
PPM1G	1.11134	1.35E-13	1.39975	1.15932
CALM2	1.12282	1.35E-13	4.192	3.62409
RTN4	1.10891	1.35E-13	4.89555	4.31654
PRADC1	1.10026	1.35E-13	0.478661	0.343916
VAMP8	1.21246	1.35E-13	0.538329	0.268762
FHL2	1.2249	1.35E-13	2.60304	1.9415
GYPC	1.28891	1.35E-13	0.609322	0.24859

MZT2B	1.19206	1.35E-13	7.85107	6.42499
LYPD1	1.1329	1.35E-13	0.837425	0.621883
OLA1	1.09622	1.35E-13	0.92598	0.756926
SPATS2L	1.108	1.35E-13	0.892458	0.707994
ARPC2	1.22757	1.35E-13	8.47406	6.71776
LRRFIP1	1.09937	1.35E-13	1.56547	1.33359
TWIST2	1.09916	3.29E-11	0.358178	0.235656
DTYMK	1.13699	1.35E-13	1.21667	0.949602
LRRFIP2	1.10039	1.35E-13	1.12686	0.932815
CDCP1	1.18071	1.35E-13	0.538254	0.302819
TMEM158	1.21168	1.35E-13	0.531913	0.264283
NDUFAF3	1.11367	1.35E-13	1.76182	1.47993
RPL29	1.09658	1.35E-13	50.2077	45.6978
DCBLD2	1.1498	1.35E-13	0.799142	0.564745
MGLL	1.27064	1.35E-13	1.90596	1.28699
H1FX	1.15188	1.35E-13	0.664882	0.445362
CDV3	1.10036	1.35E-13	1.69398	1.44827
SSR3	1.13585	1.35E-13	2.74748	2.29928
CLDN11	1.11422	1.35E-13	1.49942	1.2432
FAM43A	1.11703	1.35E-13	1.96053	1.65037
DLG1	1.14146	1.35E-13	0.692229	0.482518
MXD4	1.11158	1.35E-13	1.39853	1.15776
MFSD10	1.09666	2.99E-11	0.493809	0.362148
OCIAD2	1.23985	1.35E-13	3.61922	2.72563
HOPX	1.33997	1.35E-13	1.06914	0.544177
IGFBP7	1.1671	1.35E-13	4.34969	3.58375
CXCL6	1.24683	8.92E-10	0.450935	0.163697
CXCL1	1.13771	9.06E-05	1.84807	1.50335
11-Sep	1.11337	1.35E-13	1.662	1.39094
PYURF	1.10231	1.35E-13	1.99417	1.71628
HMGB2	1.10596	1.16E-06	0.900505	0.718417
NDUFS6	1.13465	1.35E-13	3.23018	2.72818
BASP1	1.17004	1.35E-13	2.47316	1.96842
FST	1.27869	1.35E-13	0.883844	0.473257
ESM1	1.24786	1.35E-13	1.73875	1.19476
VCAN	1.10497	0.000417	0.202857	0.0885918
NR2F1-AS1	1.0968	1.35E-13	0.87405	0.70866
CAST	1.11565	1.35E-13	1.91915	1.61656
HINT1	1.12934	1.35E-13	15.9927	14.0466
PDLIM4	1.14627	1.35E-13	1.28678	0.994972
ECSCR	1.12412	1.35E-13	5.37881	4.67451
UBE2D2	1.10694	1.35E-13	2.28245	1.96535
CYSTM1	1.10169	1.35E-13	1.31237	1.09893
HBEGF	1.09609	1.49E-08	0.341847	0.224209
CCDC69	1.10126	4.49E-11	0.34498	0.221312
PTTG1	1.13147	1.35E-13	1.2692	1.00554
ATP6V0E1	1.31272	1.35E-13	5.01127	3.57925

CLTB		1.24361	1.35E-13	2.71423	1.98664
PRELID1		1.21812	1.35E-13	7.32805	5.83682
PDLIM7		1.12167	1.35E-13	1.56936	1.29066
MGAT4B		1.13744	1.35E-13	1.65097	1.33066
TMEM14B		1.1645	1.35E-13	1.66512	1.28864
ADTRP		1.12552	1.35E-13	0.615471	0.43531
DEK		1.10402	1.35E-13	1.89355	1.62091
FLOT1		1.15476	1.35E-13	0.752781	0.517872
CLIC1		1.20516	1.35E-13	3.85994	3.03263
CUTA		1.1691	1.35E-13	1.80174	1.39649
RPS10		1.21359	1.35E-13	8.32932	6.68735
SRSF3		1.09783	1.35E-13	2.70915	2.37862
MTCH1		1.1352	1.35E-13	1.88546	1.54181
CCND3		1.10215	8.65E-10	0.325096	0.202287
MEA1		1.13138	1.35E-13	1.08212	0.840334
SNX3		1.13226	1.35E-13	3.323	2.81804
SGK1		1.11123	1.35E-13	0.486508	0.337718
SF3B5		1.10162	1.35E-13	2.43562	2.11871
RAB32		1.11394	1.35E-13	2.05296	1.7407
AKAP12		1.16519	1.35E-13	1.71815	1.3328
C7orf50		1.10408	1.35E-13	1.3133	1.09523
ZFAND2A		1.11106	1.35E-13	0.293421	0.164132
RAC1		1.25737	1.35E-13	8.26339	6.36726
SUGCT		1.11642	0.000139	0.176223	0.0535707
POLD2		1.09912	1.35E-13	0.975303	0.797169
PPIA		1.14951	1.35E-13	10.5412	9.04013
CCM2		1.10713	1.35E-13	0.778032	0.605987
SBDS		1.13118	1.35E-13	0.69913	0.502085
GNG11		1.19141	1.35E-13	12.3626	10.2158
BRI3		1.16821	1.35E-13	11.1186	9.37366
PDAP1		1.10353	1.35E-13	1.9579	1.68039
LAMTOR4		1.11256	1.35E-13	2.84785	2.45856
POP7		1.1732	1.35E-13	1.07227	0.766343
FIS1		1.12847	1.35E-13	2.88234	2.44037
POLR2J		1.11256	1.35E-13	2.39234	2.04913
CAV2		1.11691	1.35E-13	2.47718	2.11321
ATP6V1F		1.19288	1.35E-13	4.11334	3.28655
CALD1		1.25392	1.35E-13	5.22888	3.96754
RHEB		1.17157	1.35E-13	1.78972	1.38118
TMSB4X		1.16127	1.35E-13	140.781	121.091
PRDX4		1.11283	1.35E-13	1.20005	0.976995
TIMP1		1.27305	1.35E-13	2.93158	2.08831
PLP2		1.1697	1.35E-13	1.77722	1.37429
APLN		1.21457	1.35E-13	1.61542	1.15337
FHL1		1.11222	1.35E-13	0.607866	0.445634
BCAP31		1.11403	1.35E-13	1.90858	1.61088
SSR4		1.10966	1.35E-13	3.21864	2.80174

NAA10	1.1503	1.35E-13	2.39325	1.94989
IRAK1	1.10097	1.35E-13	1.08137	0.890485
FLNA	1.26266	1.35E-13	3.85113	2.842
FAM50A	1.16217	1.35E-13	1.00173	0.722406
G6PD	1.12485	1.35E-13	0.67707	0.490929
DLC1	1.10754	1.35E-13	0.847468	0.66809
STC1	1.11872	2.99E-12	0.434069	0.281883
PLAT	1.2272	1.35E-13	1.18641	0.781626
SMIM19	1.10296	7.78E-10	0.431388	0.297775
MTDH	1.17517	1.35E-13	3.84935	3.12651
NDUFB9	1.11798	1.35E-13	2.43301	2.07072
LY6E	1.18831	1.35E-13	1.41578	1.03295
GSDMD	1.16103	1.35E-13	0.442647	0.242563
TSTA3	1.11223	1.35E-13	0.619337	0.45594
PLEC	1.24687	1.35E-13	1.74364	1.20042
MAF1	1.12062	1.35E-13	1.04537	0.825204
PLGRKT	1.11288	3.53E-08	0.281261	0.151303
CDKN2A	1.11281	1.35E-13	1.14136	0.924286
BAG1	1.10448	1.35E-13	0.591031	0.440525
PRSS3	1.28509	1.35E-13	4.58103	3.34289
STOML2	1.10912	1.35E-13	2.71638	2.35074
TPM2	1.10347	1.35E-13	2.27787	1.97051
MSMP	1.1143	1.35E-13	0.478169	0.326551
HINT2	1.10108	1.35E-13	0.701014	0.544856
CLTA	1.11154	1.35E-13	4.26544	3.73705
CKS2	1.15113	1.35E-13	1.4864	1.15998
NINJ1	1.15584	1.35E-13	0.816296	0.571413
NANS	1.12139	1.35E-13	0.554982	0.386651
GABBR2	1.18661	1.35E-13	0.706783	0.438367
TXN	1.12765	1.35E-13	7.16761	6.24302
PTGR1	1.12199	1.35E-13	0.452126	0.294242
ATP6V1G1	1.10327	1.35E-13	2.51361	2.18474
ARPC5L	1.10967	1.35E-13	1.66601	1.40252
PTRH1	1.12461	1.35E-13	0.600475	0.423141
PTGES2	1.10781	1.35E-13	0.620794	0.463064
C9orf16	1.14477	1.35E-13	3.09762	2.57944
ZDHHC12	1.10772	1.35E-13	0.81588	0.639296
NTMT1	1.13041	1.35E-13	0.70434	0.507725
SURF1	1.15066	1.35E-13	0.556375	0.352596
EGFL7	1.26744	1.35E-13	6.69272	5.06951
AGPAT2	1.14232	1.35E-13	0.638393	0.434274
TMEM141	1.12924	1.35E-13	1.30589	1.04199
RABL6	1.13819	1.35E-13	1.224	0.953984
PHPT1	1.10122	1.35E-13	3.56785	3.148
EDF1	1.20192	1.35E-13	4.69685	3.73978
FBXW5	1.18597	1.35E-13	0.920062	0.618977
PAXX	1.14044	1.35E-13	0.798129	0.576704

NPDC1		1.11671	1.35E-13	2.53576	2.16622
DPP7		1.11839	1.35E-13	0.900072	0.69894
TUBB4B		1.17928	1.35E-13	2.51035	1.97668
MRPL41		1.19483	1.35E-13	2.97868	2.32991
IFITM2		1.10759	1.35E-13	2.76423	2.39859
IFITM3		1.18895	1.35E-13	3.79928	3.03658
SIGIRR		1.12487	1.35E-13	1.10602	0.872226
RNH1		1.10098	1.35E-13	1.24674	1.04066
HRAS		1.14234	1.35E-13	1.47013	1.16235
TALDO1		1.24249	1.35E-13	2.16218	1.54503
CD151		1.26233	1.35E-13	2.63723	1.88136
TSPAN4		1.22479	1.35E-13	0.828928	0.493255
CD81		1.23145	1.35E-13	3.03857	2.27951
PHLDA2		1.17159	1.35E-13	1.88749	1.4646
RHOG		1.11643	1.35E-13	0.749225	0.566807
CAVIN3		1.11243	1.35E-13	1.9468	1.64897
TAF10		1.10803	1.35E-13	1.54347	1.2955
RRAS2		1.1045	1.35E-13	0.591091	0.440549
CD44		1.15639	1.35E-13	2.86363	2.34111
TIMM10		1.14045	1.35E-13	0.713374	0.502369
FTH1		1.38455	1.35E-13	224.885	162.146
UQCC3		1.15754	1.35E-13	0.946548	0.681627
PPP1R14B		1.10632	1.35E-13	7.36982	6.56545
BAD		1.14041	1.35E-13	0.945061	0.705582
PRDX5		1.19542	1.35E-13	4.29906	3.43282
ARL2		1.12144	1.35E-13	1.57392	1.29519
CDC42EP2		1.13803	1.35E-13	0.655335	0.454564
RNASEH2C		1.1099	1.35E-13	1.74448	1.47272
CFL1		1.20521	1.35E-13	12.8575	10.498
FIBP		1.12033	1.35E-13	1.84828	1.54236
CCDC85B		1.42147	1.35E-13	6.28969	4.12826
FOSL1		1.13716	1.35E-13	1.06801	0.818571
DRAP1		1.10856	1.35E-13	2.34283	2.01547
YIF1A		1.11962	1.35E-13	1.16329	0.932165
PPP1CA		1.13118	1.35E-13	1.37412	1.0988
NDUFS8		1.15121	1.35E-13	2.32708	1.89008
CCND1		1.59857	1.35E-13	6.19176	3.49888
LAMTOR1		1.15575	1.35E-13	2.87013	2.34858
NDUFC2		1.15318	1.35E-13	2.12026	1.70579
MMP10		1.26973	1.35E-13	0.404453	0.106102
CARD16		1.1683	1.35E-13	0.627807	0.393308
TAGLN		1.15558	1.56E-06	0.341996	0.161314
NRGN		1.10997	1.35E-13	0.786377	0.609399
FRMD4A		1.11835	1.35E-13	1.17413	0.944043
CREM		1.12167	1.35E-13	0.688313	0.505179
ARHGAP22		1.12139	1.35E-13	0.602136	0.428706
DKK1		1.16692	1.35E-13	1.12478	0.820847

PCBD1		1.21447	1.35E-13	1.97334	1.44825
MRPS16		1.1094	1.35E-13	1.79323	1.51779
PLAU		1.19324	1.35E-13	0.570637	0.316284
ANXA11		1.10471	1.35E-13	1.14149	0.938515
SNCG		1.14357	1.35E-13	0.925596	0.683848
ADIRF		1.21613	1.35E-13	3.99225	3.10503
UBTD1		1.11135	1.35E-13	0.581623	0.423157
HPS1		1.14901	1.35E-13	0.779502	0.54873
NDUFB8		1.09762	1.35E-13	2.45223	2.14519
MRPL43		1.12655	1.35E-13	1.19927	0.952206
ACTR1A		1.1125	1.35E-13	0.828034	0.643177
GSTO1		1.10549	1.35E-13	4.41645	3.89959
UROS		1.12165	1.35E-13	1.09518	0.867936
MKI67		1.11583	4.50E-06	0.733862	0.553873
ZNF511		1.11206	1.35E-13	0.573105	0.414585
CCND2		1.23158	1.35E-13	0.673935	0.359178
CD9		1.13659	1.35E-13	5.86016	5.03572
MRPL51		1.10312	1.35E-13	3.07897	2.69768
GAPDH		1.19814	1.35E-13	51.3283	42.6748
MLF2		1.13446	1.35E-13	1.2643	0.995938
PTMS		1.28616	1.35E-13	4.92749	3.60867
TPI1		1.11604	1.35E-13	4.97387	4.35273
SMIM10L1		1.09715	1.35E-13	0.616386	0.473262
SMAGP		1.10019	1.35E-13	0.637284	0.488181
GALNT6		1.11184	1.35E-13	0.611174	0.449111
ATG101		1.13479	1.35E-13	0.548328	0.364419
KRT7		1.21446	1.35E-13	3.11236	2.38617
IGFBP6		1.18875	1.35E-13	0.522777	0.280987
PRR13		1.10281	1.35E-13	1.46654	1.23659
BLOC1S1		1.12659	1.35E-13	1.6136	1.31992
CD63		1.1433	1.35E-13	6.78937	5.81303
MYL6		1.22585	1.35E-13	17.4974	14.0894
BTG1		1.11376	1.35E-13	2.03943	1.72899
TXNRD1		1.12074	1.35E-13	2.63868	2.24668
ISCU		1.1296	1.35E-13	1.56044	1.26668
SRSF9		1.09836	1.35E-13	2.34438	2.04487
DYNLL1		1.21411	1.35E-13	9.60978	7.73873
ORAI1		1.12494	1.35E-13	0.61067	0.431783
RHOF		1.13562	1.35E-13	0.513706	0.332933
ARL6IP4		1.1282	1.35E-13	5.29708	4.58151
RGCC		1.28579	1.35E-13	1.12746	0.654586
DNAJC15		1.12366	1.35E-13	0.884431	0.677046
UCHL3		1.09775	5.99E-12	0.509568	0.375144
LMO7		1.33483	1.35E-13	0.577282	0.181633
GAS6		1.16944	1.35E-13	0.682036	0.438327
UPF3A		1.11471	1.35E-13	0.665183	0.49383
RNASE1		1.17123	1.35E-13	2.12244	1.66595

FRMD6	1.12231	1.35E-13	0.850757	0.649053
PGF	1.11023	1.35E-13	4.97411	4.38099
IFI27	1.62851	1.35E-13	8.73629	4.97865
EIF5	1.11348	1.35E-13	2.09443	1.77906
SIVA1	1.12492	1.35E-13	2.56346	2.16775
AHNAK2	1.10946	1.35E-13	1.25399	1.0316
CRIP2	1.19721	1.35E-13	3.53516	2.78812
SNRPN	1.18683	1.35E-13	1.03959	0.71851
EMC4	1.09562	1.35E-13	0.977109	0.804549
SPG21	1.10148	1.35E-13	0.90744	0.731713
UACA	1.15392	1.35E-13	0.835638	0.590791
PKM	1.151	1.35E-13	4.83888	4.07287
ISG20	1.11199	1.35E-13	0.660992	0.493709
CIB1	1.1026	1.35E-13	1.62796	1.38342
SNRNP25	1.10068	1.35E-13	0.882861	0.710628
MRPL28	1.11051	1.35E-13	0.708761	0.538711
METTL26	1.17939	1.35E-13	2.34737	1.83821
MCRIP2	1.09579	1.35E-13	0.767124	0.612655
STUB1	1.24962	1.35E-13	2.58628	1.86989
METRN	1.16812	1.35E-13	1.25854	0.93348
FAM173A	1.15773	1.35E-13	0.728625	0.493118
TSR3	1.14357	1.35E-13	1.10692	0.842398
MRPS34	1.1141	1.35E-13	1.30159	1.06588
NUBP2	1.09843	1.35E-13	0.675644	0.525486
NDUFB10	1.10579	1.35E-13	2.95651	2.578
SLC9A3R2	1.15465	1.35E-13	1.11097	0.828233
PGP	1.13316	1.35E-13	1.08186	0.837212
TNFRSF12A	1.18336	1.35E-13	1.29264	0.937396
CARHSP1	1.10632	1.35E-13	1.40517	1.17402
MVP	1.19392	1.35E-13	1.13431	0.787653
TGFB1I1	1.11225	1.35E-13	0.988826	0.788103
MT1E	1.40476	1.35E-13	0.944762	0.384412
TMEM208	1.10615	1.35E-13	0.624348	0.46847
NQO1	1.15482	1.35E-13	1.94735	1.55221
COTL1	1.18131	1.35E-13	2.84771	2.25716
C16orf74	1.21522	1.35E-13	1.41202	0.984839
COX4I1	1.13234	1.35E-13	10.8335	9.45052
APRT	1.25928	1.35E-13	4.97599	3.74558
DEF8	1.12875	1.35E-13	1.88827	1.55881
TAX1BP3	1.1458	1.35E-13	1.75464	1.40412
PSMB6	1.11142	1.35E-13	2.0546	1.74838
PFN1	1.22618	1.35E-13	16.876	13.5786
EIF5A	1.6753	1.35E-13	5.80739	3.06339
TRAPPC1	1.14937	1.35E-13	2.35652	1.92031
COPRS	1.11546	1.35E-13	1.15027	0.927695
CCL2	1.26662	4.38E-07	1.62925	1.07579
MIEN1	1.1422	1.35E-13	1.67382	1.34093

RAB5C	1.19492	1.35E-13	1.96429	1.48074
SLC25A39	1.11419	1.35E-13	1.32322	1.08512
SNF8	1.13254	1.35E-13	2.08983	1.72822
PHB	1.18252	1.35E-13	1.78926	1.35874
MRPL27	1.09669	1.35E-13	1.03886	0.859112
PSMC5	1.14115	1.35E-13	1.91933	1.55824
ICAM2	1.23012	1.35E-13	3.71221	2.8307
JPT1	1.14346	1.35E-13	4.42896	3.74783
SYNGR2	1.13849	1.35E-13	0.730588	0.520071
NDUFAF8	1.11998	1.35E-13	3.84123	3.32262
ARL16	1.1492	1.35E-13	0.66796	0.451411
MRPL12	1.32279	1.35E-13	1.9995	1.26755
MCRIP1	1.12882	1.35E-13	1.08011	0.842739
ARHGDIA	1.27714	1.35E-13	2.62958	1.84196
CENPX	1.16471	1.35E-13	1.92144	1.5083
TUBB6	1.11642	1.35E-13	2.60848	2.2322
SEC11C	1.10127	1.35E-13	0.442009	0.309402
CCBE1	1.1043	8.33E-09	0.147517	0.0391314
FKBP1A	1.18651	1.35E-13	23.5829	19.7187
SNRPB	1.12863	1.35E-13	2.2532	1.88243
MRPS26	1.11112	1.35E-13	0.762857	0.586562
CST3	1.41332	1.35E-13	7.72681	5.17467
BCL2L1	1.12607	1.35E-13	0.832329	0.627192
COMMD7	1.12102	1.35E-13	1.14078	0.909681
RALY	1.12082	1.35E-13	1.13326	0.903305
MMP24OS	1.14526	1.35E-13	2.02667	1.64279
EIF6	1.13352	1.35E-13	1.22705	0.964725
SCAND1	1.29451	1.35E-13	1.85717	1.20715
MYL9	1.10848	0.000437	0.21953	0.100184
RAB5IF	1.09828	1.35E-13	0.837912	0.673439
CTSZ	1.12084	1.35E-13	1.4862	1.21816
PSMA7	1.10196	1.35E-13	6.92663	6.19321
PPDPF	1.14137	1.35E-13	4.31079	3.65299
TMEM259	1.09752	1.35E-13	0.576582	0.436492
CNN2	1.10164	1.35E-13	0.875599	0.702559
POLR2E	1.15366	1.35E-13	1.69493	1.33598
GPX4	1.25073	1.35E-13	11.1017	8.67567
ATP5F1D	1.14201	1.35E-13	4.16669	3.52423
C19orf24	1.18007	1.35E-13	1.26736	0.921376
NDUFS7	1.22556	1.35E-13	1.31837	0.89168
TIMM13	1.14665	1.35E-13	3.79642	3.183
NFIC	1.098	1.35E-13	1.11819	0.929136
MRPL54	1.09554	1.35E-13	0.854252	0.692552
MAP2K2	1.19137	1.35E-13	1.58104	1.16645
NDUFA11	1.32392	1.35E-13	3.26795	2.22371
ALKBH7	1.10921	1.35E-13	0.807853	0.629861
ZNF358	1.11807	1.35E-13	0.500617	0.342146

PIN1	1.13164	1.35E-13	1.30357	1.03561
CDC37	1.15555	1.35E-13	1.68867	1.32674
TRIR	1.11829	1.35E-13	3.32568	2.86812
ASNA1	1.09904	1.35E-13	0.609008	0.464013
PRDX2	1.10535	1.35E-13	1.87749	1.60325
GADD45GIP1	1.10225	1.35E-13	3.63172	3.20206
LYL1	1.16987	1.35E-13	1.16396	0.84974
C19orf53	1.15947	1.35E-13	5.2194	4.36398
PKN1	1.16756	1.35E-13	1.37667	1.03559
GIPC1	1.13469	1.35E-13	0.742541	0.535697
NDUFB7	1.14375	1.35E-13	3.09827	2.5832
TPM4	1.15165	1.35E-13	6.2356	5.28283
MRPL34	1.10603	1.35E-13	1.11333	0.910743
MVB12A	1.12146	1.35E-13	0.522393	0.357512
RPL18A	1.10194	1.35E-13	65.6087	59.447
CCDC124	1.19415	1.35E-13	1.12209	0.777067
SSBP4	1.10093	1.35E-13	0.951485	0.772582
FKBP8	1.1165	1.35E-13	1.08334	0.865951
REX1BD	1.09947	1.35E-13	1.39499	1.17832
COPE	1.15229	1.35E-13	1.57207	1.23213
HOMER3	1.12065	1.35E-13	1.63287	1.34942
PDCD5	1.14541	1.35E-13	3.57653	2.99555
FXYD5	1.14686	1.35E-13	2.89041	2.39222
TMEM147	1.19815	1.35E-13	2.42245	1.85643
TBCB	1.09966	1.35E-13	1.13517	0.941669
C19orf33	1.09776	1.92E-05	0.468116	0.337375
PSMD8	1.14557	1.35E-13	1.75509	1.40499
MRPS12	1.13874	1.35E-13	1.51947	1.21252
BLVRB	1.13981	1.35E-13	0.805116	0.583701
AXL	1.17091	1.35E-13	2.187	1.72181
RABAC1	1.18278	1.35E-13	2.29792	1.78828
ERCC1	1.09609	1.35E-13	1.12002	0.934175
CALM3	1.11438	1.35E-13	2.85005	2.45487
AP2S1	1.27789	1.35E-13	7.25452	5.45947
TMEM160	1.1382	1.35E-13	1.449	1.15164
SELENOW	1.27484	1.35E-13	6.43803	4.83448
EMP3	1.38842	1.35E-13	2.50885	1.52723
RASIP1	1.09766	1.04E-08	0.586291	0.445158
BAX	1.14692	1.35E-13	2.88462	2.38701
FTL	1.31693	1.35E-13	172.681	130.884
NOSIP	1.11705	1.35E-13	0.818171	0.627651
RRAS	1.195	1.35E-13	2.32408	1.78165
JOSD2	1.12376	1.35E-13	0.942314	0.728407
RPL28	1.10468	1.35E-13	53.199	48.0632
UBE2S	1.15511	1.35E-13	2.51877	2.04626
EPN1	1.10462	1.35E-13	0.958471	0.772977
UBE2M	1.10027	1.35E-13	1.09643	0.905388

USP18	1.13069	1.35E-13	0.420157	0.256009
SLC25A1	1.1226	1.35E-13	0.556498	0.386512
DGCR6L	1.126	1.35E-13	1.01558	0.790036
SERPIND1	1.20513	1.35E-13	0.880968	0.560796
SDF2L1	1.11267	1.35E-13	1.14245	0.925503
SELENOM	1.22057	1.35E-13	1.80378	1.2971
YWHAH	1.13656	1.35E-13	1.33703	1.05623
HMOX1	1.099	1.35E-13	3.60291	3.18828
TXN2	1.11523	1.35E-13	1.26962	1.03512
LGALS1	1.3068	1.35E-13	48.5414	36.9105
TRIOBP	1.13702	1.35E-13	1.22504	0.956902
RPS19BP1	1.14705	1.35E-13	2.42027	1.98178
TSPO	1.17762	1.35E-13	5.95057	4.90221
CBR3	1.1215	1.35E-13	0.374528	0.225618
PDXK	1.11987	1.35E-13	0.660245	0.482536
CSTB	1.09994	1.35E-13	5.74046	5.12803
SUMO3	1.18539	1.35E-13	2.32003	1.8008
FAM207A	1.10995	1.35E-13	0.702257	0.533638
SPATC1L	1.16439	1.35E-13	0.331393	0.143427
MT-CO2	1.21619	1.35E-13	111.505	91.5061
MT-ATP6	1.18779	1.35E-13	32.2853	27.0229
MT-ND4	1.15243	8.98E-12	48.1429	41.6429
MT-ND5	1.14282	1.35E-13	4.21598	3.56414
MT-CYB	1.53058	1.35E-13	51.684	33.421

Supplementary Table 3. Genes up-regulated in each control cluster relative to the other control clusters

Cluster	Gene	Fold-Change	q-Value
2	NEGR1	2.280768535	1.52E-23
2	PSRC1	20.18222113	1.46E-64
2	HIST2H2AC	8.520807661	6.20E-27
2	CKS1B	6.933192791	0
2	RGS4	3.344883361	4.49E-161
2	NUF2	22.46846048	9.23E-136
2	KIF14	11.18260135	1.03E-98
2	SERTAD4-AS1	2.336459773	1.06E-22
2	NEK2	24.64180751	8.94E-89
2	DTL	4.667174936	2.26E-27
2	CENPF	23.20713437	1.30E-317
2	RRM2	10.27545899	7.29E-273
2	IL1RL1	4.060840917	2.38E-91
2	CKAP2L	19.99418063	2.34E-112
2	SPC25	21.06233128	1.27E-129
2	TUBA4A	3.924676349	2.24E-48
2	ILKAP	2.294573273	4.67E-33
2	SGO1	15.27667612	4.52E-138
2	KIF15	20.71534477	1.20E-67
2	CDCP1	2.613838299	2.87E-49
2	TMEM158	3.097584433	3.70E-48
2	SEMA3F	2.222961562	9.28E-82
2	ALCAM	2.747639145	2.06E-64
2	H1FX	3.535156836	4.33E-129
2	SMC4	10.49122687	0
2	TACC3	15.06365544	7.27E-183
2	CXCL1	2.444981713	2.87E-68
2	H2AFZ	4.114640442	0
2	FGF2	2.207283586	1.20E-15
2	HMGB2	10.43303073	0
2	CENPU	10.29737512	1.31E-156
2	PDLIM3	2.426283158	6.48E-32
2	FST	2.938973499	5.52E-59
2	CENPK	8.370474118	1.42E-160
2	TGFBI	2.224628209	2.42E-37
2	KIF20A	15.79994683	8.68E-100
2	PTTG1	7.407793829	0
2	SPDL1	7.519221651	2.09E-146
2	STC2	2.912293393	1.17E-68
2	MXD3	13.29338701	1.28E-83
2	HIST1H4C	11.87620538	1.10E-155
2	TCF19	7.428937439	3.41E-91
2	HSPA1A	2.456734304	4.29E-20
2	CENPW	6.164293351	0

2	FBXO5	11.3833535	3.54E-77
2	TWIST1	3.337217668	2.90E-11
2	ANLN	13.28261474	2.78E-241
2	SEMA3C	2.218176563	2.05E-15
2	SEMA3A	2.361151219	6.54E-15
2	DBF4	8.205000044	2.44E-133
2	EPHB6	2.887738901	1.37E-11
2	SFRP1	2.686417156	1.50E-81
2	MCM4	4.692214785	4.55E-74
2	ATAD2	7.260153408	4.30E-98
2	LY6K	2.53915644	6.73E-13
2	MSMP	2.287562005	4.72E-49
2	CKS2	9.154617427	0
2	NANS	2.37348065	1.03E-75
2	TBC1D2	2.462604636	3.35E-20
2	PHF19	5.3472854	2.07E-299
2	SAPCD2	7.620734979	1.50E-106
2	TUBB4B	8.323809898	0
2	KIF18A	20.14262227	1.11E-47
2	FEN1	5.816435069	2.16E-84
2	H2AFX	6.651779688	1.76E-204
2	MCM10	6.005593824	2.00E-42
2	DKK1	2.74913891	1.55E-110
2	ANKRD1	2.710689117	5.98E-78
2	HELLS	4.27486937	4.56E-98
2	MKI67	26.27793419	0
2	FOXM1	8.772693314	2.11E-184
2	CDCA3	16.340875	8.86E-116
2	APOLD1	9.295558385	7.84E-55
2	GPRC5A	2.426002388	8.88E-66
2	TUBA1B	3.981635115	0
2	TUBA1C	5.491467344	0
2	RACGAP1	13.83560863	2.77E-138
2	KRT7	2.683639407	8.67E-125
2	KITLG	2.333888615	4.07E-20
2	BRCA2	9.246765219	1.29E-73
2	CCNA1	4.628028201	2.59E-126
2	CKAP2	9.672350392	5.41E-231
2	DIAPH3	11.81754522	1.80E-195
2	LMO7	2.582042447	5.52E-36
2	MIS18BP1	6.688512896	1.77E-141
2	CDKN3	9.395695184	3.86E-289
2	SNAPC1	2.729143945	1.89E-41
2	SYNE2	5.756268544	1.01E-103
2	ARHGAP11B	5.959629066	1.36E-17
2	ARHGAP11A	16.51231303	2.35E-157
2	KNSTRN	8.032885162	1.07E-117

2	PCLAF	5.083519059	0
2	PRC1	11.75637208	4.64E-135
2	CCNF	9.155196174	1.13E-105
2	TNFRSF12A	2.861676357	3.26E-180
2	ARL6IP1	5.487915633	4.45E-207
2	ORC6	6.300204521	8.23E-91
2	GINS2	5.274287123	1.26E-94
2	CDT1	5.356631889	6.33E-86
2	TOP2A	28.61185732	3.55E-231
2	BRCA1	6.017164839	4.02E-99
2	KPNA2	7.852327899	2.51E-230
2	SLC9A3R1	2.620724832	6.02E-28
2	BIRC5	10.46273591	0
2	EIF4A3	2.680230629	5.78E-57
2	TYMS	7.743385207	2.18E-293
2	FAM110A	3.236849836	4.34E-23
2	CDC25B	6.179187776	1.23E-169
2	BMP2	2.227520493	6.24E-82
2	CST1	2.246886143	7.80E-67
2	ID1	2.861535513	4.11E-152
2	TPX2	19.92832823	1.26E-296
2	E2F1	4.171588043	2.80E-44
2	MYBL2	9.160077661	1.10E-232
2	UBE2C	19.90136252	5.89E-281
2	SERTAD1	2.502782453	3.01E-40
2	UBE2S	6.634791185	0
2	SERPIND1	3.291526462	1.04E-93
2	SMTN	3.065632505	7.66E-119
2	MCM5	4.421096724	3.64E-74
2	ADAMTS1	2.904857707	2.19E-45
2	MT-ND6	2.538490525	8.16E-111
6	NFIA-AS2	3.958896701	4.50E-08
6	SGIP1	3.444006796	2.20E-30
6	ITGA10	4.44975541	2.23E-69
6	EFNA1	2.777805168	2.94E-58
6	ADAMTS4	2.811033075	4.42E-71
6	KIAA0040	3.758027228	1.60E-80
6	HLX	3.820469712	3.02E-54
6	FAM89A	2.769390616	1.32E-46
6	PXDN	2.305383632	1.31E-165
6	RHOB	2.728217116	1.11E-248
6	LBH	2.506717336	2.20E-06
6	RASGRP3	10.13670085	6.27E-88
6	CDC42EP3	2.571544279	1.81E-132
6	GYPC	2.533151574	1.18E-36
6	CXCR4	8.520098363	2.00E-299
6	NRP2	3.298819975	8.51E-115

6	SH3BP5	2.243496065	4.57E-72
6	CD200	5.158937263	1.57E-17
6	TM4SF18	5.024753505	1.13E-61
6	TNFSF10	4.620254444	1.98E-53
6	KDR	3.540569959	2.06E-189
6	AFAP1L1	4.191597736	4.05E-124
6	NOTCH4	2.886400197	2.57E-79
6	TNFRSF21	2.71238927	9.12E-51
6	AKAP12	2.223836615	1.50E-62
6	RALA	2.267476559	1.97E-264
6	ERV3-1	3.223509257	3.73E-139
6	TFPI2	2.947388831	2.72E-92
6	GNG11	2.58063067	0
6	PEG10	2.224244034	7.43E-38
6	ANGPT2	3.009981058	4.27E-122
6	CSGALNACT1	5.173452832	5.44E-116
6	CLU	3.12241682	2.22E-93
6	TCIM	4.000382145	4.21E-13
6	FABP5	5.130744495	2.35E-305
6	LINC01235	2.214982877	8.16E-103
6	CNTNAP3B	2.652482488	2.27E-84
6	ADM	4.367460177	6.63E-80
6	TP53I11	4.138834558	1.25E-168
6	CHST1	10.93093523	4.56E-166
6	PDE2A	4.072729	5.73E-27
6	DEPP1	9.216096628	2.72E-259
6	VWF	3.892447985	2.44E-195
6	MGP	6.676951416	5.00E-138
6	NUDT4	3.075374065	2.30E-294
6	RGCC	5.174688626	2.54E-231
6	PCDH17	2.513092733	3.98E-08
6	EFNB2	3.132110212	8.57E-125
6	COL4A1	2.551835198	6.98E-111
6	NID2	5.645609732	2.07E-26
6	PGF	4.600091164	0
6	EIF2B2	3.029782124	1.44E-122
6	VASH1	4.700136934	3.35E-97
6	JAG2	3.132567538	4.75E-60
6	C2CD4B	2.958806337	4.13E-15
6	CCL14	4.290488745	7.26E-60
6	IGFBP4	3.020966511	8.29E-229
6	HOXB5	3.102423195	4.85E-10
6	YPEL2	3.295938603	4.43E-74
6	PECAM1	2.577429365	6.72E-253
6	RASSF2	4.322704045	2.38E-90
6	THBD	3.113720058	4.81E-98
6	CD93	2.96433816	4.02E-178

6	PPP1R16B	4.878051786	3.39E-73
6	SULF2	4.818988268	4.50E-127
6	SOX18	3.584046172	7.09E-167
6	GMFG	4.282754869	1.12E-70
6	PLA2G4C	6.073089562	5.78E-105
6	CDC42EP5	4.113715475	4.52E-125
6	CLDN5	4.589167794	1.67E-190
6	PDGFB	3.074502711	1.43E-47
6	PIM3	3.903679937	5.80E-193
7	SGIP1	2.314016396	0.00021694
7	PALMD	2.607592024	8.20E-37
7	ITGA10	2.22675613	3.93E-08
7	EFNA1	2.291388237	6.63E-14
7	ADAMTS4	2.751421404	2.35E-24
7	KIAA0040	2.510318836	7.25E-15
7	PTGS2	2.523974692	1.04E-06
7	LAMB3	2.270750965	0.00838807
7	HLX	3.308916449	4.14E-16
7	FAM89A	2.570768566	4.23E-16
7	PXDN	2.88324978	2.49E-94
7	RHOB	2.482762321	5.25E-72
7	CDC42EP3	2.377341853	2.60E-42
7	LIMS1	2.263712392	4.63E-47
7	GYPC	2.302915242	3.06E-10
7	CXCR4	2.334310145	1.40E-28
7	ITGAV	2.342039678	2.93E-40
7	NRP2	2.753696654	1.02E-27
7	SH3BP5	2.551206395	4.43E-39
7	MTRNR2L12	2.279026506	5.70E-32
7	TM4SF18	2.880516064	2.00E-09
7	HES1	2.49152279	2.88E-22
7	CYTL1	2.772569094	2.00E-51
7	KDR	2.443033166	1.15E-40
7	TLL1	2.675039252	0.0070195
7	SORBS2	2.578823498	3.54E-13
7	MCTP1	2.311088333	1.92E-06
7	HBEGF	2.29842752	3.21E-13
7	AFAP1L1	2.784486084	6.59E-27
7	SPARC	2.401140004	1.07E-100
7	NOTCH4	2.994194879	1.03E-31
7	TNFRSF21	2.391749778	1.81E-13
7	AKAP12	3.897821175	7.75E-57
7	RALA	2.714430594	5.80E-128
7	ERV3-1	2.55952516	3.29E-32
7	TFPI2	2.777880366	7.09E-29
7	GNG11	2.67336838	5.33E-156
7	PEG10	3.317471895	3.69E-28

7	SERPINE1	2.259546291	4.60E-114
7	EPHB6	2.37547079	0.00838807
7	GIMAP4	2.549212985	2.70E-53
7	APLN	3.051646077	8.94E-49
7	ANGPT2	2.909415031	9.97E-45
7	CSGALNACT1	2.547615847	1.51E-14
7	STC1	2.674975419	4.92E-11
7	CLU	2.296557308	1.91E-27
7	DUSP4	2.543862515	5.80E-44
7	LINC01235	2.223350803	1.62E-38
7	PRSS3	2.223950084	2.46E-91
7	CNTNAP3B	3.478569887	5.03E-47
7	FRMD3	2.874141813	1.89E-12
7	ENG	2.357360271	9.86E-87
7	COL5A1	2.273084547	1.52E-28
7	ADM	2.372365015	1.79E-10
7	MTRNR2L8	2.442888946	4.64E-42
7	DKK3	2.564504408	1.28E-54
7	SVIP	2.481583226	3.32E-97
7	TP53I11	2.560651931	1.84E-29
7	CHST1	2.733890285	2.90E-18
7	NEAT1	2.693295947	1.85E-85
7	PDE2A	2.266569337	0.00377474
7	PRSS23	2.377315189	1.15E-105
7	NMT2	2.25404395	2.49E-43
7	FZD8	2.436714503	1.31E-15
7	NUDT4	2.591209692	3.15E-70
7	NUAK1	2.390708197	1.12E-15
7	RGCC	2.617944356	4.58E-30
7	PCDH9	2.629910381	1.91E-10
7	LMO7	2.362181443	4.66E-11
7	COL4A2	2.352989338	1.70E-40
7	RNASE1	2.363822182	1.07E-53
7	MMP14	2.27715503	7.80E-44
7	DAAM1	2.23653971	9.89E-30
7	LTBP2	2.503877219	1.49E-47
7	PGF	3.46250528	6.01E-113
7	EIF2B2	2.746215315	1.44E-49
7	VASH1	2.236607126	2.29E-13
7	JAG2	2.371047539	2.88E-12
7	SQOR	2.42765526	4.66E-11
7	C2CD4B	2.303126907	0.0055194
7	MMP2	2.768626631	9.18E-76
7	PMP22	2.793102809	4.54E-41
7	CCL14	2.766518807	8.62E-12
7	IGFBP4	2.204627766	1.91E-55
7	ITGB3	2.789163441	8.74E-27

7	YPEL2	2.966003011	1.13E-21
7	PECAM1	2.600568612	6.00E-104
7	CDR2L	2.465001099	6.75E-14
7	LIPG	3.10185442	1.08E-09
7	RASSF2	2.486628075	3.04E-15
7	THBD	2.368426973	5.85E-22
7	CD93	2.589468024	2.86E-51
7	PPP1R16B	2.426208275	1.17E-08
7	SULF2	2.871035259	6.60E-26
7	PMEPA1	2.284351416	2.49E-31
7	SOX18	2.615823708	1.25E-33
7	FSTL3	2.284985971	2.42E-08
7	ANGPTL4	2.331076033	1.28E-07
7	COX7A1	2.580534349	0.00065023
7	KCNK6	2.376545177	2.77E-08
7	GMFG	2.469319401	6.71E-12
7	PCAT19	2.481135453	1.18E-38
7	PLA2G4C	2.639075381	3.64E-16
7	CDC42EP5	2.312969329	4.12E-20
7	CLDN5	2.640473059	1.19E-36
7	PDGFB	2.983750339	8.70E-17
7	PIM3	2.919998243	2.57E-50
7	COL6A1	2.235247694	6.22E-08
7	MT-ATP6	2.221066621	3.25E-74
7	MT-ND3	2.411606084	2.57E-63
7	MT-ND4L	2.30436244	1.13E-39
8	RGS4	2.293280792	9.39E-47
8	LAMC2	2.22599518	2.32E-30
8	DCBLD2	2.234612856	1.87E-43
8	PTX3	2.383583031	4.29E-82
8	DDIT4L	2.344600929	2.83E-11
8	HHIP	2.314530069	7.29E-125
8	TGFBI	2.439371111	9.35E-25
8	SGK1	2.278102221	4.27E-32
8	INHBA	2.271876242	1.65E-39
8	EPHB6	2.248224187	0.00011371
8	ZNF703	2.2209332	1.30E-14
8	PLAT	2.945899196	2.15E-40
8	FRMD3	2.432868258	6.78E-14
8	TBC1D2	2.20896583	4.05E-10
8	CLIC3	2.445455159	0.00229366
8	CCND1	2.270319295	2.73E-122
8	ANKRD1	2.490652973	5.36E-48
8	KRT7	2.284836501	2.08E-54
8	CDH11	2.260832712	1.02E-29
8	ITGB3	2.343396353	6.43E-28
8	FSTL3	2.403705182	9.46E-15

Supplementary Table 4. Genes down-regulated in each control cluster relative to the other control clusters

Cluster	Gene	Fold-Change	q-Value
1	CAMK2N1	0.396804699	2.42E-20
1	STMN1	0.234171649	0
1	LAPTM5	0.410681361	1.05E-91
1	NFIA-AS2	0.039243263	7.43E-31
1	PSRC1	0.061510508	1.13E-69
1	ITGA10	0.248566366	8.27E-82
1	HIST2H2AC	0.174832262	8.28E-27
1	CKS1B	0.194604659	0
1	EFNA1	0.432834901	2.78E-51
1	ADAMTS4	0.276258313	3.07E-146
1	NUF2	0.035464539	1.71E-158
1	KIAA0040	0.235953826	8.47E-130
1	KIF14	0.198825286	2.82E-74
1	NEK2	0.03983218	1.25E-100
1	DTL	0.317619119	8.62E-22
1	CENPF	0.069266414	0
1	HLX	0.175233847	4.78E-115
1	FAM89A	0.366189798	2.00E-62
1	PXDN	0.441907591	5.77E-263
1	RRM2	0.102957799	0
1	RASGRP3	0.074242876	1.59E-115
1	CDC42EP3	0.323877842	1.84E-283
1	CKAP2L	0.060412462	5.12E-124
1	GYPC	0.425713654	2.44E-48
1	CXCR4	0.112937888	0
1	SPC25	0.038215953	7.96E-155
1	NRP2	0.287599071	6.45E-165
1	TUBA4A	0.342630199	2.59E-44
1	HES6	0.44847068	8.52E-11
1	SH3BP5	0.352838384	6.28E-196
1	SGO1	0.081464809	7.46E-149
1	KIF15	0.090915887	1.87E-63
1	CDCP1	0.387112066	4.45E-83
1	TMEM158	0.263212887	5.18E-129
1	CD200	0.218704028	8.91E-19
1	H1FX	0.358805717	2.05E-155
1	TM4SF18	0.150672528	2.07E-92
1	SMC4	0.139008483	0
1	TNFSF10	0.343611716	4.85E-37
1	HES1	0.436016963	5.09E-70
1	TACC3	0.110827874	6.55E-194
1	KDR	0.340177221	3.85E-213
1	H2AFZ	0.266633263	0
1	HMGB2	0.147791829	0

1	CENPU	0.141715964	2.12E-165
1	CENPK	0.20448361	3.75E-141
1	KIF20A	0.06265448	1.54E-116
1	AFAP1L1	0.251351478	1.80E-181
1	PTTG1	0.137710092	0
1	SPDL1	0.302867001	1.31E-93
1	MXD3	0.130473133	3.28E-85
1	HIST1H4C	0.154049112	3.35E-236
1	TCF19	0.143986755	1.30E-111
1	NOTCH4	0.329281055	2.02E-118
1	TNFRSF21	0.43775121	6.42E-49
1	CENPW	0.166420968	0
1	AKAP12	0.291745318	4.00E-234
1	FBXO5	0.161131279	1.51E-71
1	ANLN	0.113058297	1.25E-245
1	RALA	0.430674593	0
1	DBF4	0.243538527	8.87E-105
1	TFPI2	0.388900881	3.54E-149
1	GNG11	0.354496769	0
1	PEG10	0.213892418	1.32E-207
1	GIMAP4	0.352379983	4.11E-250
1	APLN	0.424065692	2.32E-156
1	ANGPT2	0.318840731	6.30E-217
1	CSGALNACT1	0.2506312	9.38E-121
1	STC1	0.289200074	3.97E-62
1	CLU	0.384379932	2.96E-120
1	TCIM	0.356730113	1.00E-09
1	MCM4	0.274724404	2.23E-81
1	FABP5	0.155768255	0
1	ATAD2	0.222323263	2.20E-94
1	MSMP	0.438938644	8.39E-70
1	CNTNAP3B	0.410471099	1.16E-100
1	CKS2	0.24355598	0
1	NANS	0.438470664	1.99E-105
1	PHF19	0.283059135	1.95E-301
1	SAPCD2	0.231575093	1.06E-93
1	TUBB4B	0.181142396	0
1	ADM	0.247523968	2.82E-105
1	SVIP	0.393559385	0
1	KIF18A	0.124016331	5.09E-39
1	TP53I11	0.302032686	3.63E-184
1	CHST1	0.050929716	1.41E-262
1	FEN1	0.240634514	5.72E-86
1	GAL	0.395949715	2.25E-12
1	PDE2A	0.312096945	1.50E-25
1	H2AFX	0.281317328	7.00E-170
1	MCM10	0.243308231	3.68E-39

1	DEPP1	0.105534945	0
1	HELLS	0.309751423	2.43E-110
1	MKI67	0.036840614	0
1	FOXM1	0.130691278	9.19E-226
1	VWF	0.348563274	6.05E-200
1	CDCA3	0.075351292	1.09E-126
1	CLEC2B	0.314374345	4.77E-155
1	APOLD1	0.263763348	6.70E-35
1	MGP	0.204410062	1.82E-180
1	TUBA1B	0.254475273	0
1	TUBA1C	0.269452308	0
1	RACGAP1	0.139963852	1.07E-121
1	NUDT4	0.441763534	1.64E-260
1	BRCA2	0.182067384	4.42E-65
1	CCNA1	0.351197202	2.34E-110
1	RGCC	0.194751213	1.27E-305
1	CKAP2	0.200474589	2.18E-228
1	PCDH17	0.435515927	2.57E-09
1	DIAPH3	0.177136536	1.40E-157
1	LMO7	0.380232737	1.28E-52
1	EFNB2	0.44041398	4.87E-107
1	MIS18BP1	0.275025197	2.07E-119
1	NID2	0.193008933	1.98E-30
1	CDKN3	0.08806449	0
1	SYNE2	0.211953546	5.44E-132
1	PGF	0.166420736	0
1	EIF2B2	0.299551752	8.43E-267
1	VASH1	0.338624857	3.11E-83
1	ARHGAP11B	0.222538016	6.53E-17
1	ARHGAP11A	0.116635265	1.53E-143
1	KNSTRN	0.28026817	2.11E-82
1	GCHFR	0.429850626	4.77E-33
1	C2CD4B	0.250402982	6.26E-32
1	PCLAF	0.242516262	0
1	PRC1	0.086833147	3.52E-159
1	CCNF	0.234104137	1.04E-90
1	SOCS1	0.452258815	1.38E-15
1	ARL6IP1	0.320962161	9.11E-208
1	ORC6	0.200210673	6.12E-104
1	CRNDE	0.414857352	1.43E-17
1	MT2A	0.403244428	0
1	GINS2	0.196137474	9.38E-130
1	CDT1	0.257104058	2.74E-89
1	CCL14	0.203307829	4.37E-94
1	TOP2A	0.050584873	7.69E-261
1	IGFBP4	0.337309792	0
1	BRCA1	0.298716397	1.10E-69

1	HOXB5	0.399958736	2.74E-08
1	YPEL2	0.423617009	5.65E-57
1	PECAM1	0.396261795	0
1	KPNA2	0.252293268	3.80E-268
1	BIRC5	0.100192183	0
1	SOCS3	0.420544847	3.52E-33
1	TYMS	0.167641938	0
1	PMAIP1	0.340436291	2.91E-74
1	CDC25B	0.322768545	1.41E-130
1	RASSF2	0.254743378	9.11E-113
1	THBD	0.343428806	3.31E-145
1	CD93	0.264071071	0
1	TPX2	0.084551394	0
1	E2F1	0.310056507	5.38E-44
1	PPP1R16B	0.247125619	6.05E-71
1	MYBL2	0.144675467	1.68E-256
1	UBE2C	0.040065727	0
1	SULF2	0.222573575	4.67E-182
1	SOX18	0.351660409	1.26E-152
1	GMFG	0.134428132	1.88E-158
1	PLA2G4C	0.233093415	2.95E-108
1	CDC42EP5	0.39169352	5.17E-90
1	UBE2S	0.226112115	0
1	CLDN5	0.165200558	0
1	SMTN	0.438502286	9.43E-123
1	MCM5	0.272722934	7.68E-95
1	H1F0	0.398069082	1.09E-27
1	PDGFB	0.401951773	6.04E-42
1	PIM3	0.252882733	0
2	SGIP1	0.20327417	3.29E-47
2	ITGA10	0.411071456	1.08E-28
2	RASGRP3	0.117398986	1.34E-77
2	CXCR4	0.126239824	2.09748178719839e-314
2	TNFSF10	0.36729788	2.61E-25
2	ERV3-1	0.415737139	2.04E-87
2	CSGALNACT1	0.310122326	1.36E-67
2	CHST1	0.126796476	1.57E-163
2	DEPP1	0.091744097	3.32E-268
2	MGP	0.351382122	6.12E-73
2	RGCC	0.317888735	4.55E-127
2	NID2	0.353520295	1.03E-10
2	YPEL2	0.419504796	3.57E-45
2	PPP1R16B	0.199702259	8.96E-72
2	SULF2	0.390911865	1.08E-62
2	PLA2G4C	0.149504873	4.31E-134
2	PDGFB	0.37424508	3.44E-35
3	HSPG2	0.379245799	0

3	SGIP1	0.175898834	3.07E-60
3	PALMD	0.377430136	9.49E-134
3	ITGA10	0.193662292	1.52E-96
3	EFNA1	0.371972849	1.70E-55
3	SELP	0.217111964	2.62E-97
3	KIAA0040	0.325627863	1.86E-74
3	HLX	0.350488995	5.90E-39
3	PXDN	0.428408058	2.90E-267
3	RHOB	0.299559529	0
3	LBH	0.314160605	1.76E-11
3	RASGRP3	0.031051143	2.22E-139
3	CXCR4	0.080828567	0
3	ITGAV	0.437388365	2.80E-141
3	SLC40A1	0.350794482	1.04E-42
3	NRP2	0.337271613	1.54E-116
3	FN1	0.297639097	0
3	LINC00607	0.305535036	2.46E-54
3	CD200	0.392820664	2.06E-06
3	TM4SF18	0.202330958	6.63E-67
3	TNFSF10	0.117386956	4.39E-104
3	KDR	0.30397658	9.99E-224
3	TLL1	0.281099314	5.92E-16
3	MCTP1	0.3795882	8.32E-29
3	AFAP1L1	0.330976605	4.52E-111
3	SPARC	0.378832685	0
3	TNFRSF21	0.367403888	1.59E-61
3	RALA	0.449548385	0
3	ERV3-1	0.26762927	1.39E-212
3	SAT1	0.378862784	8.32105360725827e-320
3	BGN	0.28784095	9.88550132869361e-315
3	CSGALNACT1	0.139968739	1.35E-186
3	CLU	0.230729912	2.11E-243
3	TCIM	0.28411046	5.98E-12
3	FABP5	0.325711235	0
3	CNTNAP3B	0.300055435	3.51E-156
3	ALDH1A1	0.305875374	8.27E-71
3	COL5A1	0.384368248	1.17E-130
3	ADM	0.327211787	2.36E-62
3	DKK3	0.444510354	1.44E-159
3	TP53I11	0.243451246	7.69E-220
3	CHST1	0.068391089	2.73E-240
3	NEAT1	0.204820171	0
3	PDE2A	0.213797888	2.96E-36
3	DEPP1	0.039210444	0
3	ACTA2	0.379219499	2.85E-44
3	CCND2	0.359613628	6.79E-88
3	VWF	0.197548877	0

3	MGP	0.085639904	1.10E-300
3	MDM2	0.413034169	9.47E-147
3	NUDT4	0.328889099	0
3	NUAK1	0.437130502	1.05E-51
3	RGCC	0.290952121	8.78E-170
3	KCTD12	0.35100673	0
3	EFNB2	0.293883958	4.62E-180
3	COL4A1	0.357855204	3.83E-172
3	NID2	0.161822349	3.25E-30
3	PGF	0.301412721	0
3	VASH1	0.222329043	1.20E-129
3	DIO2	0.201801988	5.26E-21
3	JAG2	0.284038241	1.40E-80
3	C2CD4B	0.436968841	6.01E-09
3	MMP2	0.316491557	0
3	CCL2	0.342403994	2.77E-101
3	CCL14	0.183211418	8.23E-87
3	IGFBP4	0.370418916	3.48E-297
3	YPEL2	0.260906111	8.72E-109
3	PECAM1	0.380098516	0
3	RASSF2	0.277027283	3.62E-88
3	PPP1R16B	0.10452168	1.03E-133
3	SULF2	0.195729191	5.03E-181
3	SOX18	0.320209779	5.47E-150
3	ANGPTL4	0.250777325	2.53E-57
3	ICAM1	0.441368092	3.44E-31
3	GDF15	0.375962163	2.68E-174
3	COX7A1	0.35887637	4.25E-21
3	PCAT19	0.444746467	9.08E-123
3	PLA2G4C	0.092232609	1.57E-189
3	CDC42EP5	0.278253975	1.38E-131
3	CLDN5	0.356890447	3.08E-122
3	PDGFB	0.310866493	4.80E-58
3	PIM3	0.398479582	4.34E-143
3	MX1	0.370515187	5.22E-07
3	COL6A1	0.272605376	4.16E-68
4	STMN1	0.378224379	1.74E-214
4	NFIA-AS2	0.178561018	2.29E-08
4	PSRC1	0.143803805	1.29E-29
4	ITGA10	0.220362983	4.99E-56
4	HIST2H2AC	0.127297836	1.29E-21
4	CKS1B	0.232999343	3.09E-266
4	ADAMTS4	0.183330078	6.70E-139
4	NUF2	0.038669931	1.01E-130
4	KIAA0040	0.253183533	9.25E-68
4	KIF14	0.166392416	9.90E-54
4	NEK2	0.044761231	1.57E-80

4	DTL	0.203284852	4.04E-25
4	CENPF	0.062230615	0
4	HLX	0.300714324	8.73E-34
4	FAM89A	0.407178233	1.94E-25
4	RRM2	0.141579903	9.48E-203
4	LBH	0.448734848	0.007229833
4	RASGRP3	0.02315723	4.32E-132
4	CDC42EP3	0.323860671	1.20E-142
4	CKAP2L	0.074328678	7.41E-79
4	GYPC	0.442835008	4.79E-24
4	CXCR4	0.137169735	1.83E-270
4	SPC25	0.047750489	2.03E-120
4	NRP2	0.31432013	2.65E-81
4	TUBA4A	0.447826477	3.62E-13
4	SH3BP5	0.350030793	1.04E-107
4	SGO1	0.088865657	4.24E-97
4	KIF15	0	8.00E-100
4	CDCP1	0.304760766	2.21E-64
4	TMEM158	0.23716566	1.87E-87
4	CD200	0.238511108	6.89E-09
4	H1FX	0.428887552	3.13E-56
4	TM4SF18	0.207573177	9.65E-43
4	SMC4	0.151868907	7.10E-271
4	TNFSF10	0.370845638	1.39E-16
4	TACC3	0.173982531	2.39E-97
4	KDR	0.276758653	3.94E-152
4	H2AFZ	0.329691525	0
4	HMGB2	0.155942353	1.40E-296
4	CENPU	0.138927147	1.25E-113
4	CENPK	0.185342792	2.12E-96
4	KIF20A	0.058384214	6.94E-93
4	AFAP1L1	0.18627583	1.41E-157
4	PTTG1	0.192830632	1.63E-285
4	SPDL1	0.269308992	3.32E-64
4	MXD3	0.160670177	5.10E-48
4	HIST1H4C	0.164099948	1.61E-220
4	TCF19	0.226318445	1.53E-39
4	NOTCH4	0.294134329	4.80E-72
4	CENPW	0.208728102	2.45E-254
4	AKAP12	0.240926306	8.77E-215
4	FBXO5	0.18944108	2.50E-35
4	ANLN	0.124059977	2.22E-155
4	DBF4	0.229401308	7.23E-67
4	TFPI2	0.420190515	1.03E-60
4	PEG10	0.299304711	1.12E-81
4	APLN	0.439247593	3.27E-77
4	CSGALNACT1	0.319101902	1.05E-48

4	STC1	0.301237734	3.98E-33
4	TCIM	0.310176417	5.83E-06
4	MCM4	0.2532648	9.17E-55
4	FABP5	0.298482315	1.78E-307
4	ATAD2	0.197540015	3.26E-61
4	CNTNAP3B	0.44918228	2.37E-42
4	CKS2	0.284488034	1.49E-211
4	PHF19	0.328540488	4.72E-131
4	SAPCD2	0.193883802	2.14E-66
4	TUBB4B	0.159553999	0
4	ADM	0.293493916	2.53E-46
4	SVIP	0.388299638	3.34E-295
4	KIF18A	0.078094062	8.54E-36
4	TP53I11	0.352104334	3.78E-76
4	CHST1	0.048708931	3.27E-227
4	FEN1	0.287786582	4.25E-39
4	PDE2A	0.384752943	1.27E-08
4	H2AFX	0.260828447	1.28E-108
4	MCM10	0.113518173	2.94E-50
4	DEPP1	0.123549796	5.15E-267
4	HELLS	0.353402015	1.77E-43
4	MKI67	0.039613177	0
4	FOXM1	0.131135247	2.82E-141
4	CDCA3	0.097704195	1.26E-81
4	APOLD1	0.231978851	2.65E-23
4	MGP	0.447329967	1.94E-31
4	TUBA1B	0.126800747	0
4	TUBA1C	0.251573754	0
4	RACGAP1	0.139874898	4.75E-74
4	BRCA2	0.083872513	1.19E-75
4	CCNA1	0.335529089	4.63E-69
4	RGCC	0.240101483	1.86E-143
4	CKAP2	0.269624275	3.57E-104
4	PCDH17	0.363808242	3.22E-05
4	DIAPH3	0.124421049	5.29E-137
4	LMO7	0.260545784	5.08E-63
4	MIS18BP1	0.323749949	2.16E-53
4	NID2	0.178837215	2.71E-19
4	CDKN3	0.126912386	1.42E-234
4	SYNE2	0.225087138	1.75E-71
4	PGF	0.18689555	0
4	EIF2B2	0.34178337	2.09E-126
4	VASH1	0.378216677	6.36E-37
4	JAG2	0.451687619	2.73E-19
4	ARHGAP11B	0.215063727	2.12E-09
4	ARHGAP11A	0.119074555	6.19E-98
4	KNSTRN	0.312418056	2.99E-37

4	PCLAF	0.335338396	1.34E-171
4	PRC1	0.085964436	4.60E-115
4	CCNF	0.184578528	1.32E-71
4	ARL6IP1	0.389722675	2.76E-83
4	ORC6	0.239681777	2.07E-47
4	GINS2	0.279856142	2.68E-46
4	CDT1	0.296457075	8.40E-41
4	PMP22	0.418416716	7.14E-67
4	CCL14	0.426907248	2.46E-14
4	TOP2A	0.047901089	1.20E-255
4	IGFBP4	0.400019728	4.30E-148
4	BRCA1	0.184385353	1.06E-79
4	KPNA2	0.297730446	5.31E-143
4	CDR2L	0.427061555	9.77E-22
4	BIRC5	0.120270619	1.61E-269
4	TYMS	0.242810988	2.10E-176
4	PMAIP1	0.408600618	2.58E-26
4	CDC25B	0.303795731	2.69E-80
4	RASSF2	0.371333053	1.18E-33
4	THBD	0.267947247	4.16E-116
4	CD93	0.279134204	2.41E-206
4	TPX2	0.05550831	0
4	E2F1	0.219858825	3.80E-38
4	PPP1R16B	0.261550185	7.04E-35
4	MYBL2	0.145947364	6.53E-164
4	UBE2C	0.054619359	7.82E-297
4	SULF2	0.242937045	3.09E-100
4	GMFG	0.281418897	8.60E-41
4	PLA2G4C	0.284528754	8.74E-49
4	CDC42EP5	0.337016947	1.73E-62
4	UBE2S	0.259993067	0
4	CLDN5	0.183745971	2.87E-241
4	SERPIND1	0.40685787	1.82E-57
4	SMTN	0.379901034	3.72E-91
4	MCM5	0.3160408	3.59E-42
4	PIM3	0.298671507	2.49E-160
5	STMN1	0.379872482	1.37E-261
5	NEGR1	0.130025025	9.31E-110
5	NEXN	0.186307859	4.65E-126
5	PSRC1	0.064231125	1.00E-56
5	HIST2H2AC	0.280845382	7.63E-09
5	CKS1B	0.17658645	0
5	RGS4	0.083790349	0
5	RGS5	0.371446738	3.63E-250
5	NUF2	0.045673583	3.85E-131
5	KIF14	0.241502669	4.35E-39
5	NEK2	0.011696141	5.56E-111

5	DTL	0.119289887	2.10E-43
5	CENPF	0.048212234	0
5	RRM2	0.084100014	1.76283358854836e-312
5	IL1RL1	0.125536562	1.27E-253
5	CKAP2L	0.050029734	1.02E-112
5	LYPD1	0.27809971	1.38E-154
5	SPC25	0.045078875	7.05E-131
5	TUBA4A	0.280744387	5.05E-39
5	SERPINE2	0.385676008	4.71E-66
5	SGO1	0.044320416	4.82E-157
5	KIF15	0.04531489	1.08E-68
5	CDCP1	0.425100816	1.13E-40
5	TMEM158	0.359782458	6.43E-50
5	SEMA3F	0.37543066	1.10E-122
5	DCBLD2	0.329643201	7.92E-124
5	ALCAM	0.341579643	2.31E-64
5	PLOD2	0.410232499	3.40E-112
5	PTX3	0.180482833	0
5	SMC4	0.198311566	3.63E-237
5	MELTF	0.371864405	4.11E-17
5	TACC3	0.111239413	8.41E-155
5	CXCL8	0.367929126	9.93E-87
5	CXCL1	0.279774257	9.25E-164
5	H2AFZ	0.28126415	0
5	DDIT4L	0.293759028	3.37E-33
5	FGF2	0.367559201	8.15E-20
5	MGARP	0.20319908	9.59E-227
5	HHIP	0.308466667	0
5	HMGB2	0.200377061	2.09E-266
5	CENPU	0.068436023	1.63E-178
5	FST	0.173669193	1.37E-176
5	CENPK	0.092826513	7.46E-191
5	TGFBI	0.268220058	3.67E-83
5	KIF20A	0.018298402	5.21E-136
5	PTTG1	0.10260779	0
5	SPDL1	0.27154741	3.19E-72
5	STC2	0.340073292	2.50E-66
5	MXD3	0.186496737	3.34E-47
5	HIST1H4C	0.231148492	4.58E-180
5	TCF19	0.115492503	2.88E-92
5	CENPW	0.1240529	0
5	RSPO3	0.349413492	3.73E-32
5	SGK1	0.350581818	2.49E-68
5	FBXO5	0.111200994	4.27E-69
5	ANLN	0.058838505	2.04E-267
5	INHBA	0.243888661	7.15E-150
5	SEMA3C	0.149098088	6.62E-73

5	SEMA3A	0.241165004	6.16E-36
5	DBF4	0.224553122	3.42E-82
5	EPHB6	0.269946013	4.12E-15
5	SFRP1	0.319295808	6.95E-114
5	PLAT	0.323574266	1.43E-124
5	MCM4	0.189825613	4.45E-87
5	ATAD2	0.079450548	6.39E-151
5	LY6K	0.274592133	3.46E-21
5	PRSS3	0.386160087	4.56302132762196e-314
5	CKS2	0.224952892	9.93611778360256e-312
5	GADD45G	0.219673706	8.96E-17
5	TBC1D2	0.312928488	9.25E-30
5	GABBR2	0.35647216	2.13E-88
5	PHF19	0.25824152	1.13E-219
5	PTGS1	0.416791402	2.38E-35
5	CLIC3	0.201869249	4.47E-16
5	SAPCD2	0.168469957	2.55E-88
5	TUBB4B	0.216769541	0
5	BDNF	0.358702493	2.77E-13
5	KIF18A	0.020365045	3.19E-60
5	FEN1	0.204156098	1.49E-67
5	GAL	0.445342982	4.55E-05
5	BIRC3	0.286383442	9.72E-07
5	TAGLN	0.237612015	7.89E-43
5	H2AFX	0.365831548	2.01E-70
5	MCM10	0.089228289	1.98E-65
5	NMT2	0.417221574	1.00E-121
5	DKK1	0.157597026	0
5	ADIRF	0.453403737	7.63E-196
5	ANKRD1	0.143790687	0
5	HELLS	0.256708013	1.72E-90
5	MKI67	0.037770648	0
5	FOXM1	0.073838395	1.57E-241
5	CDCA3	0.053615936	8.82E-122
5	APOLD1	0.270089601	3.92E-22
5	GPRC5A	0.192243663	6.77E-176
5	TUBA1B	0.131529253	0
5	TUBA1C	0.243320318	0
5	RACGAP1	0.097591476	1.63E-115
5	KRT7	0.150896636	0
5	RAB3IP	0.4402002	1.01E-49
5	KITLG	0.404445983	1.97E-16
5	BRCA2	0.152178357	2.35E-54
5	CCNA1	0.164016374	5.42E-183
5	CKAP2	0.272437948	3.39E-119
5	DIAPH3	0.069631086	6.18E-216
5	MIS18BP1	0.406274146	3.63E-40

5	CDKN3	0.04874717	0
5	ARHGAP11B	0.238527591	1.07E-09
5	ARHGAP11A	0.068769589	1.97E-149
5	KNSTRN	0.298292525	8.01E-47
5	PCLAF	0.140781961	0
5	PRC1	0.079969348	1.36E-132
5	ALDH1A3	0.348410629	4.74E-121
5	CCNF	0.257128947	1.09E-55
5	TNFRSF12A	0.350454799	9.47E-163
5	ORC6	0.148941293	7.50E-92
5	MT2A	0.211878471	0
5	MT1E	0.229632056	6.69E-90
5	CDH11	0.321359208	4.03E-93
5	GINS2	0.125558182	1.56E-139
5	CDT1	0.237909402	1.95E-62
5	TOP2A	0.045931503	1.28E-259
5	BRCA1	0.149539138	5.74E-105
5	KPNA2	0.33416448	4.49E-137
5	SLC9A3R1	0.454148242	7.31E-16
5	BIRC5	0.064932678	0
5	TYMS	0.21168087	2.12E-223
5	SERPINB2	0.297139628	5.92E-27
5	DSEL	0.395869374	2.89E-41
5	CST1	0.358181262	2.19E-103
5	TPX2	0.067501123	4.68374232257502e-320
5	E2F1	0.187528121	1.79E-56
5	MYL9	0.388090215	3.27E-17
5	MYBL2	0.060644227	1.11399406970069e-310
5	UBE2C	0.04984098	8.4790191157001e-310
5	FSTL3	0.444486712	6.03E-18
5	GADD45B	0.259654321	6.45E-49
5	UBE2S	0.248945279	0
5	TXNRD2	0.293761015	2.06E-229
5	SERPIND1	0.126343849	8.56512203630385e-320
5	MCM5	0.232220781	2.12E-75
5	ADAMTS1	0.364260408	7.49E-37
5	MT-ND6	0.452996268	1.86E-76
6	PSRC1	0.120779899	5.17E-47
6	HIST2H2AC	0.428880476	7.74E-06
6	CKS1B	0.199531081	0
6	RGS4	0.063707435	0
6	RGS5	0.453926084	7.88E-188
6	NUF2	0.037670559	1.28E-150
6	KIF14	0.239598368	2.54E-52
6	NEK2	0.058330265	9.04E-87
6	DTL	0.14938073	1.06E-43
6	CENPF	0.055053357	0

6	RRM2	0.074337182	0
6	IL1RL1	0.114395405	3.47E-307
6	CKAP2L	0.030893125	2.67E-138
6	LYPD1	0.229290746	8.83E-264
6	SPC25	0.032503539	8.77E-154
6	SGO1	0.062969196	1.48E-154
6	KIF15	0.0373742	3.08E-79
6	CDCP1	0.334478595	8.97E-90
6	SEMA3F	0.391437994	7.61E-157
6	DCBLD2	0.31304285	1.09E-182
6	ALCAM	0.439180097	2.23E-54
6	PLOD2	0.424486261	2.11E-142
6	PTX3	0.102526989	0
6	SMC4	0.275721137	3.77E-205
6	CLDN11	0.333021877	5.86E-150
6	TACC3	0.180939841	1.04E-125
6	CXCL8	0.32288395	5.12E-141
6	CXCL1	0.219775255	4.72E-287
6	CXCL3	0.438728478	1.32E-10
6	H2AFZ	0.415761736	0
6	DDIT4L	0.288648671	3.76E-46
6	MGARP	0.162543792	0
6	HHIP	0.385152154	0
6	HMGB2	0.204481878	2.41623199351178e-317
6	CENPU	0.131401678	2.77E-151
6	FST	0.208983305	6.90E-174
6	CENPK	0.168478441	7.09E-150
6	KIF20A	0.062742386	1.97E-109
6	PTTG1	0.136402821	0
6	SPDL1	0.288482103	6.57E-89
6	STC2	0.332492592	8.47E-96
6	MXD3	0.314702144	1.35E-31
6	HIST1H4C	0.371039991	6.47E-112
6	TCF19	0.131491926	5.85E-108
6	CENPW	0.221226299	0
6	RSPO3	0.386438007	5.86E-34
6	SGK1	0.287673684	1.95E-127
6	FBXO5	0.217543334	5.66E-48
6	ANLN	0.069456703	1.61E-273
6	INHBA	0.261555444	4.69E-178
6	SEMA3C	0.125044874	3.01E-103
6	SEMA3A	0.137603258	3.78E-77
6	DBF4	0.284707571	2.86E-77
6	LRRC17	0.427560844	1.38E-13
6	EPHB6	0.287453754	4.42E-20
6	SFRP1	0.442423199	2.32E-77
6	PLAT	0.276329168	9.88E-195

6	MCM4	0.251640946	7.70E-79
6	ATAD2	0.195262023	3.05E-90
6	CKS2	0.242018668	0
6	GADD45G	0.191854225	1.41E-25
6	TBC1D2	0.207920835	2.81E-68
6	GABBR2	0.373995095	6.21E-112
6	PHF19	0.329516719	1.43E-204
6	CLIC3	0.238594221	2.42E-17
6	SAPCD2	0.360839566	5.57E-42
6	TUBB4B	0.353783982	1.08E-257
6	BDNF	0.266505243	1.70E-32
6	KIF18A	0.025209955	2.52E-61
6	FEN1	0.254662192	1.56E-69
6	BIRC3	0.365783992	1.29E-06
6	TAGLN	0.284827209	4.73E-42
6	MCM10	0.076053837	1.77E-81
6	NMT2	0.41402123	6.06E-165
6	DKK1	0.12069096	0
6	ADIRF	0.407608093	0
6	ANKRD1	0.105403128	0
6	HELLS	0.299277811	1.86E-89
6	MKI67	0.032709573	0
6	FOXM1	0.10420493	7.86E-225
6	CDCA3	0.071450676	7.78E-119
6	APOLD1	0.411725257	1.11E-12
6	GPRC5A	0.174674247	4.35E-264
6	MGST1	0.406598975	9.66E-170
6	TUBA1B	0.27419759	0
6	TUBA1C	0.378680187	0
6	RACGAP1	0.106358419	6.11E-127
6	KRT7	0.191268021	0
6	BRCA2	0.166643981	1.05E-60
6	CCNA1	0.217214479	2.63E-183
6	CKAP2	0.348687094	8.96E-101
6	DIAPH3	0.049506139	7.62E-275
6	CDKN3	0.092400182	0
6	ARHGAP11B	0.212928707	5.35E-15
6	ARHGAP11A	0.073212473	4.67E-170
6	KNSTRN	0.346622912	7.60E-49
6	PCLAF	0.150958695	0
6	PRC1	0.076350151	7.02E-157
6	CCNF	0.374174663	3.71E-41
6	TNFRSF12A	0.358792316	5.11E-223
6	ORC6	0.179996469	1.29E-100
6	MT2A	0.321278694	0
6	MT1E	0.240583169	6.43E-107
6	CDH11	0.361199734	5.69E-99

6	GINS2	0.157345747	5.11E-138
6	CDT1	0.251506379	2.24E-77
6	TOP2A	0.039351455	1.17E-272
6	BRCA1	0.265637477	3.87E-74
6	KPNA2	0.453007405	3.81E-96
6	BIRC5	0.080797403	0
6	TYMS	0.24027482	4.10E-249
6	SERPINB2	0.193231863	3.17E-63
6	TPX2	0.082438609	0
6	E2F1	0.265395215	6.94E-50
6	MYBL2	0.087689282	3.10E-298
6	UBE2C	0.067313907	1.02E-307
6	FSTL3	0.439489854	1.02E-24
6	GADD45B	0.265523454	2.10E-66
6	UBE2S	0.308220064	0
6	TXNRD2	0.290840572	5.50E-304
6	SERPIND1	0.104032967	0
6	MCM5	0.440678722	3.52E-34
6	ADAMTS1	0.255671158	8.77E-87
7	NUF2	0.346060982	1.03E-11
8	PSRC1	0.184825921	7.46E-23
8	NUF2	0.295588625	1.99E-24
8	NEK2	0.273020863	1.54E-18
8	CENPF	0.32171308	4.42E-102
8	RASGRP3	0.104551951	7.58E-56
8	CKAP2L	0.324447869	1.02E-19
8	CXCR4	0.216212096	4.93E-161
8	SPC25	0.351861112	3.24E-18
8	KIF15	0.374076161	1.88E-07
8	TM4SF18	0.369539622	8.57E-15
8	TNFSF10	0.369332626	1.78E-15
8	HMGB2	0.442547941	3.58E-58
8	MXD3	0.437341495	2.30E-10
8	ADM	0.373255032	2.50E-31
8	CHST1	0.122085588	9.91E-147
8	DEPP1	0.100386584	4.53E-306
8	MKI67	0.257242614	3.32E-126
8	CDCA3	0.410237096	4.88E-13
8	MGP	0.305840608	5.94E-85
8	RGCC	0.376770537	8.17E-68
8	NID2	0.335440926	1.33E-07
8	TOP2A	0.213364655	1.86E-117
8	HOXB5	0.42178223	0.003104668
8	TPX2	0.340267579	4.04E-75
8	PPP1R16B	0.260483087	1.63E-33
8	UBE2C	0.309443967	4.53E-71
8	PLA2G4C	0.415717387	2.23E-24

Supplementary Table 5. Genes up-regulated in each PAH cluster relative to the other PAH clusters

Cluster	Gene	Fold-Change	q-Value
1	SELP	2.247975927	5.07E-06
1	CXCL8	2.203931818	9.21E-34
1	TAGLN	2.458351567	6.02E-24
2	TOP2A	23.31466095	3.18E-299
2	UBE2C	20.81621634	0
2	KIF18A	20.80020025	3.16E-46
2	MKI67	17.37921703	0
2	NEK2	17.1630725	2.83E-84
2	PSRC1	17.09632113	2.95E-71
2	NUF2	16.73112233	1.31E-152
2	CKAP2L	15.91362108	4.26E-133
2	CENPF	15.54628052	3.58E-316
2	SPC25	14.05552696	4.99E-104
2	TPX2	13.99175497	3.08E-317
2	SGO1	13.07892601	3.57E-153
2	MXD3	12.87938096	6.77E-105
2	ARHGAP11A	12.76487106	9.25E-170
2	CDCA3	12.42640273	2.71E-114
2	KIF15	12.38307661	3.61E-55
2	RACGAP1	10.81245872	1.46E-148
2	HMGB2	10.79564451	0
2	KIF14	10.169082	1.80E-101
2	HIST1H4C	10.1167781	2.28E-195
2	TACC3	9.961113549	2.47E-151
2	ANLN	9.905285615	1.71E-249
2	KIF20A	9.150493518	8.10E-59
2	HIST2H2AC	8.906419374	5.09E-43
2	CKS2	8.828594291	0
2	CCNF	8.31462034	4.60E-129
2	CKAP2	8.237703068	8.29E-229
2	PRC1	8.061838132	9.69E-97
2	SMC4	7.682361918	0
2	ARHGAP11B	7.654653179	2.78E-43
2	RRM2	7.627822051	2.50E-254
2	BIRC5	7.540539924	0
2	DIAPH3	7.396444493	1.55E-162
2	KNSTRN	7.144952007	1.77E-115
2	FOXM1	7.130066445	1.13E-194
2	FBXO5	7.034521326	1.41E-71
2	CDKN3	6.790643429	4.11E-300
2	UBE2S	6.567372667	0
2	KPNA2	6.533652217	7.55E-246
2	SAPCD2	6.49308077	1.04E-106
2	CKS1B	6.439434669	0

2	MYBL2	6.421317001	2.45E-209
2	MIS18BP1	6.285549285	1.57E-141
2	BRCA2	6.282839016	1.33E-55
2	DBF4	6.24986922	1.87E-131
2	CENPU	5.904311148	3.98E-134
2	CENPK	5.901209694	1.93E-137
2	ARL6IP1	5.821984443	4.24E-267
2	CDC25B	5.795173372	1.49E-168
2	H2AFX	5.776366517	6.25E-215
2	APOLD1	5.64603371	1.25E-28
2	TUBB4B	5.341418678	1.77E-268
2	TCF19	5.117209982	3.68E-88
2	SPDL1	5.047994457	3.94E-107
2	PTTG1	5.027165723	0
2	PCLAF	4.951901524	0
2	CENPW	4.759301471	0
2	TYMS	4.737219201	1.28E-232
2	SYNE2	4.684905129	7.89E-85
2	ATAD2	4.485218382	2.02E-80
2	ORC6	4.481308417	1.10E-74
2	PHF19	4.356214202	3.49E-302
2	BRCA1	4.166279	9.96E-81
2	TUBA1C	4.080496561	0
2	STMN1	3.833649367	0
2	FEN1	3.829423417	5.34E-62
2	H2AFZ	3.66930306	0
2	H1FX	3.517545592	4.90E-153
2	CCNA1	3.409993229	4.49E-82
2	GINS2	3.396930426	1.10E-79
2	H1F0	3.19482717	2.75E-34
2	LAMB3	3.115082928	6.25E-09
2	SMTN	3.093107474	2.84E-118
2	CDT1	3.027758472	9.85E-45
2	MCM10	3.002298977	6.68E-20
2	FAM110A	2.978904439	8.87E-31
2	MCM4	2.937612694	4.91E-44
2	TUBA1B	2.842737049	0
2	HELLS	2.80409216	5.99E-70
2	TUBA4A	2.626605734	5.51E-37
2	DTL	2.592002906	8.58E-14
2	GADD45G	2.585162811	1.94E-08
2	FST	2.567483119	4.42E-76
2	ID1	2.564124261	6.40E-135
2	LRRC17	2.559511031	4.13E-14
2	GATA3	2.53907796	0.001080188
2	TMEM158	2.480726391	6.87E-53
2	SNAPC1	2.45194185	1.85E-22

2	SERPIND1	2.446974964	4.28E-58
2	TNFRSF12A	2.442235458	9.63E-188
2	DKK1	2.437568488	4.61E-124
2	CRNDE	2.394422844	2.33E-19
2	TWIST1	2.383123551	2.29E-12
2	SOCS1	2.3628338	4.71E-13
2	E2F1	2.35040726	1.93E-23
2	MCM5	2.330824679	8.37E-31
2	ADAMTS1	2.289524664	4.12E-28
2	ING2	2.271200387	5.98E-36
2	LMO7	2.259772105	5.30E-49
2	RGS4	2.252247285	2.40E-148
3	DTL	2.864467417	1.43E-25
3	MCM4	2.370515087	2.82E-43
3	MCM10	2.884836554	8.37E-26
3	TUBA1B	2.374727347	0
3	GINS2	2.519801484	3.60E-63
3	CDT1	2.22514212	5.08E-37
3	E2F1	2.985704965	1.41E-53
3	MCM5	2.339736103	5.60E-47
5	SGIP1	2.423753918	8.88E-11
5	ITGA10	2.317167759	5.66E-13
5	RASGRP3	2.68766549	6.67E-09
5	CXCR4	2.983789305	3.01E-52
5	NRP2	2.451044864	3.52E-35
5	TM4SF18	2.710871286	8.57E-18
5	TNFSF10	3.253200019	9.59E-08
5	ERV3-1	2.203230129	2.29E-27
5	CSGALNACT1	2.985941539	1.18E-24
5	ADM	2.425491869	2.29E-19
5	TP53I11	2.368691783	5.30E-35
5	CHST1	2.363608325	2.66E-16
5	DEPP1	2.676648883	1.10E-38
5	VWF	2.261639305	2.53E-24
5	RGCC	2.553964856	1.94E-59
5	NID2	2.363361962	0.007881121
5	PGF	2.478637755	9.81E-120
5	CCL14	2.641992743	1.25E-05
5	PPP1R16B	3.177574014	5.81E-16
5	SULF2	2.544644983	4.64E-30
5	SOX18	2.363148604	2.44E-22
5	PLA2G4C	2.442865566	1.68E-14
5	CLDN5	2.225915281	1.06E-30
6	CHST1	12.52724927	3.06E-162
6	DEPP1	10.92164641	2.61E-257
6	CXCR4	9.002071006	9.68E-288
6	RASGRP3	8.64920468	3.82E-69

6	VWF	7.821002651	6.27E-191
6	PPP1R16B	7.191421822	2.63E-73
6	NID2	6.666894279	3.47E-26
6	CSGALNACT1	6.174138021	2.42E-106
6	HOXB5	5.980496397	4.69E-14
6	TNFSF10	5.196597822	1.09E-26
6	CCL14	4.974254146	2.44E-30
6	RGCC	4.930352466	2.12E-250
6	FABP5	4.866622997	0
6	SOX18	4.634071035	3.56E-130
6	CD200	4.380493155	2.72E-15
6	TP53I11	4.34493474	1.64E-162
6	ADM	4.309601744	6.21E-92
6	TM4SF18	4.216268854	3.34E-59
6	PGF	4.213154512	0
6	VASH1	4.172720907	1.18E-64
6	TLL1	4.16782646	1.22E-10
6	HLX	4.145280259	5.49E-44
6	SULF2	4.123017833	1.40E-114
6	MGP	4.022025635	2.84E-62
6	ITGA10	3.956226396	5.73E-57
6	TCIM	3.817541356	1.34E-09
6	PLA2G4C	3.691573248	2.86E-61
6	CLDN5	3.654669948	5.00E-129
6	TNFRSF21	3.57104183	5.12E-95
6	C2CD4B	3.423281917	0.000222799
6	KDR	3.369833974	1.41E-150
6	EFNB2	3.352259127	1.46E-88
6	RASSF2	3.322180512	1.21E-68
6	EFNA1	3.246846082	3.31E-46
6	KIAA0040	3.191152218	1.86E-51
6	RHOB	3.095560451	1.36E-188
6	ERV3-1	3.037873233	4.52E-97
6	PIM3	3.022200039	2.66E-131
6	NUDT4	3.007317617	6.56E-281
6	JAG2	3.006342172	1.24E-34
6	COL4A1	2.983246766	1.02E-112
6	YPEL2	2.968089543	2.31E-53
6	GCHFR	2.955487003	8.94E-47
6	PDGFB	2.908218609	6.29E-44
6	ANGPT2	2.739156846	8.86E-90
6	CDC42EP5	2.736282382	1.27E-71
6	IGFBP4	2.728311071	9.47E-165
6	CDC42EP3	2.693742432	7.14E-140
6	AFAP1L1	2.687274903	7.60E-73
6	EIF2B2	2.684204776	1.69E-103
6	FAM89A	2.659372929	1.98E-39

6	GMFG	2.62888396	2.40E-43
6	SOCS3	2.536327828	9.87E-22
6	NOTCH4	2.493742217	6.03E-57
6	NRP2	2.311014051	5.37E-75
6	TFPI2	2.304438529	4.10E-56
6	PECAM1	2.27617972	3.47E-243
7	PLA2G4C	3.152371554	8.22E-20
7	ITGA10	3.121176978	2.22E-19
7	CCL14	3.023824847	1.09E-06
7	FRMD3	2.987223157	1.90E-13
7	AKAP12	2.924917691	1.01E-52
7	CNTNAP3B	2.845281525	3.06E-54
7	CDC42EP5	2.82167109	3.26E-33
7	HSPG2	2.7813723	6.94E-121
7	PGF	2.779502026	2.09E-124
7	CAMK2N1	2.771007913	1.09E-19
7	NRP2	2.744249521	5.04E-45
7	CLDN5	2.721605786	3.77E-37
7	PTGS1	2.697830514	4.39E-19
7	AFAP1L1	2.694155741	3.38E-34
7	COX7A1	2.687075127	4.01E-11
7	CYTL1	2.66607973	2.22E-50
7	TFPI2	2.661226768	7.12E-32
7	YPEL2	2.658323565	3.37E-19
7	NEAT1	2.626352256	8.76E-134
7	CSGALNACT1	2.582747729	1.28E-14
7	JAG2	2.575164701	1.58E-12
7	ADAMTS4	2.564317592	3.61E-18
7	COL6A1	2.560476992	6.05E-14
7	PMP22	2.553594412	1.24E-42
7	SOX18	2.550497748	9.53E-23
7	PEG10	2.549523135	2.28E-13
7	PXDN	2.549305089	4.33E-115
7	PIM3	2.527812477	3.72E-41
7	PECAM1	2.513200058	2.34E-135
7	MMP2	2.493374097	1.52E-95
7	TP53I11	2.492145229	7.69E-34
7	SULF2	2.490120283	2.20E-23
7	CLU	2.46725696	9.80E-27
7	ERV3-1	2.450853698	1.77E-29
7	KDR	2.449796788	5.96E-39
7	ADAMTS18	2.431149044	3.84E-11
7	VASH1	2.416838783	1.95E-14
7	RALA	2.41390134	2.59E-159
7	RASSF2	2.399069881	6.10E-17
7	PMEPA1	2.39606253	4.64E-34
7	ADM	2.38519169	8.85E-16

7	KIAA0040	2.366222414	3.26E-13
7	GNG11	2.36468376	1.19E-194
7	RBM38	2.346378459	3.63E-11
7	PCDH9	2.345426729	2.47E-09
7	DUSP4	2.339396409	5.22E-39
7	DEPP1	2.329568787	5.93E-18
7	PDE2A	2.318380609	1.23E-05
7	SH3BP5	2.309915701	1.03E-37
7	LINC00607	2.304949891	1.57E-08
7	RASGRP3	2.295645951	7.14E-06
7	MMP1	2.243100367	8.48E-76
7	GYPC	2.241278206	1.14E-21
7	SPARC	2.223996187	4.53E-136
7	PPP1R16B	2.219993983	3.18E-06
7	TM4SF18	2.2062052	1.59E-08
8	PLAT	2.396676266	2.91E-47

Supplementary Table 6. Genes down-regulated in each PAH cluster relative to the other PAH clusters

Cluster	Gene	Fold-Change	q-Value
1	STMN1	0.244253345	0
1	NFIA-AS2	0.197067883	2.39E-08
1	PSRC1	0.104389099	9.40E-60
1	ITGA10	0.329523345	2.92E-50
1	HIST2H2AC	0.119157324	5.04E-51
1	CKS1B	0.15319777	0
1	ADAMTS4	0.37048192	1.11E-59
1	NUF2	0.018364749	1.90E-210
1	KIAA0040	0.284507257	5.97E-76
1	KIF14	0.118521253	3.01E-104
1	LAMB3	0.358514166	3.20E-09
1	NEK2	0.049607683	2.52E-97
1	DTL	0.186998721	1.80E-45
1	CENPF	0.080883382	0
1	HLX	0.280479802	2.64E-43
1	RRM2	0.07289685	0
1	RASGRP3	0.105837186	6.99E-80
1	CDC42EP3	0.405736117	3.12E-151
1	CKAP2L	0.03404495	1.79E-172
1	CXCR4	0.169763297	1.12E-253
1	SPC25	0.025091895	2.41E-152
1	NRP2	0.368023786	4.40E-119
1	SH3BP5	0.331468227	2.07E-206
1	SGO1	0.062273983	3.43E-178
1	KIF15	0.071535985	5.28E-67
1	CDCP1	0.448272833	1.09E-82
1	TMEM158	0.313194935	2.46E-157
1	H1FX	0.377973171	3.02E-170
1	TM4SF18	0.279478088	8.86E-58
1	SMC4	0.137068871	0
1	TNFSF10	0.3533631	1.71E-12
1	TACC3	0.15378618	1.69E-146
1	KDR	0.344126848	2.43E-159
1	H2AFZ	0.23023011	0
1	HMGB2	0.090317901	0
1	CENPU	0.156893809	3.11E-164
1	CENPK	0.158560365	4.97E-171
1	KIF20A	0.082908818	4.04E-74
1	AFAP1L1	0.32843076	5.57E-116
1	PTTG1	0.140760775	0
1	SPDL1	0.39294262	2.63E-48
1	MXD3	0.228220098	8.71E-59
1	HIST1H4C	0.156554348	2.51E-303
1	TCF19	0.152906713	7.91E-133

1	NOTCH4	0.331249772	1.50E-96
1	CENPW	0.160684586	0
1	FBXO5	0.149526215	1.42E-82
1	ANLN	0.072724406	0
1	DBF4	0.279615491	3.76E-86
1	TFPI2	0.401664531	1.37E-108
1	GNG11	0.445672441	0
1	PEG10	0.390406393	6.00E-42
1	LRRC17	0.355748386	2.56E-25
1	GIMAP4	0.272560825	3.82E-175
1	APLN	0.31799368	6.29E-294
1	ANGPT2	0.377331527	2.84E-109
1	CSGALNACT1	0.184666586	2.72E-107
1	STC1	0.384575941	1.39E-74
1	MCM4	0.264485843	4.34E-80
1	FABP5	0.193340702	0
1	ATAD2	0.179914595	8.23E-128
1	CKS2	0.246279916	0
1	PHF19	0.282101542	0.00E+00
1	SAPCD2	0.201217861	2.48E-105
1	TUBB4B	0.23817892	0
1	ADM	0.348805986	2.91E-64
1	KIF18A	0.121272543	3.09E-32
1	TP53I11	0.310252643	2.52E-140
1	CHST1	0.06741796	3.01E-212
1	FEN1	0.236146838	1.78E-84
1	GAL	0.430629818	5.34E-13
1	PDE2A	0.373912151	6.19E-23
1	H2AFX	0.319775861	5.24E-135
1	GATA3	0.247199186	3.72E-11
1	MCM10	0.174957828	5.01E-54
1	DEPP1	0.127185655	4.48E-273
1	HELLS	0.324206431	5.38E-101
1	MKI67	0.037549839	0
1	FOXM1	0.119881465	6.21E-260
1	VWF	0.237108429	4.72E-147
1	CDCA3	0.056564102	6.39E-145
1	CLEC2B	0.334221764	2.90E-102
1	APOLD1	0.327823824	1.07E-14
1	TUBA1B	0.262135441	0
1	TUBA1C	0.326656979	0
1	RACGAP1	0.106888771	5.73E-149
1	BRCA2	0.154227039	2.66E-62
1	RGCC	0.315868631	2.93E-213
1	CKAP2	0.281256787	2.98E-141
1	DIAPH3	0.173375804	1.85E-167
1	LMO7	0.303094297	6.38E-166

1	MIS18BP1	0.256096784	6.41E-120
1	NID2	0.328637985	1.35E-11
1	CDKN3	0.086289698	0
1	SYNE2	0.240246648	1.56E-102
1	PGF	0.221243926	0
1	EIF2B2	0.385737986	5.87E-157
1	VASH1	0.444174255	1.47E-31
1	ARHGAP11B	0.244473874	2.40E-26
1	ARHGAP11A	0.114481942	3.57E-161
1	KNSTRN	0.264271857	1.39E-75
1	GCHFR	0.375832891	1.17E-48
1	C2CD4B	0.159123135	4.05E-12
1	PCLAF	0.146871347	0
1	PRC1	0.065022398	1.79E-151
1	LINC01197	0.367031873	1.14E-09
1	CCNF	0.283161855	3.20E-77
1	SOCS1	0.260120299	1.53E-38
1	ARL6IP1	0.374777427	7.80E-152
1	ORC6	0.202380685	2.07E-97
1	CRNDE	0.419300909	3.52E-23
1	GINS2	0.167222252	9.07E-175
1	CDT1	0.22841749	3.80E-103
1	CCL14	0.294363516	4.74E-22
1	TOP2A	0.041084026	0
1	IGFBP4	0.437076627	9.24E-159
1	BRCA1	0.234659823	3.39E-104
1	KPNA2	0.308606522	1.55E-197
1	BIRC5	0.088253469	0
1	SOCS3	0.438216739	2.78E-20
1	TYMS	0.219361425	9.70E-306
1	CDC25B	0.397607087	3.97E-81
1	RASSF2	0.320235309	3.70E-76
1	CD93	0.259551879	2.23135855762574e-318
1	TPX2	0.07793896	0
1	E2F1	0.211355304	3.61E-83
1	PPP1R16B	0.220203735	5.95E-55
1	MYBL2	0.09814266	0
1	UBE2C	0.034104584	0
1	SULF2	0.353254339	1.74E-85
1	SOX18	0.231819789	7.31E-143
1	GMFG	0.143863811	2.12E-170
1	UBE2S	0.229769956	0
1	CLDN5	0.182762312	5.18E-257
1	MCM5	0.245424035	3.77E-100
1	H1F0	0.450354051	9.40E-24
2	SGIP1	0.250882449	1.22E-50
2	SELP	0.441578776	1.79E-05

2	RHOB	0.327880452	8.82E-199
2	LBH	0.446835666	1.94E-05
2	RASGRP3	0.239711593	4.13E-36
2	CXCR4	0.108679522	6.26131270424066e-316
2	LINC00607	0.381005555	1.03E-26
2	TNFSF10	0.417046621	7.15E-05
2	TLL1	0.359841111	2.04E-05
2	ERV3-1	0.358075837	4.49E-88
2	CSGALNACT1	0.292896905	1.65E-57
2	TCIM	0.410209792	0.000186551
2	CHST1	0.148705181	1.27E-120
2	DEPP1	0.119280671	1.05E-215
2	CCND2	0.356297391	1.25E-124
2	VWF	0.287912909	2.92E-103
2	RGCC	0.31189889	1.97E-197
2	DIO2	0.442976981	3.47E-09
2	JAG2	0.345721994	3.54E-34
2	CCL2	0.406788176	3.95E-59
2	CCL14	0.288895528	2.83E-20
2	HOXB5	0.149657879	3.67E-15
2	YPEL2	0.404290385	8.57E-40
2	PPP1R16B	0.167851581	1.91E-63
2	SOX18	0.423420696	4.09E-43
2	PLA2G4C	0.259959801	1.84E-67
2	PDGFB	0.174577656	8.36E-114
3	SGIP1	0.243221511	1.59E-73
3	ITGA10	0.355058544	2.28E-54
3	SELP	0.295148294	3.37E-17
3	PTGS2	0.332175163	2.27E-37
3	PLA2G4A	0.357057041	5.22E-44
3	BTG2	0.395684539	2.89E-61
3	HLX	0.434310128	6.82E-25
3	RHOB	0.277096848	0
3	LBH	0.352273771	1.74E-14
3	RASGRP3	0.146086134	4.48E-76
3	CXCR4	0.092409579	0
3	SLC40A1	0.289897283	5.62E-36
3	FN1	0.294780818	0
3	LINC00607	0.243678097	2.66E-73
3	COL8A1	0.442520585	4.96E-14
3	CD200	0.452923419	8.39E-07
3	TM4SF18	0.360164703	3.31E-46
3	TNFSF10	0.182521378	5.89E-37
3	KDR	0.452503657	2.19E-112
3	TLL1	0.182371819	4.47E-19
3	CDKN1A	0.39674739	1.52E-277
3	TNFRSF21	0.41193963	2.50E-76

3	ERV3-1	0.290012756	1.76E-176
3	SAT1	0.405127744	0
3	BGN	0.431677946	1.80E-149
3	CSGALNACT1	0.197108022	1.22E-119
3	TCIM	0.221802004	3.19E-17
3	CTHRC1	0.412258468	2.45E-09
3	ALDH1A1	0.430769556	1.15E-24
3	PTGS1	0.45374134	3.91E-45
3	ADM	0.302472709	3.44E-99
3	TP53I11	0.308219116	8.71E-173
3	CHST1	0.081917518	1.21E-208
3	NEAT1	0.292132568	0
3	DEPP1	0.054976754	0
3	ACTA2	0.362391833	1.49E-53
3	FAS	0.375564598	2.40E-13
3	VWF	0.163592253	9.54E-244
3	MGP	0.285287926	1.59E-99
3	MDM2	0.404053425	2.52E-186
3	NUDT4	0.423042238	1.29E-290
3	NUAK1	0.450921749	2.84E-59
3	RGCC	0.30702492	4.00E-264
3	PCDH17	0.447904877	7.75E-08
3	KCTD12	0.425159913	2.55E-281
3	EFNB2	0.452124049	9.02E-65
3	COL4A1	0.404789291	8.59E-122
3	NID2	0.193545764	3.39E-28
3	PGF	0.380687239	0
3	VASH1	0.353684478	7.04E-64
3	DIO2	0.333435601	1.46E-24
3	JAG2	0.413428497	2.85E-36
3	MMP2	0.36389727	0
3	CCL2	0.201859698	1.49E-248
3	CCL14	0.249688552	6.05E-32
3	HOXB5	0.188344392	6.40E-16
3	YPEL2	0.295524501	3.87E-95
3	PPP1R16B	0.119016373	3.06E-103
3	SULF2	0.340703554	8.48E-111
3	SOX18	0.433620213	1.91E-62
3	GDF15	0.446934669	1.23E-125
3	PLA2G4C	0.11948139	2.51E-184
3	CDC42EP5	0.423626673	1.44E-91
3	PDGFB	0.350503551	2.03E-63
4	CAMK2N1	0.314542739	9.54E-50
4	STMN1	0.284817679	0
4	NFIA-AS2	0.070722898	3.35E-14
4	PSRC1	0.079503929	2.14E-56
4	ITGA10	0.188964746	2.61E-73

4	HIST2H2AC	0.117412528	2.25E-40
4	CKS1B	0.243914478	2.14E-280
4	ADAMTS4	0.205664039	3.60E-100
4	NUF2	0.081932579	1.20E-121
4	KIAA0040	0.236566858	1.22E-71
4	KIF14	0.125244956	8.11E-78
4	LAMB3	0.403843821	0.000274213
4	NEK2	0.065357713	5.36E-75
4	DTL	0.274691836	1.18E-20
4	CENPF	0.046021737	0
4	HLX	0.345937557	1.57E-20
4	FAM89A	0.374497541	2.45E-30
4	RRM2	0.13482934	2.19E-186
4	RASGRP3	0.137345918	3.01E-53
4	CDC42EP3	0.376208384	1.26E-114
4	CKAP2L	0.055052347	8.05E-132
4	GYPC	0.448294706	6.82E-61
4	CXCR4	0.213915682	1.01E-149
4	SPC25	0.055746284	3.98E-110
4	NRP2	0.336314719	1.33E-103
4	TUBA4A	0.368787503	1.87E-34
4	SH3BP5	0.289472012	4.08E-181
4	SGO1	0.121662499	1.45E-102
4	KIF15	0.065697527	1.24E-58
4	CDCP1	0.393007597	2.41E-64
4	TMEM158	0.331244034	7.50E-104
4	H1FX	0.346210566	3.40E-136
4	TM4SF18	0.309333981	7.39E-34
4	SMC4	0.1675204	3.02E-268
4	CLDN11	0.422900923	1.70E-82
4	TNFSF10	0.194997291	7.09E-22
4	TACC3	0.165009001	3.75E-100
4	KDR	0.289914227	3.25E-142
4	H2AFZ	0.302881971	0
4	HMGB2	0.142284076	0
4	CENPU	0.125055582	2.00E-159
4	CENPK	0.131852081	2.04E-146
4	KIF20A	0.045488968	1.13E-80
4	AFAP1L1	0.242492481	8.93E-131
4	PTTG1	0.197170192	0
4	SPDL1	0.369108796	5.80E-38
4	MXD3	0.144807835	2.26E-68
4	HIST1H4C	0.123643808	0
4	TCF19	0.121368645	4.10E-122
4	NOTCH4	0.386509885	8.30E-48
4	CENPW	0.207071995	3.95252516672997e-321
4	FBXO5	0.210597074	2.10E-43

4	TWIST1	0.393539987	3.01E-13
4	ANLN	0.09129931	4.47E-220
4	DBF4	0.207687443	6.12E-89
4	TFPI2	0.353340256	1.07E-90
4	GNG11	0.452973932	0
4	PEG10	0.388022065	2.74E-26
4	LRRC17	0.29973351	4.03E-25
4	GIMAP4	0.291226973	9.92E-112
4	APLN	0.402474661	1.62E-137
4	ANGPT2	0.358622593	4.27E-82
4	CSGALNACT1	0.208576673	5.23E-73
4	STC1	0.340079007	1.69E-65
4	DUSP4	0.42059115	8.28E-82
4	MCM4	0.209768359	6.41E-76
4	FABP5	0.386417245	4.27E-260
4	ATAD2	0.217699321	7.96E-73
4	MSMP	0.341596696	1.38E-81
4	CKS2	0.291553871	1.01E-290
4	GADD45G	0.396706116	2.46E-06
4	GABBR2	0.399035516	2.15E-96
4	PHF19	0.293790369	6.53E-200
4	SAPCD2	0.196000072	7.94E-80
4	TUBB4B	0.176882861	0
4	ADM	0.270106182	2.73E-62
4	KIF18A	0.110840565	1.40E-26
4	TP53I11	0.253073123	2.35E-140
4	CHST1	0.090363464	1.30E-155
4	FEN1	0.224970168	3.12E-61
4	PDE2A	0.195293382	9.68E-40
4	H2AFX	0.292441644	5.25E-105
4	GATA3	0.161308397	6.40E-13
4	MCM10	0.217202049	1.51E-30
4	DEPP1	0.082077324	1.08E-287
4	HELLS	0.352737035	6.07E-57
4	MKI67	0.036253017	0
4	FOXM1	0.131226955	1.12E-196
4	VWF	0.290245967	4.80E-88
4	CDCA3	0.078249372	8.40E-105
4	CLEC2B	0.345183946	3.26E-66
4	APOLD1	0.327541532	1.14E-09
4	TUBA1B	0.128324957	0
4	TUBA1C	0.26092127	0
4	RACGAP1	0.184697031	2.07E-69
4	BRCA2	0.198961876	3.70E-40
4	CKAP2	0.289707374	2.25E-93
4	PCDH17	0.407865219	2.62E-05
4	DIAPH3	0.193633459	3.11E-109

4	LMO7	0.203668157	1.37E-229
4	MIS18BP1	0.333575091	1.10E-55
4	NID2	0.105006851	1.01E-29
4	CDKN3	0.129756632	8.35E-303
4	SYNE2	0.169590315	2.23E-106
4	PGF	0.247823195	0
4	VASH1	0.312368866	7.81E-44
4	ARHGAP11B	0.202026263	2.72E-23
4	ARHGAP11A	0.086663132	7.19E-147
4	KNSTRN	0.30479505	1.75E-41
4	GCHFR	0.347774984	1.35E-37
4	PCLAF	0.223081744	0
4	PRC1	0.169229792	3.79E-63
4	LINC01197	0.180757707	1.66E-18
4	CCNF	0.227830399	7.80E-73
4	SOCS1	0.285183538	1.94E-24
4	ARL6IP1	0.409313837	2.49E-90
4	ORC6	0.216313466	1.28E-61
4	CRNDE	0.334381757	9.20E-24
4	GINS2	0.223917281	1.28E-95
4	CDT1	0.235612748	6.63E-73
4	CCL14	0.264624596	9.57E-18
4	TOP2A	0.06869916	1.44E-253
4	IGFBP4	0.442247776	1.52E-105
4	BRCA1	0.150856271	5.44E-116
4	HOXB5	0.180511778	4.37E-10
4	YPEL2	0.441212104	8.26E-24
4	KPNA2	0.340292223	1.27E-122
4	BIRC5	0.106406943	0
4	TYMS	0.232527221	6.51E-215
4	CDC25B	0.255841046	2.12E-121
4	RASSF2	0.346714695	5.68E-44
4	THBD	0.349079734	2.38E-74
4	CD93	0.251301218	2.16E-245
4	TPX2	0.082223039	0
4	E2F1	0.203366273	4.36E-62
4	PPP1R16B	0.345510824	1.13E-19
4	MYBL2	0.087687886	2.67E-303
4	UBE2C	0.081496411	3.86E-297
4	SULF2	0.356762895	5.56E-57
4	SOX18	0.23501839	4.30E-95
4	GMFG	0.257679199	8.88E-69
4	CDC42EP5	0.416158131	1.76E-52
4	UBE2S	0.245206574	0
4	CLDN5	0.200605812	1.21E-168
4	SMTN	0.370380765	1.74E-109
4	MCM5	0.25360084	2.80E-73

4	PIM3	0.297572839	6.75E-146
5	NEXN	0.301775435	5.40E-62
5	PSRC1	0.046452826	2.72E-64
5	HIST2H2AC	0.298127601	1.68E-10
5	CKS1B	0.185936274	3.67821992015891e-319
5	RGS4	0.118911294	0
5	NUF2	0.029876782	8.60E-166
5	KIF14	0.097461446	3.75E-77
5	LAMB3	0.349758638	8.25E-05
5	NEK2	0.019084529	1.87E-103
5	DTL	0.178621387	9.51E-28
5	CENPF	0.062239978	0
5	RRM2	0.071331534	2.55E-296
5	IL1RL1	0.246752152	1.18E-61
5	CKAP2L	0	1.38E-211
5	SPC25	0.027173089	4.17E-128
5	SGO1	0.055911405	3.63E-141
5	KIF15	0.120769283	1.85E-32
5	SEMA3F	0.44079943	8.69E-58
5	VGLL3	0.411616017	9.96E-09
5	DCBLD2	0.450246646	1.08E-52
5	ALCAM	0.433626576	9.51E-24
5	PLOD2	0.438001197	1.66E-74
5	PTX3	0.21847534	4.93E-249
5	SMC4	0.205820964	4.33E-180
5	MELTF	0.448033569	0.000347933
5	TACC3	0.202389142	3.12E-62
5	CXCL1	0.271161799	8.34E-122
5	CXCL3	0.302221737	7.77E-17
5	H2AFZ	0.264724112	0
5	DDIT4L	0.372201125	5.20E-26
5	MGARP	0.212127245	1.09E-105
5	HMGB2	0.167159721	0.00E+00
5	CENPU	0.051149343	2.75E-214
5	FST	0.137082666	1.31E-246
5	CENPK	0.138583929	4.71E-109
5	KIF20A	0.04444927	1.13E-71
5	PTTG1	0.12706919	0
5	SPDL1	0.43014868	1.52E-19
5	STC2	0.318002774	4.70E-46
5	MXD3	0.236609207	2.22E-31
5	HIST1H4C	0.260847864	2.29E-182
5	TCF19	0.133205949	6.40E-95
5	CENPW	0.146172848	0
5	RSPO3	0.19403935	4.42E-22
5	FBXO5	0.159609423	5.01E-46
5	ANLN	0.035491381	0

5	INHBA	0.373051361	1.37E-64
5	SEMA3C	0.119421643	4.69E-70
5	SEMA3A	0.29113285	7.91E-27
5	DBF4	0.217384709	4.97E-62
5	LRRC17	0.426688202	8.42E-10
5	EPHB6	0.268410701	1.26E-06
5	PLAT	0.368916068	9.03E-85
5	MCM4	0.190026048	1.30E-62
5	ATAD2	0.127454101	1.60E-106
5	CKS2	0.232880362	0
5	GADD45G	0.382916184	6.12E-05
5	TBC1D2	0.266638825	1.69E-52
5	PHF19	0.292490037	3.61E-154
5	CLIC3	0.24755554	2.62E-14
5	SAPCD2	0.232393453	3.96E-50
5	TUBB4B	0.323944032	5.44E-222
5	BDNF	0.138622133	2.87E-57
5	KIF18A	0.080887333	1.65E-27
5	FEN1	0.212906507	1.23E-53
5	GAL	0.453056869	0.00013991
5	TAGLN	0.250267923	3.92E-37
5	H2AFX	0.363077647	7.53E-57
5	MCM10	0.016425763	1.09E-118
5	DKK1	0.131353346	0
5	ANKRD1	0.210860767	9.42E-222
5	HELLS	0.258504387	8.44E-67
5	MKI67	0.027769703	0
5	FOXM1	0.049747156	7.95E-304
5	CDCA3	0.063586021	3.87E-100
5	GPRC5A	0.191228672	2.84E-147
5	TUBA1B	0.132808222	0
5	TUBA1C	0.326670578	2.65E-305
5	RACGAP1	0.055245353	1.36E-147
5	KRT7	0.316309329	3.45E-272
5	BRCA2	0.183060807	2.48E-34
5	CCNA1	0.307979106	2.17E-51
5	CKAP2	0.340380899	5.05E-50
5	DIAPH3	0.053778977	7.90E-236
5	MIS18BP1	0.429130978	2.40E-24
5	CDKN3	0.067438785	0
5	ARHGAP11B	0.214452738	1.88E-16
5	ARHGAP11A	0.067066734	1.69E-151
5	KNSTRN	0.286773703	2.50E-35
5	PCLAF	0.090886968	0
5	PRC1	0.058358311	4.44E-115
5	CCNF	0.34105871	1.32E-29
5	TNFRSF12A	0.407235364	6.94E-114

5	ORC6	0.152699503	4.69E-70
5	MT2A	0.325967247	0
5	MT1E	0.242867385	1.18E-161
5	GINS2	0.14476714	1.27E-125
5	CDT1	0.280547818	5.11E-41
5	TOP2A	0.023755187	0
5	BRCA1	0.227237589	5.99E-65
5	BIRC5	0.048442275	0
5	TYMS	0.389406606	1.21E-59
5	SERPINB2	0.178783686	2.26E-30
5	CDC25B	0.443986093	1.43E-34
5	TPX2	0.054255348	0
5	E2F1	0.203351348	2.39E-43
5	MYBL2	0.047996624	0
5	UBE2C	0.033974655	0
5	UBE2S	0.241700947	0
5	TXNRD2	0.414829498	3.28E-77
5	SERPIND1	0.109283431	0
5	MCM5	0.354680314	5.80E-31
5	ADAMTS1	0.246626239	2.54E-44
6	S100A4	0.38718585	1.14E-08
6	CKS1B	0.150851402	0
6	RGS4	0.091851173	0
6	RGS5	0.373267983	1.02E-161
6	NUF2	0.040168888	2.23E-180
6	KIF14	0.177698027	1.17E-70
6	LAMB3	0.283000562	4.04E-13
6	NEK2	0.060108263	6.06E-88
6	DTL	0.124880097	1.06E-57
6	CENPF	0.043245011	0
6	RRM2	0.057262541	0
6	IL1RL1	0.114882144	3.14E-186
6	CKAP2L	0.036064958	4.98E-166
6	LYPD1	0.294876479	1.42E-228
6	SPC25	0.030403158	2.20E-144
6	SERPINE2	0.394318444	3.65E-81
6	SGO1	0.037499949	2.12E-204
6	KIF15	0.075711278	1.73E-62
6	CDCP1	0.303914586	9.23E-155
6	TMEM158	0.339548396	2.73E-91
6	SEMA3F	0.260907624	5.21E-267
6	VGLL3	0.427108342	1.58E-14
6	DCBLD2	0.258875137	7.78E-283
6	ALCAM	0.371397279	2.62E-61
6	PLOD2	0.346643627	7.01E-218
6	PTX3	0.114303883	0
6	SMC4	0.211059151	1.30E-276

6	CLDN11	0.194086805	0
6	MELTF	0.344309176	2.92E-15
6	TACC3	0.117027801	1.05E-165
6	HOPX	0.365818181	6.50E-185
6	CXCL8	0.377670462	1.01E-97
6	CXCL1	0.229329243	1.15E-239
6	CXCL3	0.340905695	1.10E-28
6	H2AFZ	0.302618704	0
6	DDIT4L	0.295078027	3.36E-67
6	FGF2	0.402960578	1.99E-18
6	MGARP	0.136356433	1.32E-260
6	HHIP	0.410671083	0
6	HMGB2	0.147831985	0
6	CENPU	0.094920669	2.09E-219
6	FST	0.085863441	0
6	CENPK	0.141593373	1.83E-169
6	TGFBI	0.308930996	3.87E-68
6	KIF20A	0.059887378	2.55E-81
6	PTTG1	0.134087196	0
6	SPDL1	0.334176497	7.76E-58
6	STC2	0.273863399	2.87E-105
6	MXD3	0.154277138	8.66E-79
6	HIST1H4C	0.193799973	2.50E-266
6	TCF19	0.10308413	5.63E-165
6	CENPW	0.159908558	0
6	RSPO3	0.175624465	2.22E-45
6	SGK1	0.221121729	4.44E-252
6	FBXO5	0.243942037	2.74E-45
6	TWIST1	0.346084092	3.13E-23
6	ANLN	0.061823986	0
6	INHBA	0.206944161	7.56E-265
6	SEMA3C	0.10750346	9.58E-115
6	SEMA3A	0.15007401	1.60E-114
6	DBF4	0.237394802	7.41E-96
6	LRRC17	0.15492123	4.10E-69
6	CALD1	0.432210324	0
6	EPHB6	0.181412089	7.28E-17
6	INSIG1	0.401916346	1.29E-44
6	ZNF703	0.386023628	4.47E-54
6	SFRP1	0.397296256	1.34E-87
6	PLAT	0.144142913	0
6	MCM4	0.190930625	1.51E-113
6	ATAD2	0.117879585	1.19E-162
6	LY6K	0.293698045	3.15E-40
6	PRSS3	0.345735308	0
6	CKS2	0.174051822	0
6	GADD45G	0.200268543	1.62E-20

6	TBC1D2	0.176084847	1.53E-142
6	GABBR2	0.31223015	1.10E-205
6	PHF19	0.288051551	1.99E-257
6	CLIC3	0.121731859	1.59E-48
6	SAPCD2	0.240139982	5.24E-79
6	TUBB4B	0.271548088	0
6	BDNF	0.142392771	2.61E-87
6	KIF18A	0.017995144	7.44E-59
6	FEN1	0.200628092	7.40E-93
6	GAL	0.408671242	4.61E-12
6	BIRC3	0.348242592	6.34E-14
6	TAGLN	0.149803286	6.46E-146
6	H2AFX	0.349827148	1.38E-107
6	MCM10	0.081590888	5.21E-82
6	NMT2	0.4112734	7.03E-184
6	DKK1	0.09851588	0
6	PLAC9	0.367759827	1.11E-141
6	ADIRF	0.352013853	0
6	ANKRD1	0.088405749	0
6	HELLS	0.23707667	8.28E-131
6	MKI67	0.020500204	0
6	FOXM1	0.089758913	8.68E-298
6	CDCA3	0.097542996	4.69E-108
6	APOLD1	0.335191247	1.01E-12
6	GPRC5A	0.128211094	0
6	MGST1	0.376398593	1.04E-198
6	TUBA1B	0.190339314	0
6	TUBA1C	0.287456318	0
6	RACGAP1	0.057828708	2.64E-188
6	KRT7	0.241060475	0
6	RAB3IP	0.374316255	5.07E-88
6	GLIPR1	0.401547615	1.11E-89
6	KITLG	0.339656211	4.66E-60
6	BRCA2	0.137352319	7.27E-68
6	CCNA1	0.238781724	1.22E-131
6	CKAP2	0.261865417	1.05E-141
6	DIAPH3	0.055474542	1.59E-291
6	MIS18BP1	0.3126335	3.04E-82
6	CDKN3	0.055395501	0
6	SNAPC1	0.420274852	2.13E-32
6	ARHGAP11B	0.181884517	1.88E-33
6	ARHGAP11A	0.094065528	5.85E-170
6	KNSTRN	0.320210929	1.01E-51
6	MAP1A	0.404676181	4.29E-26
6	TPM1	0.426904475	0
6	PCLAF	0.092455901	0
6	PRC1	0.094783585	4.13E-116

6	ALDH1A3	0.451979692	2.30E-88
6	CCNF	0.220585642	3.20E-95
6	TNFRSF12A	0.258639764	0
6	ORC6	0.141707599	3.90E-122
6	MT2A	0.245932403	0
6	MT1E	0.193032508	0
6	CDH11	0.300263449	1.81E-98
6	OSGIN1	0.410159598	1.58E-13
6	GINS2	0.109941423	2.38E-225
6	CDT1	0.195628401	4.02E-110
6	TOP2A	0.028348515	0
6	BRCA1	0.171741438	1.54E-135
6	ITGB3	0.378052131	1.98E-59
6	KPNA2	0.440838622	5.11E-95
6	BIRC5	0.067945747	0
6	TYMS	0.220874857	6.13E-277
6	SERPINB2	0.184332685	4.23E-42
6	DSEL	0.449530726	3.74E-46
6	FAM110A	0.396527897	7.72E-23
6	CDC25B	0.326222137	2.15E-111
6	ID1	0.408304669	5.22E-149
6	TPX2	0.057371806	0
6	E2F1	0.164484268	4.27E-97
6	MYL9	0.387308338	7.59E-41
6	MYBL2	0.054547131	0
6	UBE2C	0.042418477	0
6	FSTL3	0.350152668	4.40E-42
6	GADD45B	0.261113828	1.40E-67
6	UBE2S	0.237208444	0
6	TXNRD2	0.26840233	4.78E-301
6	SERPIND1	0.072009226	0
6	MCM5	0.300108178	7.80E-73
6	ADAMTS1	0.133197969	8.74E-150
7	UBE2C	0.380797062	9.03E-41
7	MKI67	0.408309392	9.37E-41
7	TOP2A	0.398434073	1.81E-31
7	SPC25	0.320524152	3.82E-13
7	SGO1	0.382398869	5.96E-13
7	NUF2	0.384675808	1.07E-11
7	CDCA3	0.379262066	2.35E-10
7	KIF15	0.327834352	1.71E-07
8	HIST2H2AC	0.367749631	5.62E-10
8	NUF2	0.29821942	2.36E-34
8	KIF14	0.349216137	2.01E-22
8	NEK2	0.159155243	1.24E-41
8	CENPF	0.215615536	3.05E-174
8	RRM2	0.327047183	1.79E-83

8	RASGRP3	0.192219499	6.66E-40
8	CKAP2L	0.207588401	6.62E-52
8	CXCR4	0.205793397	1.12E-159
8	SPC25	0.201290675	3.26E-45
8	SGO1	0.271671988	1.31E-45
8	KIF15	0.264585824	6.38E-18
8	TM4SF18	0.40920111	2.86E-20
8	SMC4	0.389565682	5.79E-89
8	HMGB2	0.295809071	5.82E-176
8	KIF20A	0.363931335	6.00E-11
8	MXD3	0.327367757	2.66E-26
8	HIST1H4C	0.376867637	1.61E-87
8	ANLN	0.387737352	1.43E-46
8	CSGALNACT1	0.300788937	1.36E-39
8	FABP5	0.438368593	8.62E-208
8	KIF18A	0.207763877	6.04E-15
8	CHST1	0.12615124	3.13E-132
8	DEPP1	0.105882777	1.12E-244
8	MKI67	0.183166544	6.00E-219
8	VWF	0.31200514	7.18E-65
8	CDCA3	0.271739949	1.35E-32
8	CKAP2	0.450036104	2.66E-43
8	NID2	0.37620102	2.29E-05
8	CDKN3	0.443349513	7.64E-55
8	ARHGAP11B	0.43715258	2.84E-06
8	ARHGAP11A	0.295018982	4.39E-48
8	PRC1	0.422759465	2.61E-14
8	CCL14	0.421720005	2.03E-06
8	TOP2A	0.193707842	9.84E-139
8	BIRC5	0.349651403	8.53E-103
8	TPX2	0.263415976	4.44E-144
8	PPP1R16B	0.20771044	8.11E-44
8	UBE2C	0.227206971	1.25E-131
8	SOX18	0.420091139	1.17E-37
8	GMFG	0.449757403	7.16E-22
